# COPS: A Sensitive and Accurate Tool for Detecting Somatic Copy Number Alterations Using Short-Read Sequence Data from Paired Samples

**DOI:** 10.1371/journal.pone.0047812

**Published:** 2012-10-22

**Authors:** Neeraja M. Krishnan, Prakhar Gaur, Rakshit Chaudhary, Arjun A. Rao, Binay Panda

**Affiliations:** 1 Ganit Labs, Bio-IT Centre, Institute of Bioinformatics and Applied Biotechnology, Bangalore, India; 2 Strand Life Sciences, Bangalore, India; Seoul National University College of Medicine, Republic of Korea

## Abstract

Copy Number Alterations (CNAs) such as deletions and duplications; compose a larger percentage of genetic variations than single nucleotide polymorphisms or other structural variations in cancer genomes that undergo major chromosomal re-arrangements. It is, therefore, imperative to identify cancer-specific somatic copy number alterations (SCNAs), with respect to matched normal tissue, in order to understand their association with the disease. We have devised an accurate, sensitive, and easy-to-use tool, COPS, COpy number using Paired Samples, for detecting SCNAs. We rigorously tested the performance of COPS using short sequence simulated reads at various sizes and coverage of SCNAs, read depths, read lengths and also with real tumor:normal paired samples. We found COPS to perform better in comparison to other known SCNA detection tools for all evaluated parameters, namely, sensitivity (detection of true positives), specificity (detection of false positives) and size accuracy. COPS performed well for sequencing reads of all lengths when used with most upstream read alignment tools. Additionally, by incorporating a downstream boundary segmentation detection tool, the accuracy of SCNA boundaries was further improved. Here, we report an accurate, sensitive and easy to use tool in detecting cancer-specific SCNAs using short-read sequence data. In addition to cancer, COPS can be used for any disease as long as sequence reads from both disease and normal samples from the same individual are available. An added boundary segmentation detection module makes COPS detected SCNA boundaries more specific for the samples studied. COPS is available at ftp://115.119.160.213 with username “cops” and password “cops”.

## Introduction

Copy number alterations (CNAs) represent an important category of structural aberrations in human cancers [1]–[3], where the genome undergoes amplifications and/or deletions on a very large-scale [4], [5]. As against single nucleotide polymorphisms (SNPs), which impact the chromosome (chr) at a single nucleotide level, CNAs range from one kilo base (kb) to several mega bases (mb) [6] and therefore, may span across several genes [7], including oncogenes and tumor-suppressor genes [8]–[11]. In some diseases other than cancer, copy number variations (CNVs) severely impact cellular function, for e.g. in the case of DiGeorge/velocardiofacial syndrome [12], the autosomal dominant Prader-Willi syndrome [13], the Williams-Beuren syndrome [14] and the Smith-Magenis syndrome [15]. In addition to cancer/disease genomes, genomes of normal individuals also show copy number variations (CNVs) [16], [17]. In cancer, CNAs usually refer to somatic variations present in tumor genomes compared to normal genomes from the same individual (matched normal). Hence, it is important to identify the cancer-specific somatic CNAs (SCNAs) and distinguish them from those inherited or present in matched normal samples (germ line CNVs). Further more, given the heterogeneity in CNVs in the normal population, [18] it is imperative to distinguish CNAs or SCNAs (detected using matched control samples) from CNVs (detected using a single sample) in a given disease/cancer genome.

High throughput DNA microarray and next generation sequencing (NGS) based approaches have been used in the past in detecting structural variations in human genomes [19]–[30]. The NGS-based methods provide an unbiased and comprehensive view of all types of variations in the genome, such as SNPs, short indels, translocations and CNVs [31], [32] where finding the alterations does not depend on indirect measurements of probe intensities as in the case of DNA microarrays [19]. Often, data from high-resolution array comparative genome hybridization (array-CGH) [21] is combined with whole genome sequencing to obtain a comprehensive map of CNVs existing in a population [31].

A number of open-source/freely-available algorithms have been reported in the literature for CNV detection using data from next-generation sequencers [29], [33]–[37]. However, most CNV detection tools are optimized to detect amplifications and deletions of a selected size range in a single sample (either cancer or normal) and results from various tools are often not comparable to each other. Similarly, the available pair-wise tools do not provide good sensitivity of detection and ease of use. Hence, there is a need to develop an easy-to-use, sensitive, accurate tool to detect SCNAs using paired samples.

Here, we report an accurate, sensitive, and easy-to-use SCNA detection tool, COPS, (COpy number using Paired Samples) and a downstream boundary segmentation detection module. We have evaluated sensitivity (detection of true positives), specificity (detection of false positives), size-deviation (variation from the actual SCNA size) and processing times (time taken to use the tool) of COPS using both simulated and real tumor:normal datasets. We tested COPS using simulated sequencing data with different read lengths and coverage, in combination with different alignment tools to optimize its working over a wide-range of conditions. We find COPS to perform well in comparison with commonly used tools for detection of CNA for all evaluated parameters at a maximum resolution of 500 nucleotides. Precise SCNA boundaries were further fine-tuned using an additional boundary segmentation module.

## Results

### COPS Workflow

**Figure 1:**
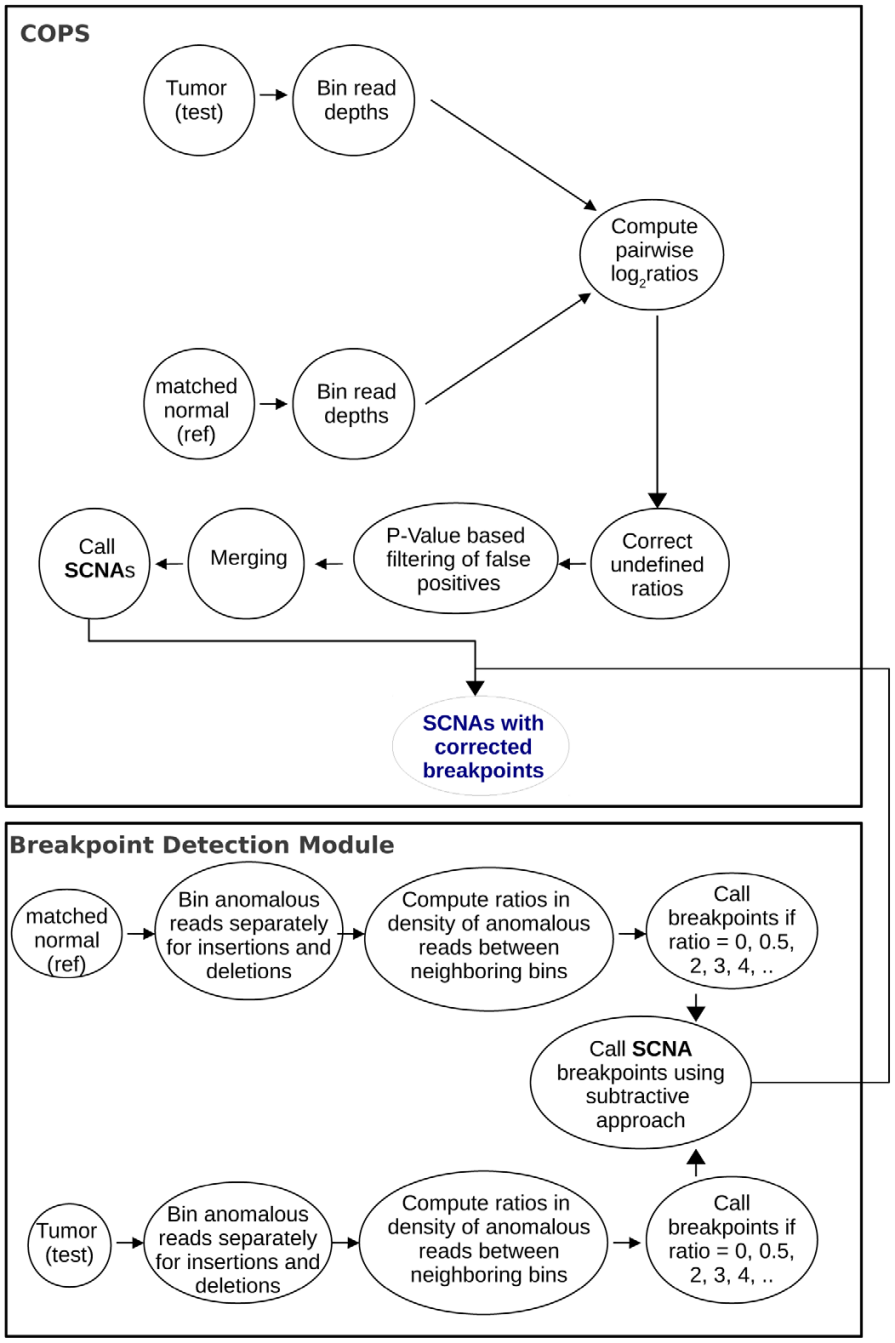
COPS and the boundary segmentation module workflow. Steps involved in the COPS workflow for tumor:normal (paired) samples along with the steps involved in the SCNA boundary segmentation module.

The schematic of COPS and boundary segmentation workflow is presented in Figure 1 that consists of the following steps:

#### Binning of reads

The mapped reads from the aligned test and the ref SAM files (details of the SAM file generation is provided in the Methods section) are processed using SAMtools [38] Pileup (http://samtools.sourceforge.net/) to yield read depth at every nucleotide position. These are binned into windows of size 50 nucleotides (nts) according to their mapped coordinates in the chr. The bin sizes of 50 and 60 nts are benchmarked using simulated data for all read lengths where the 50 nts bin size yielded better results using simulated data (Table S1).

#### Calculation of pair-wise log_2_ratios

The binned read depths for the test and the ref samples are further processed to calculate test-to-ref log_2_ratios. A stretch of negative log_2_ratio values is typically representative of copy number deletion, while a positive trend denotes a copy number amplification. The bins for which the read depth was zero in either the test or the ref samples or in both was marked as Udef-Deletion, Udef-Amplification and Udef-Neutral events respectively, where the tag ‘Udef’ denotes ‘Undefined’. If an amplification or a deletion event was neighboring to a similar event with a Udef tag, the log_2_ratio for that Udef event was taken to be the same value as the neighboring similar event.

#### Smoothing of read depth

The log_2_ratios were further averaged over every four consecutive bins. For each bin *b*:
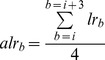

*alr* is the average log_2_ratio, *lr* is the log_2_ratio and *i* is the bin index. This step smoothens the data, by filtering out noise and improves the signal-to-noise ratio. The threshold of 4 for sliding average proved to be the most accurate for CNA detection, as tested with simulated data (Table S1). This sliding average method of smoothing data (rectangular or un-weighted sliding-average smoothing) is the simplest form of smoothing and in our case provided with the best results (Table S1). The log_2_ratios of ten contiguous bins are summed up starting from every bin, in order to provide a cumulative log_2_ratio score (*clr*) for the merged bin.

#### P-value based merging

The log_2_ratios of ten contiguous bins are summed up in order to provide a cumulative log_2_ratio score (*clr*) for the merged bin. We observe the read depths for the test and ref samples to be Poisson distributed (Figure S1), in agreement with earlier findings using read counts ([39] [33], [40]. We also observe the log_2_ratio between read depths of test and ref samples, and the absolute value of the corresponding *clr* to follow a Poissonian distribution (Figure S1). We consider only the magnitude of *clr* while calculating the test statistic and ignore its sign, since we use the statistical framework only to merge bins with a significant *clr*. We observe the *clr* to be Poisson distributed, and its square root to be approximately normally distributed with variance of about ¼, per sample.

Accordingly, the normal [0,1] statistic to assess significance of *clr* for the merged bin is therefore calculated assuming its square root to be approximately normally distributed with a small variance of about ¼, per sample, as follows:
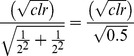



The merged bins are retained for significant *clr*s (P<0.001) as assessed by a normal [0,1] distribution. Contiguous merged bins are further fused into single CNA events. The maximum resolution of COPS is therefore 500 nt, i.e. it can detect CNAs as small as 500 nt long.

#### SCNA boundary segmentation module

The detection of SCNA boundaries is an independent module provided along with COPS, the result of which is fed into COPS to result more accurate SCNA boundaries. This module classifies the anomalous reads from the aligned test and the ref (SAM) files into deletion and amplification categories, mapping with greater and lower than expected insert sizes, respectively. Details on this module are provided in the Methods section.

#### Correction of COPS detected SCNA boundaries

Once the segmentation module detects the boundaries, the COPS SCNA boundaries are further corrected using the results obtained with the added module.

### Pre-requisites, Installation, and Execution of COPS

Pre-requisites:

Operating System: Linux 64 bit.

RAM: 4 GB.

Samtools-0.1.12a or advanced versions.

R programming language version 2.12.1.

Perl module: Distribution.pm.

#### Installation

Decompress the COPS version1.1.zip file to a suitable location. Avoid placing any other files into the extracted folder.

#### Execution

Locate the following files within the Scripts subdirectory, List_test.name & List_ref.name. These files should have all the name of chromosomes in your input sam/bam files (one per line) as per the third field of your input sam/bam file.

E.g. chr1, c1.fa, chr1.fa, c1.

Only the chromosomes specified in the above files will be processed.

Ensure that both files have the same chromosome names and the same number of chromosomes, in the same order. Once the files have been populated appropriately, the main script can be executed as follows.

% bash COPS.sh <input file-type> <test file-name> <ref file-name>

input file-type: 0 for “.bam” file and 1 for “.sam”

test file-name: File name of test/cancer sample (with full path)

ref file-name: File name of reference/normal sample (with full path)

The arguments must be provided in the same order. One must avoid processing multiple sample pairs simultaneously within the same folder. Upon successful execution, an output folder/COPS_output is generated within the/COPSversion1.1 directory. This folder contains the detected SCNA files (Con.*). The final output file carries the following columns: Chromosome name, SCNA start, SCNA end, cumulative log_2_ratio, t-statistic and P-value. The provided sample data contains test.bam and ref.bam.

### Performance of COPS using Simulated Data

**Figure 2:**
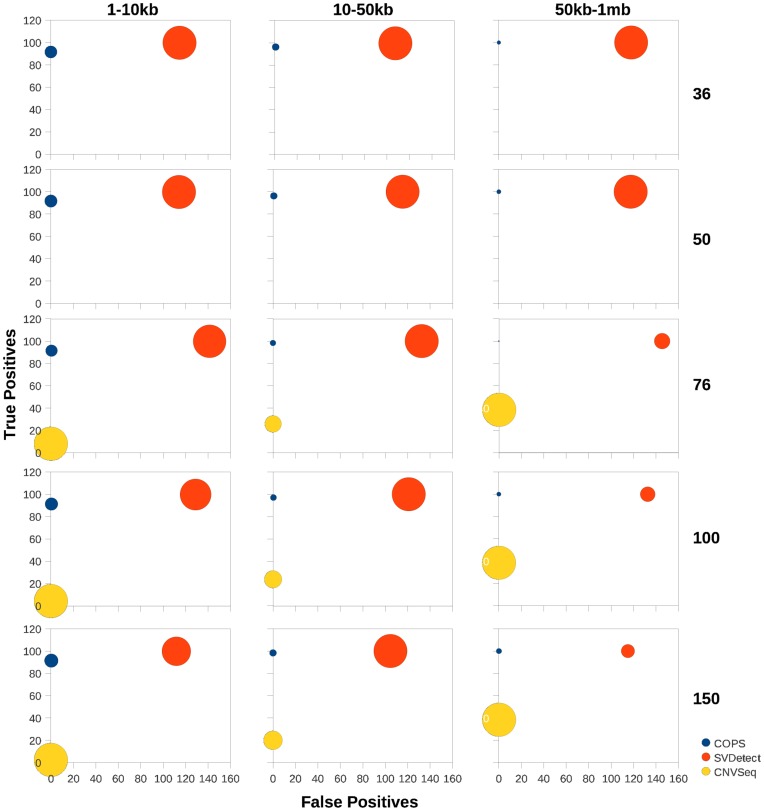
Performance comparison across SCNA detection tools. The percentage of true positive SCNAs (y axes) are plotted against the percentage of false positive SCNAs (x axes) for available SCNA detection tools including COPS. Simulated CNVs at three size ranges: 1–10 kb (A), 10–50 kb (B) and 50 kb–1 mb (C) were used. Paired-end reads of lengths 36, 50, 76, 100 and 150, were generated for each dataset. The size of the data points is representative of the deviation in size of the detected SCNA.

We simulated CNAs in the hg18 ref chr1 sequence to create a test sequence with cumulative size of CNAs equal to 1.5% of the size of chr1. The CNAs were simulated at various sizes (1–10 kb, 10–50 kb, 50 kb–1 mb). We ran available pair-wise CNA detection tools, CNV-Seq [33] and SVDetect [34], along with COPS with all combinations of simulated test:ref paired samples. As depicted in Figure 2, COPS largely outperformed CNV-Seq and SVDetect in terms of sensitivity, specificity and accuracy of CNA size. CNV-Seq is implemented for longer reads (Sanger and 454 derived reads of minimum 250 bp) [33] and did not function for smaller read lengths of 36 and 50 in our comparison study. We used CNV-Seq for read lengths 76, 100 and 150 where it provided only ∼25% of true positive CNAs (Figure 2). Among the small number of calls that CNV-Seq made, most were true positive calls. SVDetect [34], unlike CNV-Seq [33], detected more numbers of SCNAs but with larger fraction of false positive calls (Figure 2). Among the true positive SCNA calls that SVDetect made, the boundaries of SCNAs were far from the true break points, giving rise to more size-deviant SCNAs. The sensitivity of CNA detection goes up with increasing SCNA sizes for all tools (Figure 2). The specificity of CNA detection did not show any such trend. In the case of SVDetect, the specificity dropped to an all time low for read length of 76. Furthermore, the size deviation decreased with increasing SCNA size for COPS, and also for SVDetect for read lengths 76, 100 and 150. However, for CNV-Seq the minimum size deviation is observed for detecting SCNAs in the 10–50 kb size range.

**Figure 3:**
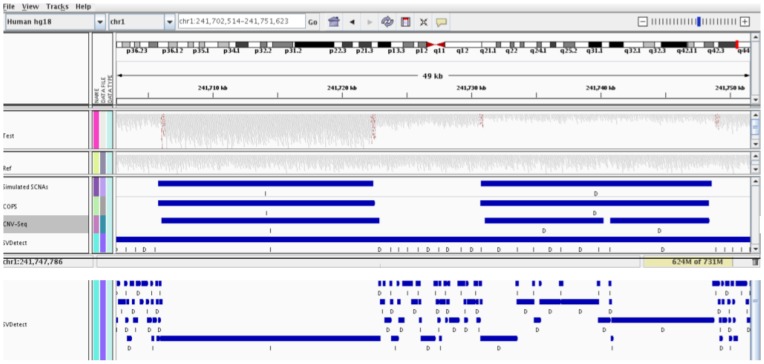
IGV snapshot of performance comparison across SCNA detection tools in the region of chr1.

We then focused on a few regions where there was discordance among the tools in calling SCNAs, with an aim to understand the reason underlying this discordance. In a region of chr1, where COPS correctly detected amplification and deletion, CNV-Seq detected the amplification intact, but not the deletion. Instead, it detected the deleted region as two distinct SCNAs, hence giving rise to a fragmented SCNA (Figure 3). Like CNV-Seq, SVDetect detected the amplification intact but not the deletion. However, the deleted SCNA was further fragmented into multiple calls (Figure 3). SVDetect also detected several false positive SCNAs around the amplification and deletion.

Finally, we tested the tools on a single desktop computer to profile the time taken to complete the CNA detection. For paired-sample analyses, CNV-Seq gave the best time profile (17′04′′), followed closely by SVDetect (22′18′′) and COPS (31′30′′).

In addition to simulations covering 1.5% of chr1, we also evaluated the performance for various SCNA detection tools for simulated SCNAs covering 0.05%, 0.1%, and 3% of chr1 at various read lengths. Like our earlier observation with SCNAs simulated to cover 1.5% of chr1, COPS outperformed CNV-Seq and SVDetect for all SCNA sizes and applicable read lengths when 0.05%, 0.1% and 3% of chr1 simulated SCNAs (Figure S2). Like the CNA detection tools, the CNV detection tools with individual samples and then employing a subtractive approach also corroborated the earlier results with SCNAs covering 1.5% of chr1 (Figure S3).

**Figure 4:**
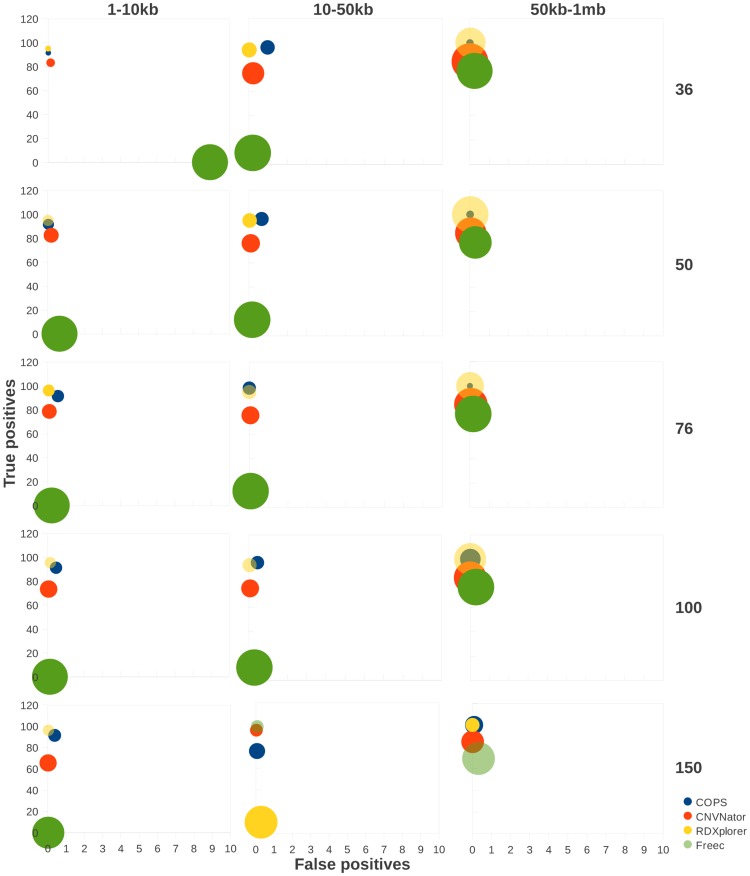
Performance comparison across CNV detection tools. The percentage of true positive SCNAs (y axes) detected using the subtractive approach, are plotted against the percentage of false positive SCNAs (x axes) for available CNV detection tools including COPS. Simulated CNVs at three size ranges: 1–10 kb (A), 10–50 kb (B) and 50 kb–1 mb (C) were used. Paired-end reads of lengths 36, 50, 76, 100 and 150, were generated for each dataset. The size of the data points is representative of the deviation in size of the detected SCNA.

Since the number of SCNA detection tools is limited, we explored the possibility of using individual sample-based CNV callers to detect CNVs in both test and ref sample separately and then employ a subtractive method to detect SCNAs in test sample (details of the subtractive approach is provided in the Methods section). We used the popular CNV calling tools CNVNator [35], RDXplorer [29] and Freec [36] to detect CNVs first and then detect SCNAs by employing the above approach (Figure 4). We used the same set of simulated data described above for these subtractive analyses. COPS and RDXplorer performed best among all tools in detecting SCNAs even when compared with the results from the subtractive approach with CNV detection tools (Figure 4). The size accuracy of COPS was markedly better than that of RDXplorer for the 50 kb–1 mb size range of the simulated SCNAs. CNVNator ranked next in performance comparison. The true positive detection capability of CNVNator did not vary across the SCNA size ranges. The true positive calls made by Freec at all read lengths tested were low except for the 50 kb–1 mb size range of SCNAs (Figure 4). Freec, like SVDetect, detected CNAs larger than the actual size, hence giving rise to more size-deviant SCNAs.

**Figure 5:**
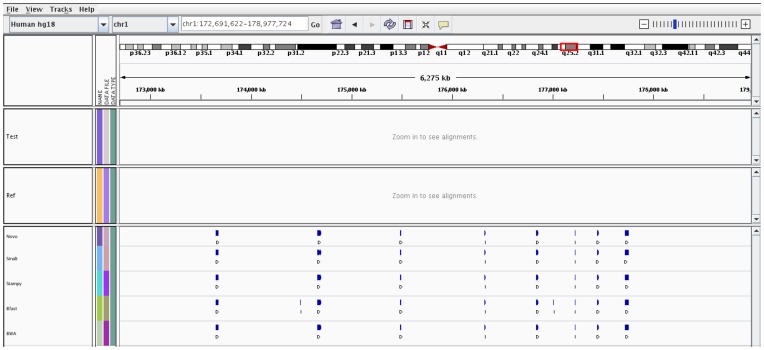
IGV snapshot of performance comparison across CNV detection tools in the region of chr1.

In the same region of chr1 described above, where COPS correctly detected the amplification and deletion in comparison to CNV-Seq and SVDetect, RDXplorer also picked up the amplification and deletion in the test sample CNV detection analyses (Figure 5). CNVNator detected the deletion alone and not the amplification event and Freec detected the amplification alone and not the deletion event.

When tested for the time profiling, CNVNator, Freec and RDXplorer, took 22′18′′, 20′12′′ and 46′ respectively, to complete the CNV detection in individual samples.

**Figure 6:**
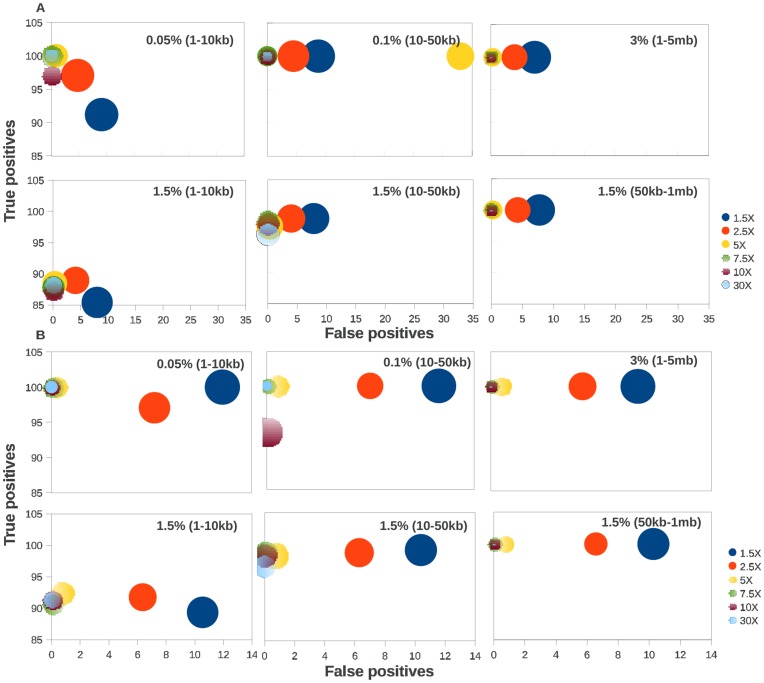
Performance comparisons of COPS at different sequencing read coverage. The performance of COPS using two bin sizes, 50 nt (A) and 60 nt (B), on 76 nt reads,against test:ref pairs simulating SCNAs covering 1.5% of chr1 in the 1–10 kb, 10–50 kb and 50 kb–1 mb size ranges is plotted as true positive calls (y axes), false positives (x axes) and size deviation (size of the data point).

For all the data simulating SCNAs in chr1 (with different coverage and size), we generated paired-end 76 nts long reads with varying coverage, starting from 1.5X up to 30X, and tested the performance of COPS under these conditions at two bin sizes, 50 and 60 nts (Figure 6A & &B respectively). At bin size 50, the minimum coverage at which COPS performed best was 5X with minimum number of false positives (high specificity) and maximum number of true positives (high sensitivity) for most SCNA size ranges except 0.1% (10–50 kb) range (Figure 6A). We found that the results for 5X coverage data at 0.1% CNV (10–50 kb) are reproducible, although the reasons for a discontinuous performance (Figure 6A) are unclear. The minimum coverage extended to 7.5X, when we considered the size deviation aspect as well (Figure 6A). We found no significant difference in sensitivity by increasing the coverage beyond 5X. For read coverage of 2.5X, the size deviation increased further crossing 24% along with concomitant compromise in the sensitivity and specificity of the detected SCNAs. Upon increasing the bin size to 60 nts, we observed a marginal enhancement in sensitivity of COPS for the lowest size range of SCNAs, 0.05% (1–10 kb), but not in specificity or accuracy of size (Figure 6B). COPS did not result in enhanced performance by using other size ranges of simulated CNAs and upon increasing the bin size to 60 nts, including the data using reads generated at low coverage (< = 10X; Figure 6B), in disagreement with the observations highlighted for CNVNator [35].

### Effect of Alignment on SCNA Detection

**Figure 7:**
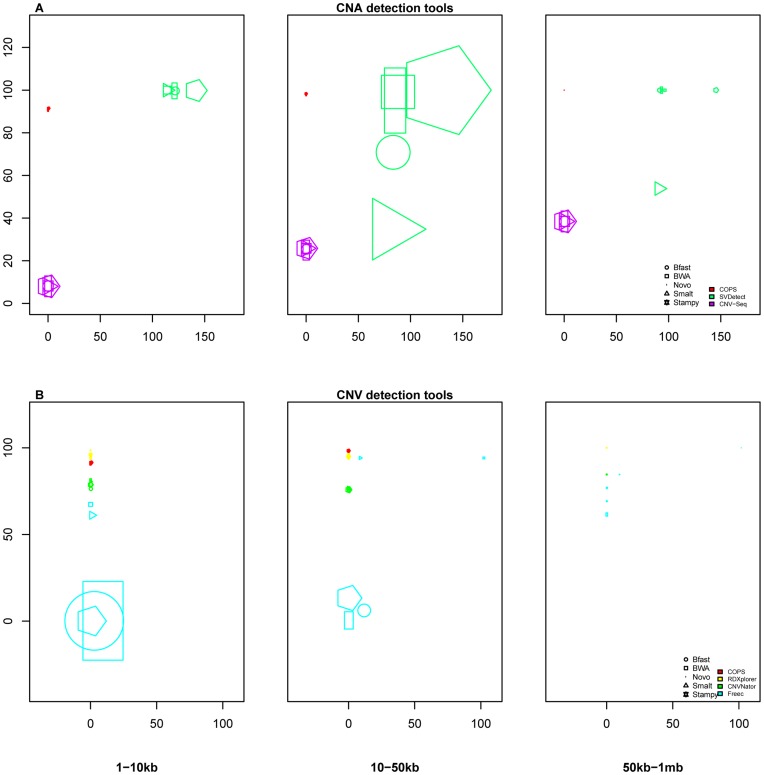
Performance comparisons across short read aligners. Shown are the performances of SCNA (A) and CNV (B) detection tools using 76 nt reads againsttest:ref pairs simulating 1–10 kb, 10–50 kb and 50 kb–1 mb long SCNAs covering 1.50% of chr1, using five aligners. Each plot graphs the false positives (x axes) against the true positives (y axes). The size of the data point indicates the deviation in size of the detected SCNA from the simulated SCNA, the shape indicates the upstream aligner (Text S2) and the color indicates the SCNA/CNV detection tool used in the respective analyses.

In order to understand if the process of read alignment plays a role in detecting CNAs, we aligned reads, generated at different lengths with various coverages of chr1 and sizes, using various open source/freely available aligners like Bfast [41], BWA [38], Novoalign [42] (freely available non-MPI version), Smalt [43] and Stampy [44]. Subsequently, we used the aligned files to call CNAs with all tools including COPS. We first visualized the performance of COPS in comparison to other CNA (Figure 7A) and CNV (Figure 7B) callers. For size ranges of 1–10 kb, 10–50 kb and 50 kb–1 mb, COPS did not show any aligner-dependence on its sensitivity, specificity or size-deviation of SCNA detection. CNV-Seq also did not show any aligner-dependence in sensitivity and specificity of SCNA detection, but did in size-deviation. SVDetect was the most aligner-dependent tool that resulted in lowered sensitivity and elevated size deviation with Smalt (for >10 kb SCNA size ranges), and lowered specificity with Stampy (for most size ranges). Among the CNV detection tools, RDXplorer and CNVNator showed marginal aligner-dependence in the <10 kb SCNA detection sensitivity. Freec showed the highest aligner-dependence: poorer sensitivity and size deviation for Novo, Bfast and Stampy (in that order, for most size ranges) and poorer specificity for Bfast (for >10 kb size ranges).

We subsequently extended our aligner comparison study and observed that our conclusions on aligner-dependent performance of SCNA/CNV detection tools held true for combinations of all other simulated SCNA sizes and read lengths (Figure S4).

**Figure 8:**
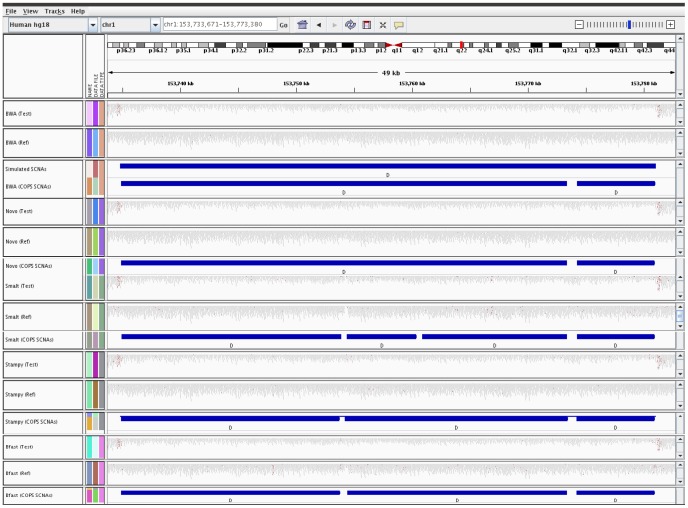
The IGV snapshot of various short read aligners and its effect on SCNA detection. 10–50 kb long SCNAs covering 1.5% of chr1 was used.

We then focused on a chromosomal region encompassing a simulated sample-specific deletion detected as fragments by COPS using all aligners (Figure 8). The fragmentation was minimal for aligners like Novoalign and BWA (2 fragments), but excessive for others like Smalt (4 fragments), and intermediate for aligners like Bfast and Stampy (3 fragments). Incorrect mapping of reads also led to the inaccuracy in SCNA breakpoints (Figure S5) and detection of false positive events, such as the two amplifications detected using COPS with Bfast-aligned reads (Figure S6).

### Performance of COPS with Real Tumor:normal Paired Samples and Validation of SCNAs with Whole Genome SNP Microarray

**Figure 9:**
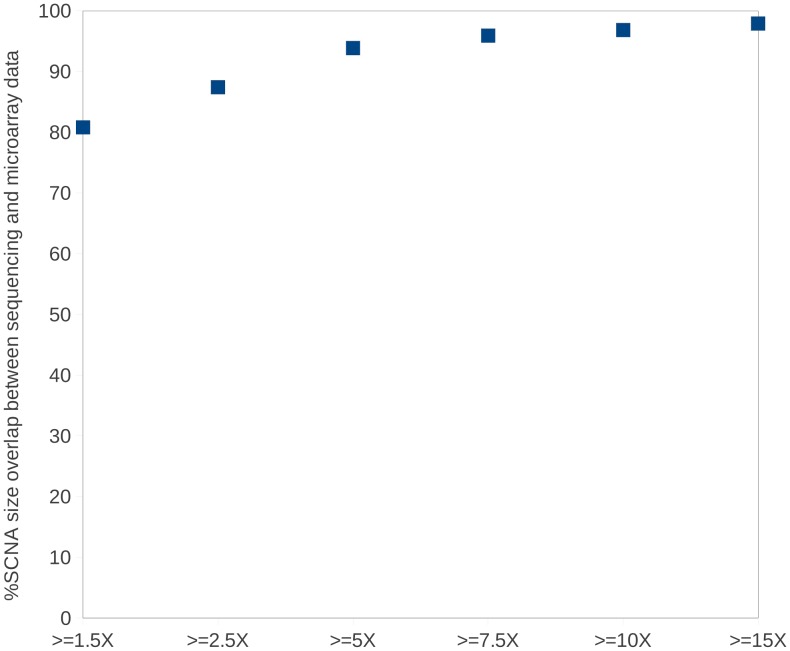
Validation of SCNAs detected using sequencing reads with whole genome SNP microarray. SCNAs detected by COPS using reads from tumor:normal paired samples were overlapped with SCNAs detected by cnvPartition2.4.4 Illumina plugin in GenomeStudio with Omni 2.5 whole genome SNP microarray.

**Figure 10:**
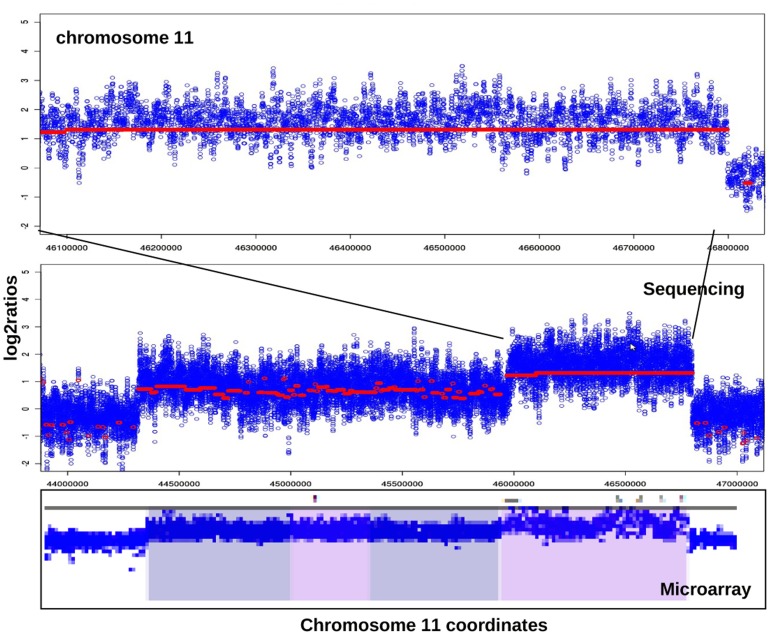
Overlapping of SCNAs detected using COPS and cnvPartition2.4.4 in a region of chr11. The log_2_ratios estimated by COPS using paired tumor:normal reads are plotted before (blue dots) and after (red dots) applying the smoothening function (see zoomed inset), for a section of chromosome 11 harboring SCNAs. A snap-shot of this chromosome section from GenomeStudio is juxtaposed to reveal SCNAs (two major amplifications with different copy numbers) in the same region.

We further tested COPS on real tumor:normal paired samples. We performed whole genome sequencing experiment (5 kb long-insert mate pair library using Illumina Solexa instrument) for a tumor and its matched normal sample and used the data to test COPS in finding SCNAs. We validated the CNAs found by COPS by performing the whole genome DNA microarray (Illumina Omni 2.5 million SNP microarray) on the same sample pair. In order to compare the sequencing data with that from the microarray, we took only those regions with reads where there were at least 5 probes tiled on the microarray. The concordance between CNAs obtained using COPS and DNA microarray was 80% when regions with > = 1.5X coverage were considered that increased considerably to 97.9% with regions with > = 15X coverage (Figure 9 and Table S2) consistent with our finding in simulated data (Figure 6). We visualized a region of chromosome 11 harboring major amplifications by juxtaposing a screen-shot from Illumina GenomeStudio loaded with the real tumor:normal B allele frequency (BAF) ratios and the CNV Analysis bookmarks, against a plot of log_2_ratios from COPS using sequencing reads validated contiguous regions of amplification type SCNA events within the chromosome 11 (Figure 10). The boundaries of the 1 and 1.5 copy amplification events, detected independently across different platforms, coincided perfectly thus further validating the performance of COPS.

### Identification of Precise SCNA Boundaries

In order to detect precise SCNA boundaries using short reads; we used a module as outlined in Figure 1. This is an independent module, the results of which are fed into COPS to fine tune the SCNA boundaries. We used the difference in density of anomalous reads near COPS detected CNA start and end boundaries as a measure to improve their precision. Anomalous reads, defined as paired-reads mapped with deviant insert sizes, result due to structural variations, mainly copy number variations and translocations. An amplification or deletion event results in anomalously mapped paired-reads with a lesser or greater than expected insert size respectively. Details on the binning of anomalous reads, calculating ratios between binned anomalous read counts of neighboring bins, and detecting copy number aberration boundaries are provided in Methods section. The CNV boundaries detected in the test (tumor) sample are compared with those detected in the ref (normal) sample and filtered using the subtractive approach in order to arrive at precise boundaries. In order to detect sensitivity and specificity of boundary detection using the module, we have compared the boundaries resulting from both the sequencing reads with that from the Illumina whole genome SNP genotyping microarray that use the CNVPartition algorithm [45] to detect boundaries. COPS detected 66.1% of the boundaries found by the whole genome SNP microarray. For individual breakpoints, we analyzed a region of chromosome 11 with COPS detected SCNAs validated with DNA microarray and found that the boundaries detected with sequencing reads match with the ones detected by the microarray (Figure S7).

In order to understand the discordance between sequencing and microarray-derived boundaries, we explored the presence/absence of sequencing reads and presence/absence of probes in the whole genome SNP microarray. SCNA breakpoints found using microarray and not with sequencing reads were due to the presence of lower read coverage. The average and maximum depth of coverage per nt per CNA in the region of concordance between microarray and sequencing were 0.55 and 0.84 respectively and in the region of discordance were 0.3 and 0.5 respectively. We then investigated the SCNA boundaries detected using sequencing reads but not with the microarray and found that 99.91% of the discordant breakpoints were due to the absence of probes in the microarray that correspond to the region of SCNA detected using sequencing reads.

## Discussion

Structural variations play an important role in diseases like cancer. Despite the presence of many tools described earlier in detecting copy number variations, there is a need for studies in detecting acquired structural variants in tumor (or diseased) samples using improved methods of detection. This report describes an approach, COPS, to discover SCNAs, i.e. sample-specific regions of copy number alterations between paired (normal and tumor) samples. We used trial data and comparisons to other methods to judge the performance of COPS. COPS fine-tunes common, if not universal, approaches (binning, ratios, smoothing, etc.) based on read depth, and incorporates an internal, robust and non-heuristic statistical method to judge the probability that a called CNA is truly deviant. Despite this simplistic approach, the method and results obtained are useful to the biology community where a simple approach to find pair-wise and tumor (disease)-specific copy number alterations is desired that can run on a desktop computer with very little knowledge and know-how on sophisticated bioinformatics tools.

Most CNV detection tools are optimized to perform well for a single sample (and not paired samples) and for a particular size range of amplifications and deletions [35] and since different complementary approaches discover ∼30–60% of CNVs, the results obtained using these tools cannot directly be compared to each other [35]. On the other hand, COPS performed well over a wide size range of SCNAs and for different read lengths. Any sequencing errors and/or experimental anomalies introduced during imaging and/or sequencing library preparation do not account for any possible bias in our analysis as both test and ref samples are equally subjected to those biases, hence giving rise to real tumor (disease)-specific alterations. COPS scales up well in detecting larger SCNAs (>10 kb), in terms of sensitivity, specificity and size deviation. The improved performance of COPS compared to other tools at a higher size range works to its advantage in detecting cancer-specific SCNAs. We tested the performance of COPS repeatedly on simulated and real data, and find the results obtained using COPS to be reproducible for a given dataset, as expected for a non-heuristic approach. Some CNV detection tools like RDXplorer [29] adopts a method of filtering out reads of low mapping quality (<Q30). Such a filter is not necessary in a pair-wise approach like COPS. Another pair-wise CNA detection tool, CNASeg [46], also uses the depth of coverage information to calculate CNAs in tumor samples. However, we could not include CNASeg in our performance comparisons due to lack of availability of a compatible (working) version of the software that works in our computing environment (personal communication with Sergei Ivakhno). The post-processing errors in filtering false positives and merging are lowered when the paired log_2_ratios are significantly different from 0, therefore, making COPS perform well in detecting larger CNAs. CNV-Seq [33] and SVDetect [34] use paired log_2_ratios to calculate CNAs but perform poorly in our comparative study. This is most likely because they lack any pre- or post-processing steps, such as defining undefined log_2_ratios (caused due to lack of reads in either test or ref or both samples) based on their neighboring bins, smoothing of the data, filtering false positives and merging SCNAs.

Additionally, bin size is one of the important factors in determining the accuracy of SCNA identification and varies according to read length, sequencing coverage (Table S1) and data quality [35]. However, since our approach is based on depth of coverage at each nucleotide position, we used a fixed bin size that renders its performance invariant across read lengths. The current tools for CNV detection do not detect all the true positive CNAs across the genome for a wide-range of read lengths. Abyzov et al. [35] discuss the need of alternative approaches for detecting CNVs with sequencing data of larger read lengths. However, we find that COPS scales up in its performance for reads with length upto 150 base pairs for most CNA size ranges, partially corroborating the finding of Abyzov et al [35].

Alignment of raw sequence reads to a reference genome is the first step in NGS data analysis. Read lengths, sequencing errors, repeat regions of the genome and presence of SNPs and/or indels affect the efficiency of alignment of reads to the reference genome. Data from our lab [47] have shown that post-alignment base calibration and not the alignment per se has a huge impact on finding true positive single nucleotide variants from the sequencing data and increases the sensitivity of detection of variants. Although the effect is minimal, it was not surprising that the some of the most sensitive aligners performed better, although marginally over others, when tested with COPS for the detection of CNAs. COPS does not contain any module for correction of GC bias during sequencing. In an approach based on inter-sample ratios, we believe GC correction is not necessary, because the bias within a bin is inherently corrected for during calculation of the ratio. COPS, being a paired ratio-based approach, allows analysis of reads to repeated gene clusters and segmental duplications such as the beta-defensin gene [25]. Tumor heterogeneity is a major issue that may complicate the downstream sequencing analysis with cancer samples. International Cancer Genome Consortium requires researchers to use samples with at least 80% tumor cells on histological assessment and less than 20% necrotic/normal cells [48]. Presently most researchers focusing on cancer genome sequencing use samples with very high degree of tumor cells in their samples. However, in order to cover a wide variety of cancer samples, both sequencing technology and analytical tools need to be developed that can take into account high degree of cellular heterogeneity. COPS is not designed to be used for samples that has high degree of heterogeneity and assumes a very high percentage of tumor cells in samples. Additionally, as COPS relies on a paired approach, it assumes uniform sequencing coverage for both the ref and test samples. In case, the samples are sequenced at different read coverage, the ratio of the coverage can be factored in to accordingly determine what ratio of read-depth can be termed as baseline neutral.

Once we validated SCNAs detected by COPS with high-density whole genome SNP microarray using real tumor:normal sample pair (Figure 9), we wanted to test the impact of read coverage on the sensitivity of SNCA detection. We found that the required resolution in binned read depths to call pair-wise CNAs dropped for reads with coverage < = 5X, particularly when the binned read depths for one or both the samples was too low. This was confirmed by our observation of lower concordance between CNAs detected using COPS on low coverage (<5X) tumor:normal complete genome sequencing data, and subtractive CNVs detected for the same samples using the whole genome SNP microarrays (Figure 9). By increasing the threshold further in the CNA regions detected in the microarray data (by filtering out the low coverage bins with read depths of < = 7.5X), the concordance of finding CNAs between sequencing and array data increased to 95.9%, validating the dynamic range drop off at ∼7.5X with the simulated data (Figure 6). SCNAs detected across individual chromosomes also indicates a dynamic range drop off of ∼7.5X for a majority of the chromosomes (Table S2).

Boundary mapping is an important step for any CNA detection. There are reports that use soft-clipped reads to detect breakpoints [49]. However, soft-clipped read mapping gave rise to a higher percentage of false positive breakpoints in our sample. Instead, the approach of using anomalous read mapping and difference in density between anomalous reads proved to be a better approach in detecting precise boundaries. This is demonstrated by the higher percent of breakpoint concordance between SNP microarrays and sequencing reads. The exact boundaries of CNVs, hence exact breakpoints, depend on the upstream aligner used to map short sequencing reads to the reference genome. Introducing boundary correction based on differential densities in anomalous reads is aligner-dependent as different aligners use different parameters to map anomalous reads. COPS (when used without the boundary segmentation module) reported only those SCNAs, which fall within a 10% margin of variability in the CNA breakpoints, as found in simulated data. The tagging of simulated SCNA boundaries with anomalously paired reads was best demonstrated with the aligner Novoalign. This is not surprising given that Novoalign is one of the most sensitive aligners known [47]. The breakpoint estimation algorithm used by Illumina’s plugin cnvPartition uses a systematic sliding window approach over 4, 8, 16 and 32 probes to detect consistent departure in preliminarily inferred copy number states from the neutral copy number state of 2, and thus identify maximally different segments [45]. Breakpoints are then called at the boundaries of these maximally different segments and visualized by the Illumina software GenomeStudio. We found that most of the breakpoints that are found in arrays and not in the sequencing-based approach were due to the lack of reads in the sequencing data and those found with sequencing reads but not with the array-based approach were due to lack of any probes for those regions in the array. Unlike COPS, the boundary segmentation module relies on anomalous read mapping in individual samples, and hence, does not require equal read coverage of the test and the ref samples.

### Conclusion

We have developed a pair-wise, easy to use, biologist-friendly, somatic copy number alteration (SCNA) detection tool, COPS, for short-read NGS data, specifically designed to identifying somatic CNAs in cancer/disease samples over a wide-range of read lengths. Also, we reported an independent boundary segmentation module, the results from which can be fed into COPS to fine tune the SCNA boundaries. Although COPS is not designed to detect CNVs between two different individuals but between paired samples from the same individual, its ability to find subtractive copy number alterations allows it to be applicable to different individuals, sequenced under the same conditions. Ratio-based approaches, using paired-sample approach, have been used in the past for CNA detection using sequencing and microarray data. We found that the challenge in discovering true CNAs using sequencing data (short-insert single/paired-end or long-insert mate pair) primarily lies in the choice of taking read depth and not read count along with the processing of data prior to and post calculation of ratios such as choosing the correct bin size, filtering background noise, merging bins and filtering false positives. COPS incorporates all these pre- and post-processing steps to allow for a smooth and progressively improving work flow for SCNA detection. By using a database of known CNVs discovered in normal population and other disease/cancer samples, one can find CNAs that might play specific role(s) in disease progression. Although the cost of performing sequencing for longer reads is going down, we have shown that to detect most true positive CNAs in cancer sample, one doesn’t need longer reads but decent coverage. We recognize that the ability to detect all the disease causing CNVs in a sample does not merely depend on the sequencing coverage but also on the ability of a particular technology/chemistry to reproducibly sequence the difficult/low complexity regions of the genome and hence the completeness of sequencing.

## Methods

### Data Simulations, Whole Genome Sequencing of Tumor:normal Paired Sample, Use of Short-read Sequence Alignment

We simulated wide size-range and numbers of SCNAs with a fixed coverage for chr1 of the human reference genome (ref) hg18 (downloaded from UCSC) and used dwgsim [http://sourceforge.net/apps/mediawiki/dnaa/index.php?title=Whole_Genome_Simulation] to generate reads using a downstream read generator. A mean insert size of 250 bp with a standard deviation of 50 was used to generate simulated reads for both sample (test) and the ref samples to form a test:ref pair. The percentages of chr1 used and sizes of CNVs simulated are: 0.05% (1–10 kb), 0.1% (10–50 kb), 1.5% (1–10 kb, 10–50 kb and 50 kb–1 mb) and 3% (1–5 mb) for coverage, 1.5X, 2.5X, 5X, 7.5X, 10X, 15X and 30X (where 1X means reads covering the entire length of chr1) for paired-end reads.

Human samples were obtained after ethics committee approval from Mazumdar Shaw Cancer Centre, Narayana Hrudayalaya, Bangalore, India and after obtaining written informed consent from the participants involved in this. Illumina GAIIx was used to sequence the tumor:normal paired sample for oral tumors following Illumina long-insert (5 kbp) mate pair protocol with 3–5X coverage. Raw sequence reads from the simulated test:ref and tumor:normal pairs were aligned using Novoalign (version 2.07.05), with parameters set to maximize alignment accuracy (http://www.novocraft.com). Novoalign was used with the default option that keeps the best-aligned read at each location. In order to test the effect of alignment on CNA detection, in addition to Novoalign [42], we used other open-source aligners widely used by the sequencing research community, like BWA [38], Bfast [41], Smalt [43] and Stampy [44]. All aligners were used with default options. Aligned data were generated in SAM format, converted to BAM, sorted according to the mapped read coordinate position by using SAMtools (http://samtools.sourceforge.net/) and reconverted to the SAM format before further processing.

### Benchmarking COPS

Various combinations of simulated data, described above, were used to benchmark COPS against other commonly used open source pair-wise CNA detection tools like CNV-Seq [33]and SV Detect [34] and single sample CNV detection tools like CNVNator [35], RDXplorer [29] and Freec [36]. We ran CNVNator, RDXplorer and Freec, being individual sample based CNV detection tools that do not rely on a matched sample, on test and ref samples separately, and then subtracted the CNV outputs of the ref from test in order to give us a pair-wise CNA output comparable to COPS. The output of pair-wise CNA algorithms such as CNV-Seq and SV Detect were used directly for comparison with COPS. All tools were run with default options. Details of commands and configuration files are provided in Text S1.

We used four parameters to benchmark COPS against other tools. They are sensitivity (% of true positives), 100 - specificity (% of false positives), % size-deviation (size of the detected SCNA – size of the simulated SCNA/size of the simulated SCNA×100), and time taken to run each of the tools. In order to translate the % of true and false positives into actual numbers of SCNAs detected and undetected, we have provided the numbers of simulated SCNAs (deletions and amplifications) in Table S3. We ran the time profiler for all tools on a single 3.2 GHz Intel I3 processor with 2TB SATA hard disk, 4GB DDR3 RAM loaded with Linux Ubuntu 10.10 64 bit operating system on the dataset with 1.5% of simulated SCNA in chr1 in 10–50 kb size range.

### Validation of CNAs Found by COPS in Tumor:normal Paired Samples

We performed whole genome SNP microarray experiment with the same tumor:normal paired samples. Genomic DNA was hybridized onto whole-genome OMNI SNP arrays from Illumina following manufacturer’s specifications. Array CNVs from individual samples were found by using the plug-in cnvPartition 2.4.4 present in Illumina Genome Studio software. The arrays had 2.5 million SNPs selected from the whole human genome tiled onto them. CNAs detected with COPS using the tumor:normal paired samples were validated against the CNVs found by whole genome microarray by subtracting (test MINUS ref) CNVs detected for the same sample pair. Concordance was estimated as the fraction of overlapping loci between CNAs detected across the sequencing and array platforms for the sample pairs being studied, out of the total loci involved in CNA events from the two platforms. Only those COPS detected SCNAs were considered for overlap which had at least 5 array probes tiled in that region, and only those array SCNAs were considered for overlap which had the necessary coverage of sequencing reads in both samples, especially while testing coverage thresholds as part of failure analyses for any discordance.

### Identification of Precise SCNA Boundaries

COPS boundary segmentation module takes the anomalous reads from the aligned test and the ref (SAM) files into deletion and amplification categories, mapping with greater and lower than expected insert sizes, respectively. The anomalous reads from both categories were subsequently binned into bins of size 5000 nts. The anomalous read count ratios are then computed between adjacent bins. and SCNA boundary segmentation is determined between adjacent bins, where the ratios were 0±0.1 and 0.5±0.1 for the deletion category and 1.5±0.1, 2±0.1, 2.5±0.1, 3±0.1, … for the amplification category. These ratios correspond to distinct copy number states of 0 (full deletion), 1 (mono-allelic deletion), 3 (mono-allelic amplification), 4 (amplification of both alleles) etc. assuming uniform sequencing coverage across the genome. Once such boundaries were detected in both tumor and normal samples, the boundaries detected in the normal sample are filtered from that of tumor (within ±100 nt) for SCNA boundaries. The results from COPS SCNAs were further corrected using the precise boundaries detected by the additional module.

Details on the scripts and options used during alignment are given in Text S2.

## Supporting Information

Figure S1 **Poissonian fits to read depths, log2ratios and** ***clr.*** Frequency histograms were plotted for log_2_ratios, cumulative log_2_ratios (*clr*) summed over 10 consecutive bins, binned read depths of test and ref samples. Fits to Poisson distribution are further plotted (shown in red) for each histogram.
(PDF)

Figure S2 **Performance comparison across SCNA detection tools.** The percentage of true positive SCNAs (y axes) are plotted against the percentage of false positive SCNAs (x axes) for available SCNA detection tools including COPS, using data simulating SCNAs covering 0.05%, 0.10% and 3% of chr1 at three size ranges, respectively: 1–10 kb (A), 10–50 kb (B) and 1 mb–5 mb (C). Paired-end reads of lengths 36, 50, 76, 100 and 150, were generated for each dataset. The size of the data points is representative of the deviation in size of the detected SCNA.
(PDF)

Figure S3 **Performance comparison across CNV detection tools.** The percentage of true positive SCNAs (y axes) detected using the subtractive approach, are plotted against the percentage of false positive SCNAs (x axes) for available CNV detection tools including COPS, using data simulating SCNAs covering 0.05%, 0.10% and 3% of chr1 at three size ranges, respectively: 1–10 kb (A), 10–50 kb (B) and 1 mb–5 mb (C). Paired-end reads of lengths 36, 50, 76, 100 and 150, were generated for each dataset. The size of the data points is representative of the deviation in size of the detected SCNA.
(PDF)

Figure S4 **Performance comparisons across aligners.** Shown are the performances of six SCNA/CNV detection tools using reads generated at five lengths (36, A; 50, B; 76, C; 100, D; 150, E) against test:ref pairs simulating six SCNA sizes and mapped to the chr1 reference sequence using five aligners. Each plot graphs the false positives (x axes) against the true positives (y axes). The size of the data point indicates the deviation in size of the detected SCNA from the simulated SCNA, the shape indicates the upstream aligner (Text S2).
(PDF)

Figure S5 **Performance comparison of COPS using different aligners.** An IGV snapshot captures variation in SCNA boundaries detected by COPS using reads mapped by different upstream aligners.
(PDF)

Figure S6 **Performance comparison of COPS using different aligners.** An IGV snapshot captures detection of false positive amplification-type SCNA events using COPS with Bfast-aligned reads.
(PDF)

Figure S7 **A differential density of anomalous reads near SCNA boundaries.** The insert sizes of anomalously mapped paired reads below the expected insert size of 5000 nts including a standard deviation of 500 nts are plotted for a region of chromosome 11 harboring two amplification-type SCNAs, individually for the tumor and the normal sample.
(PDF)

Table S1 **Performance of COPS at varying bin sizes for binning read depths and step sizes for smoothening the log_2_ratios.**
(PDF)

Table S2 **Chromosome-wise validation of SCNAs detected by COPS using whole genome SNP microarray.**
(PDF)

Table S3 **Number of SCNAs in each simulated dataset.**
(PDF)

Text S1 **Commands used to run various CNA/CNV detection tools for performance comparison with COPS.**
(PDF)

Text S2 **The R codes and a sample input file used to generate** **Figure 7** **and Figure S3.**
(PDF)
