# Supplementary figures and images for: COPS: A Sensitive and Accurate Tool for Detecting Somatic Copy Number Alterations Using Short-Read Sequence Data from Paired Samples
Source: PLoS One. 2012 Oct 22;7(10):e47812. doi: 10.1371/journal.pone.0047812 (PMC3478291; doi:10.1371/journal.pone.0047812)

Supporting Figure 1

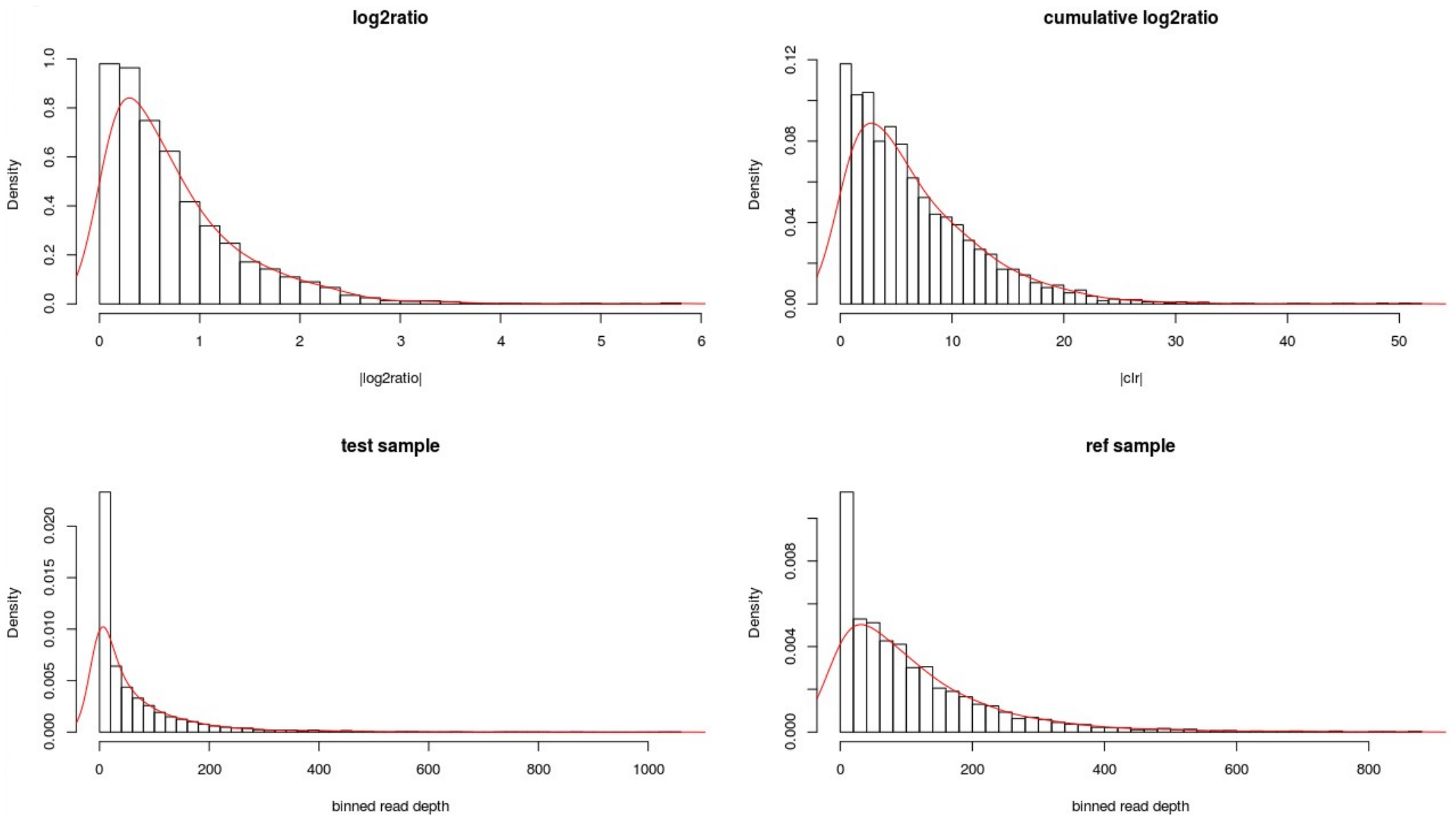

Supplement: Figure S1 — Poissonian fits to read depths, log2ratios and clr. Frequency histograms were plotted for log2ratios, cumulative log2ratios (clr) summed over 10 consecutive bins, binned read depths of test and ref samples. Fits to Poisson distribution are further plotted (shown in red) for each histogram. (PDF) [file pone.0047812.s001.pdf]

0.05% (1-10kb)

0.1% (10-50kb)

3% (1-5mb)

36

50

76

100

150

● COPS  
● SVDetect  
● CNVSeq

True positives

False positives

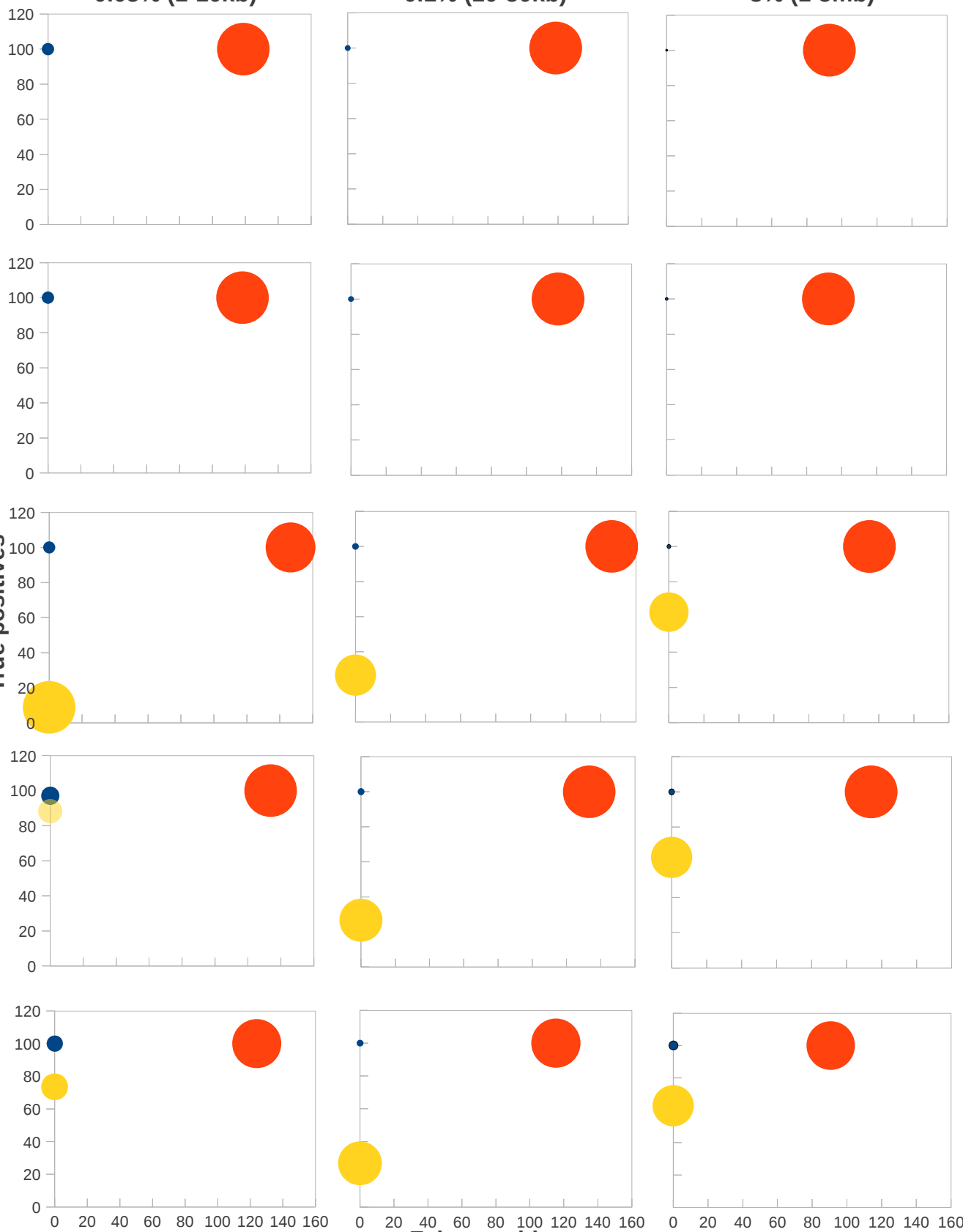

Supplement: Figure S2 — Performance comparison across SCNA detection tools. The percentage of true positive SCNAs (y axes) are plotted against the percentage of false positive SCNAs (x axes) for available SCNA detection tools including COPS, using data simulating SCNAs covering 0.05%, 0.10% and 3% of chr1 at three size ranges, respectively: 1–10 kb (A), 10–50 kb (B) and 1 mb–5 mb (C). Paired-end reads of lengths 36, 50, 76, 100 and 150, were generated for each dataset. The size of the data points is representative of the deviation in size of the detected SCNA. (PDF) [file pone.0047812.s002.pdf]

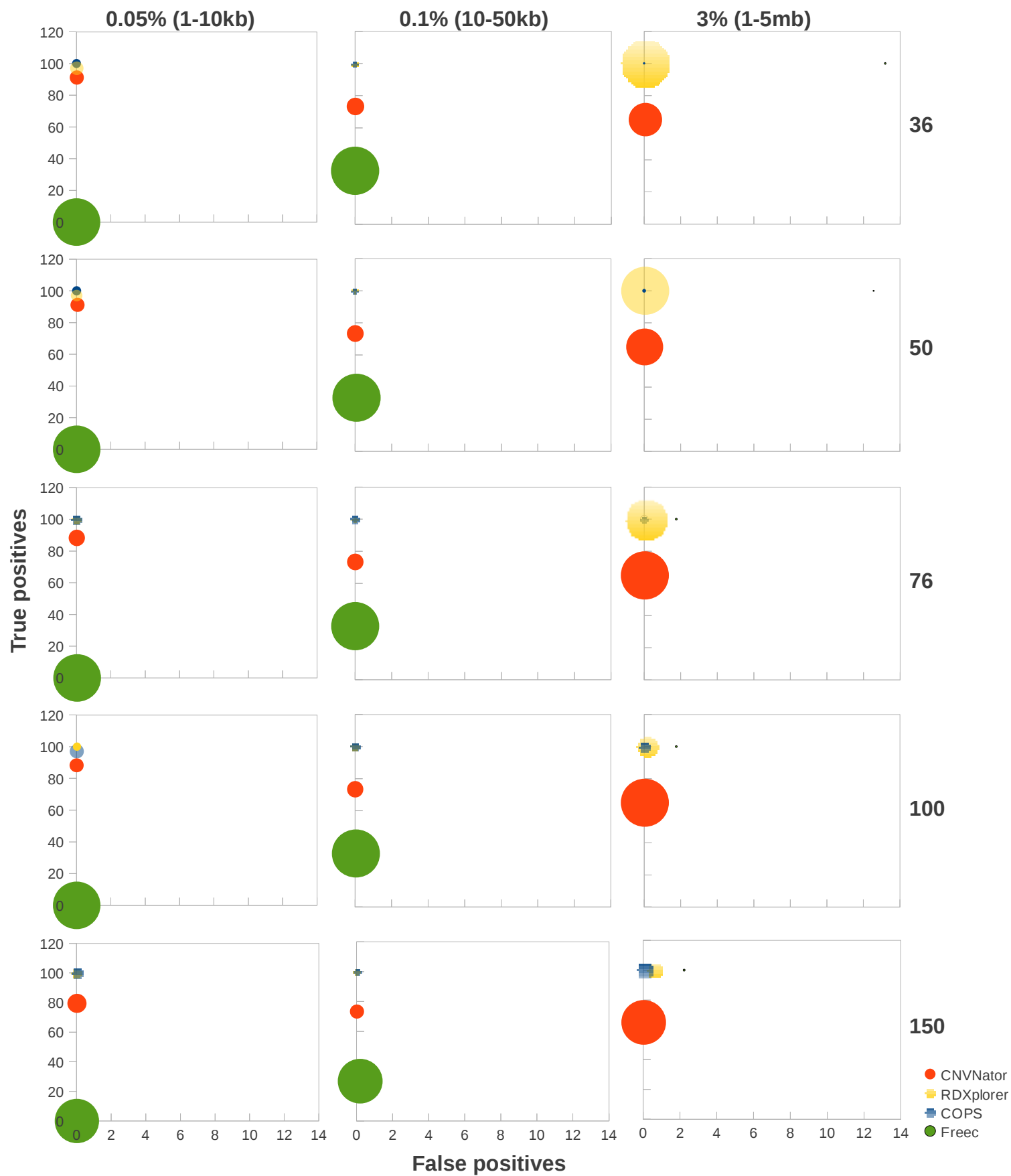

Supplement: Figure S3 — Performance comparison across CNV detection tools. The percentage of true positive SCNAs (y axes) detected using the subtractive approach, are plotted against the percentage of false positive SCNAs (x axes) for available CNV detection tools including COPS, using data simulating SCNAs covering 0.05%, 0.10% and 3% of chr1 at three size ranges, respectively: 1–10 kb (A), 10–50 kb (B) and 1 mb–5 mb (C). Paired-end reads of lengths 36, 50, 76, 100 and 150, were generated for each dataset. The size of the data points is representative of the deviation in size of the detected SCNA. (PDF) [file pone.0047812.s003.pdf]

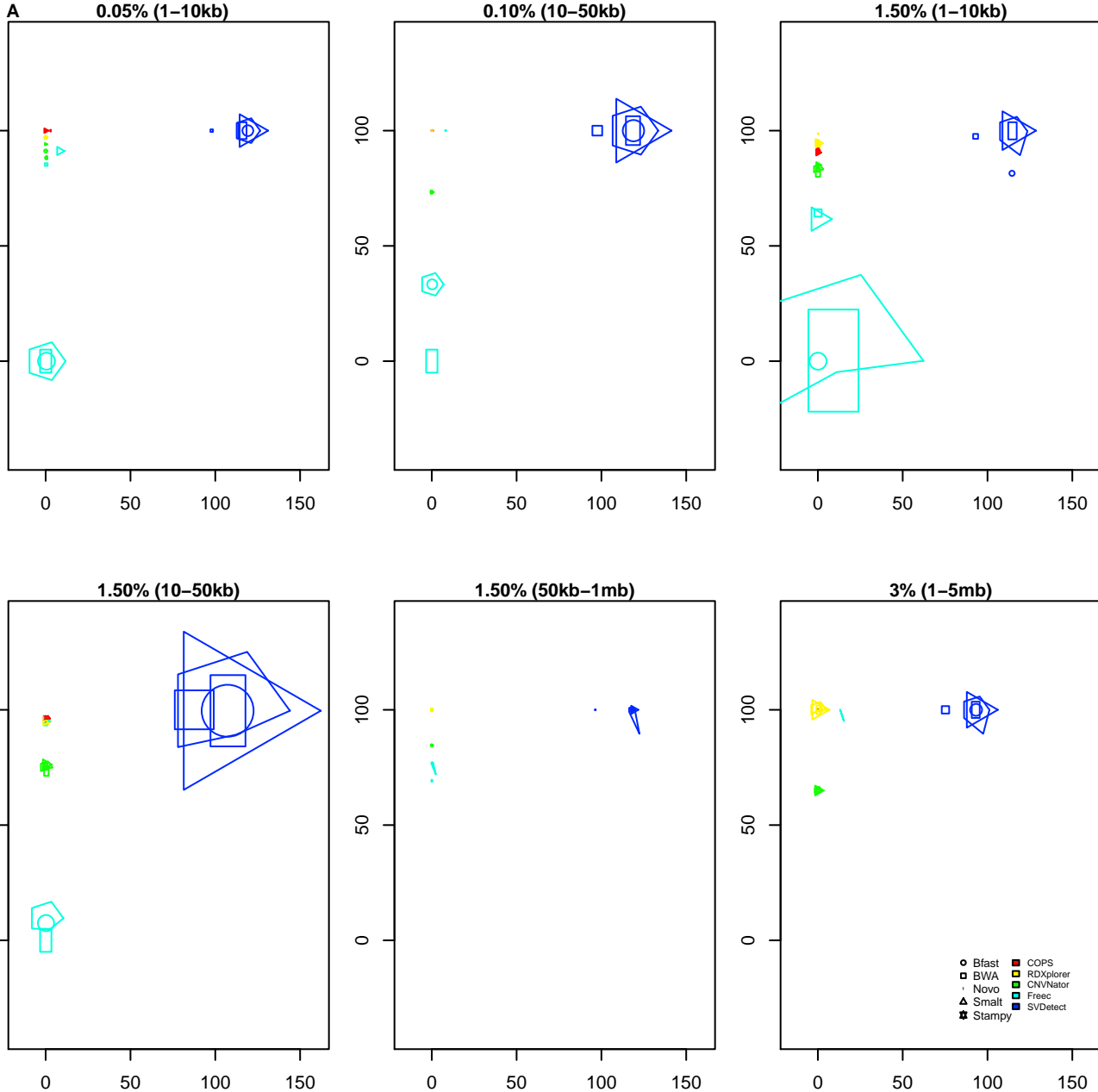

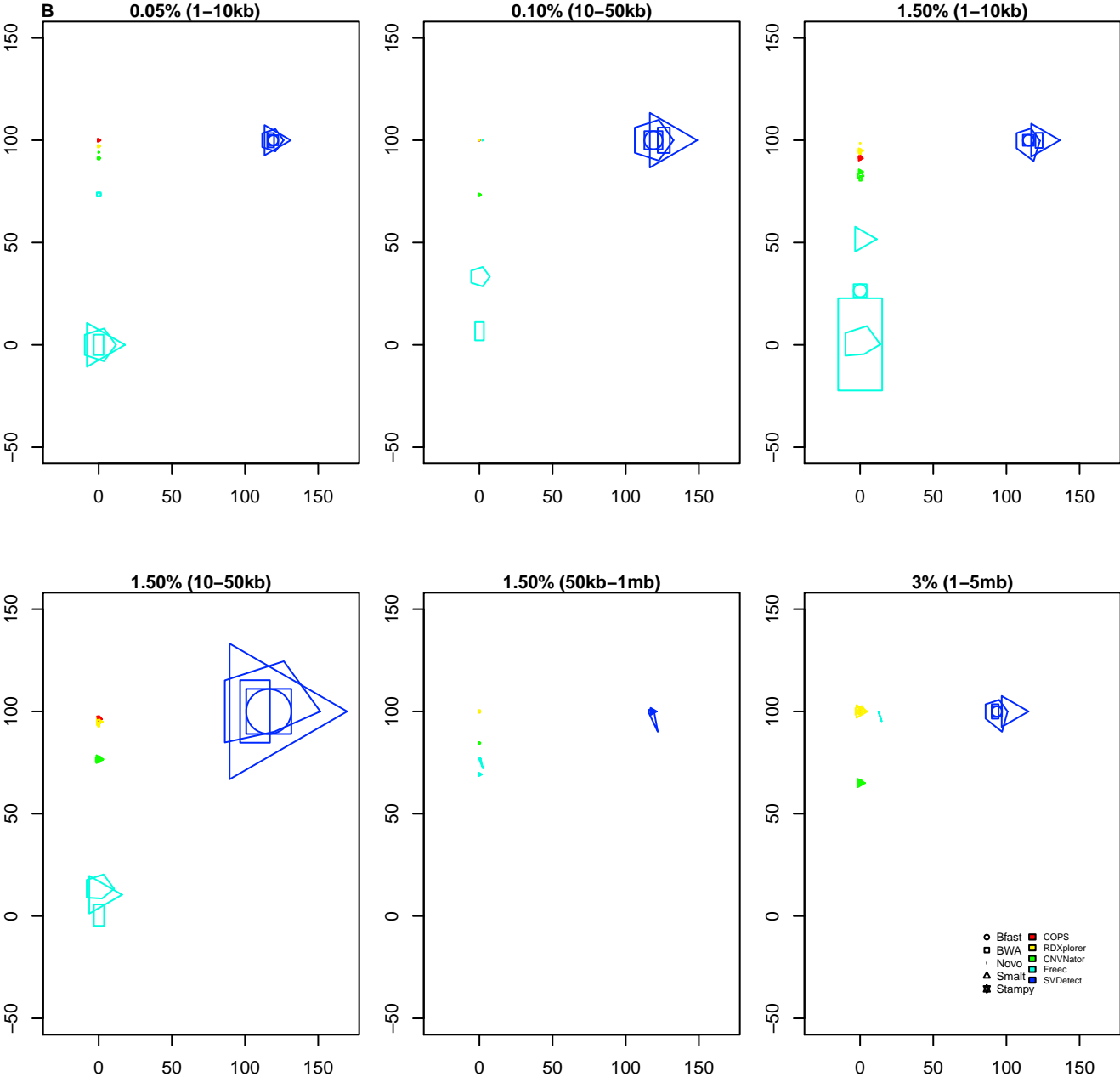

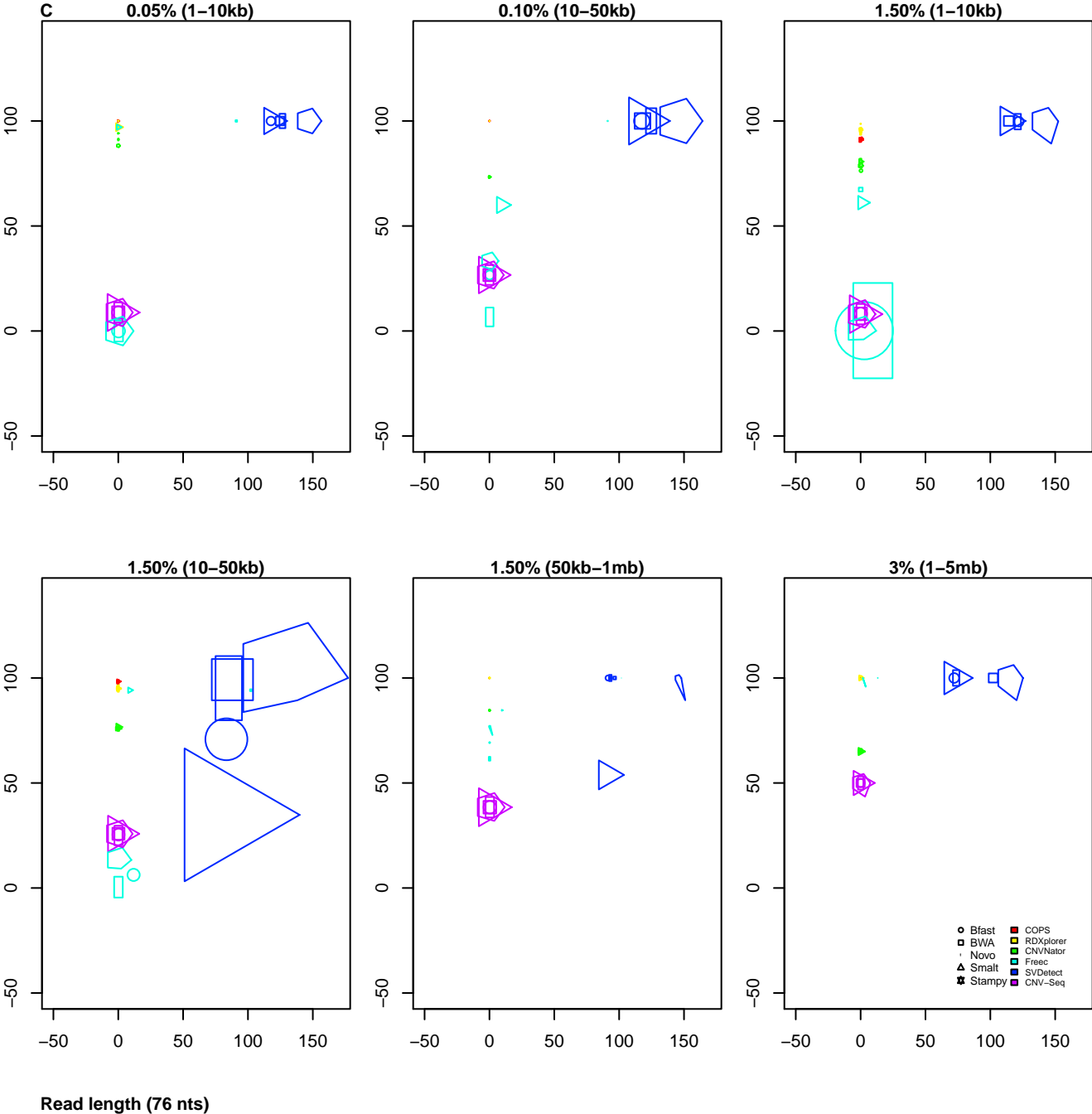

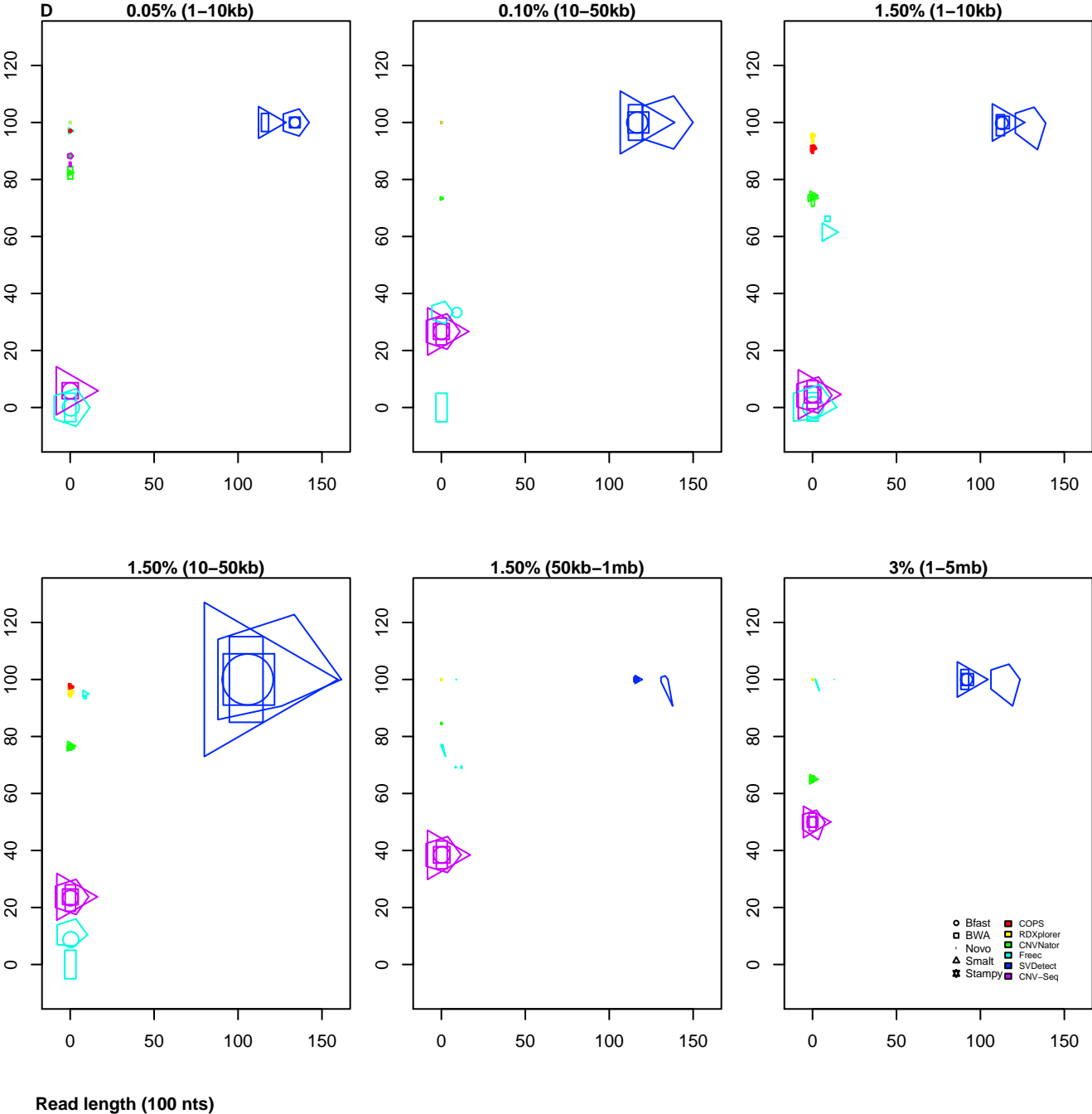

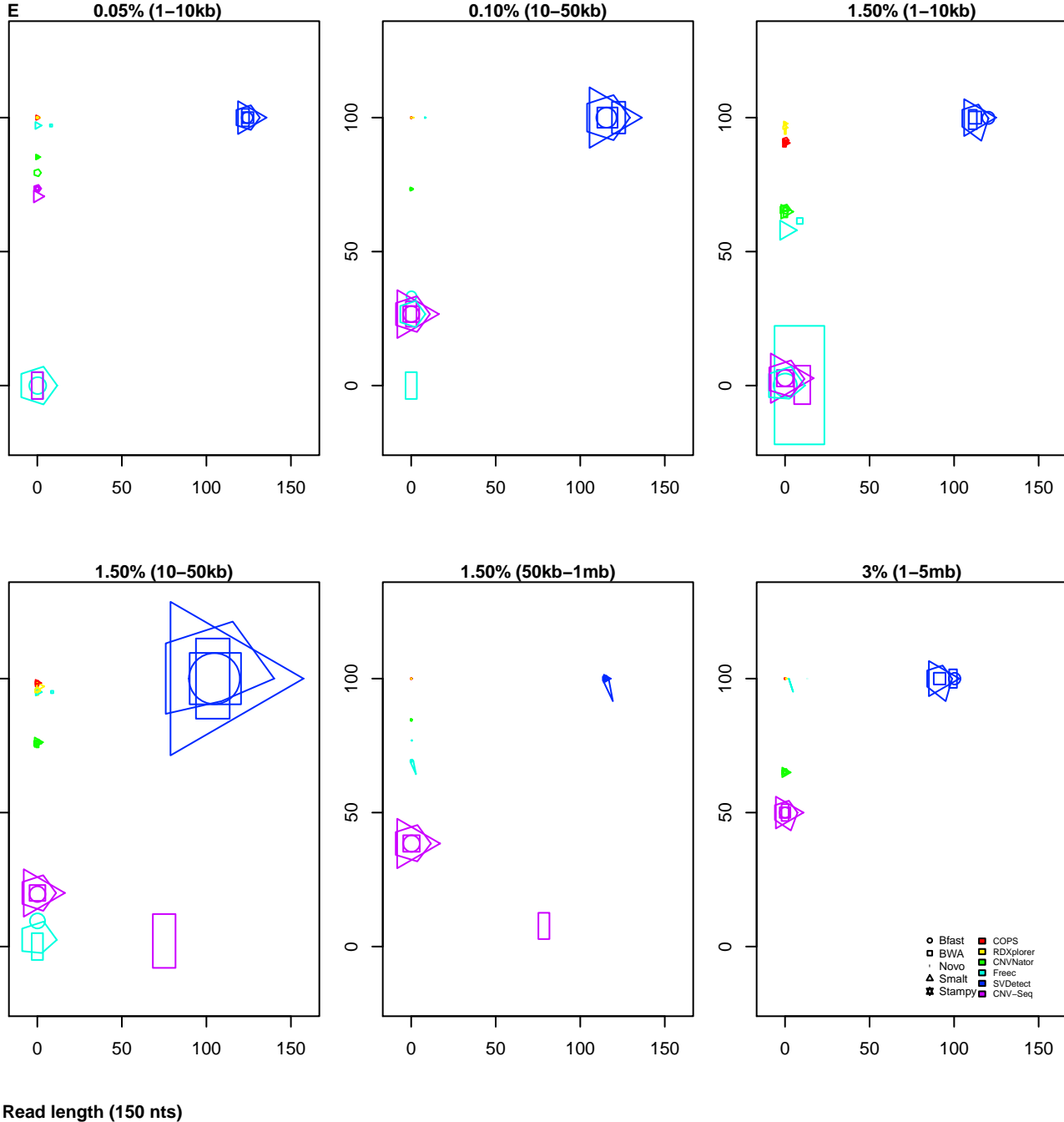

Supplement: Figure S4 — Performance comparisons across aligners. Shown are the performances of six SCNA/CNV detection tools using reads generated at five lengths (36, A; 50, B; 76, C; 100, D; 150, E) against test:ref pairs simulating six SCNA sizes and mapped to the chr1 reference sequence using five aligners. Each plot graphs the false positives (x axes) against the true positives (y axes). The size of the data point indicates the deviation in size of the detected SCNA from the simulated SCNA, the shape indicates the upstream aligner (Text S2). (PDF) [file pone.0047812.s004.pdf]

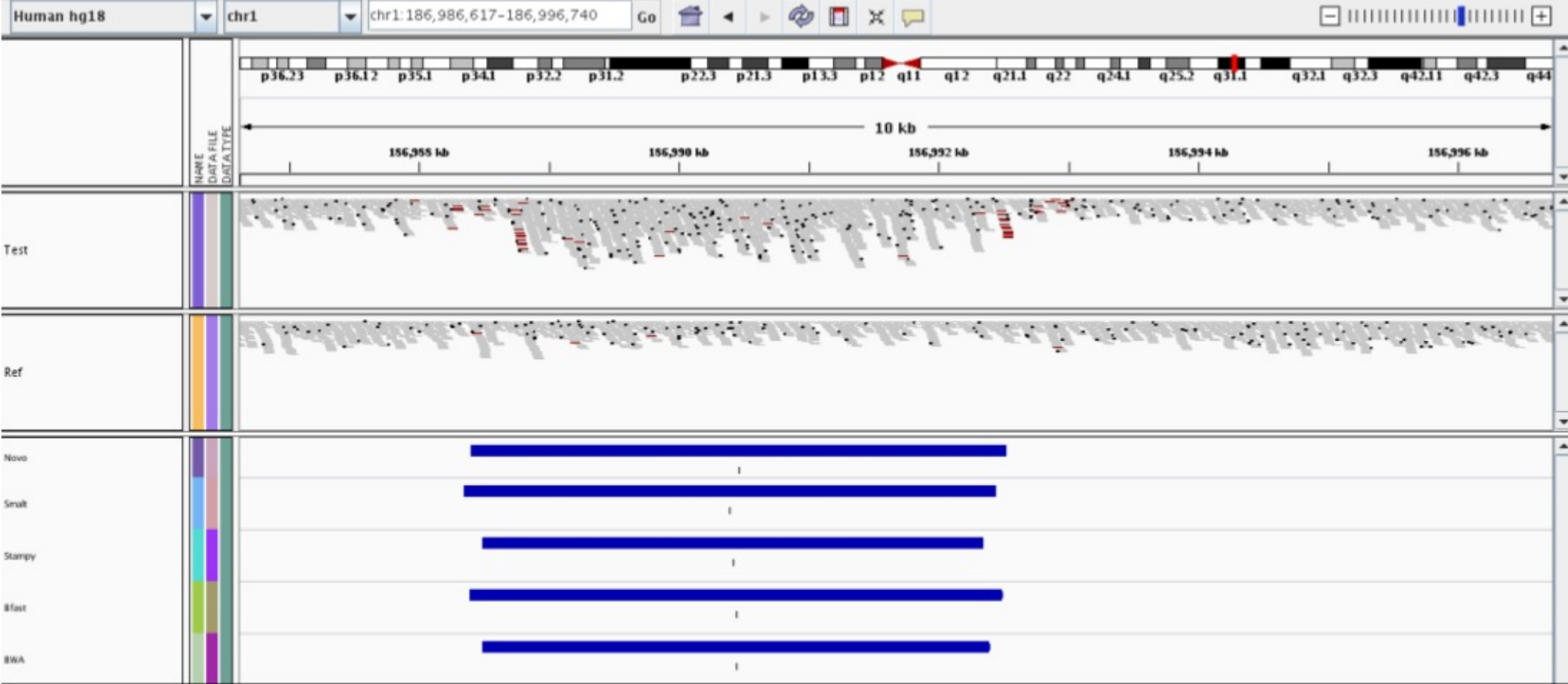

Supplement: Figure S5 — Performance comparison of COPS using different aligners. An IGV snapshot captures variation in SCNA boundaries detected by COPS using reads mapped by different upstream aligners. (PDF) [file pone.0047812.s005.pdf]

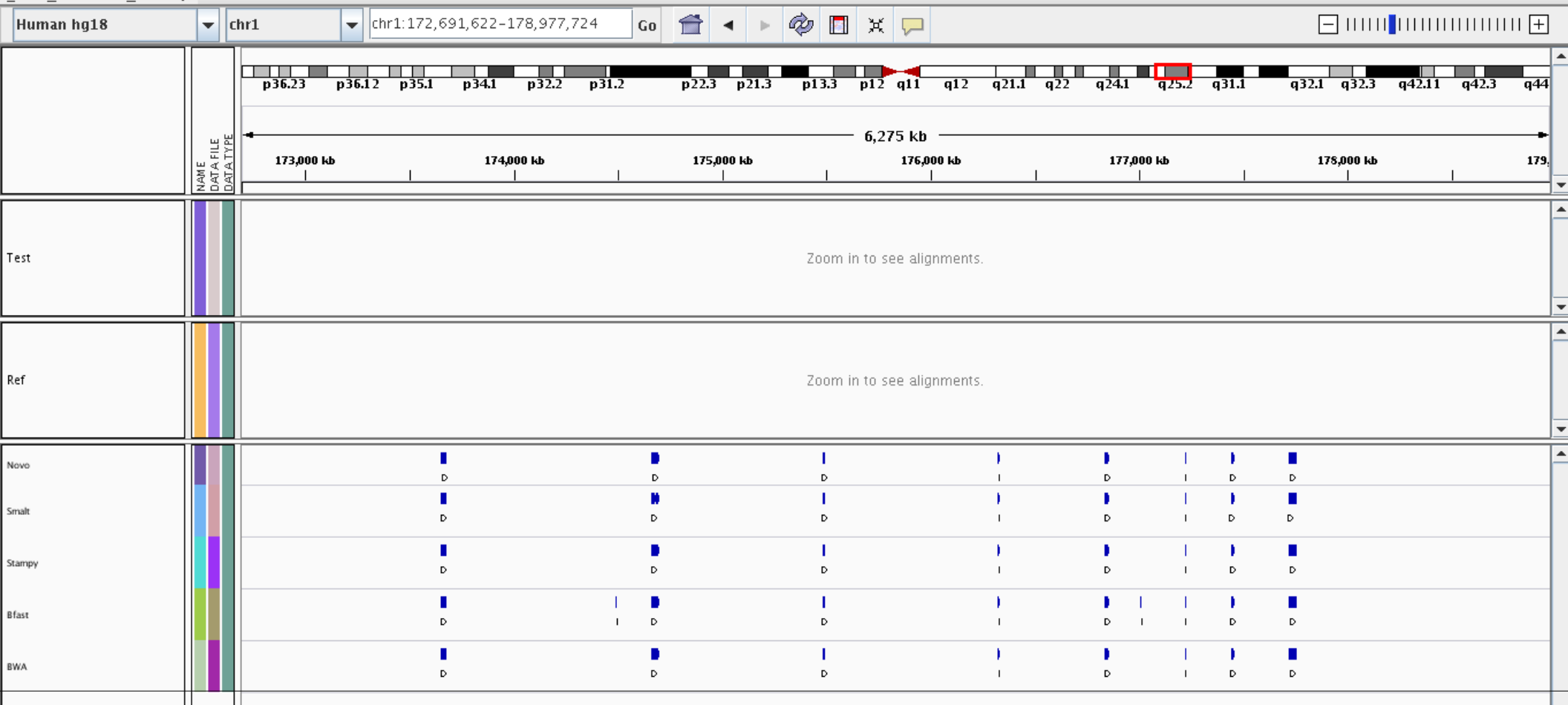

Supplement: Figure S6 — Performance comparison of COPS using different aligners. An IGV snapshot captures detection of false positive amplification-type SCNA events using COPS with Bfast-aligned reads. (PDF) [file pone.0047812.s006.pdf]

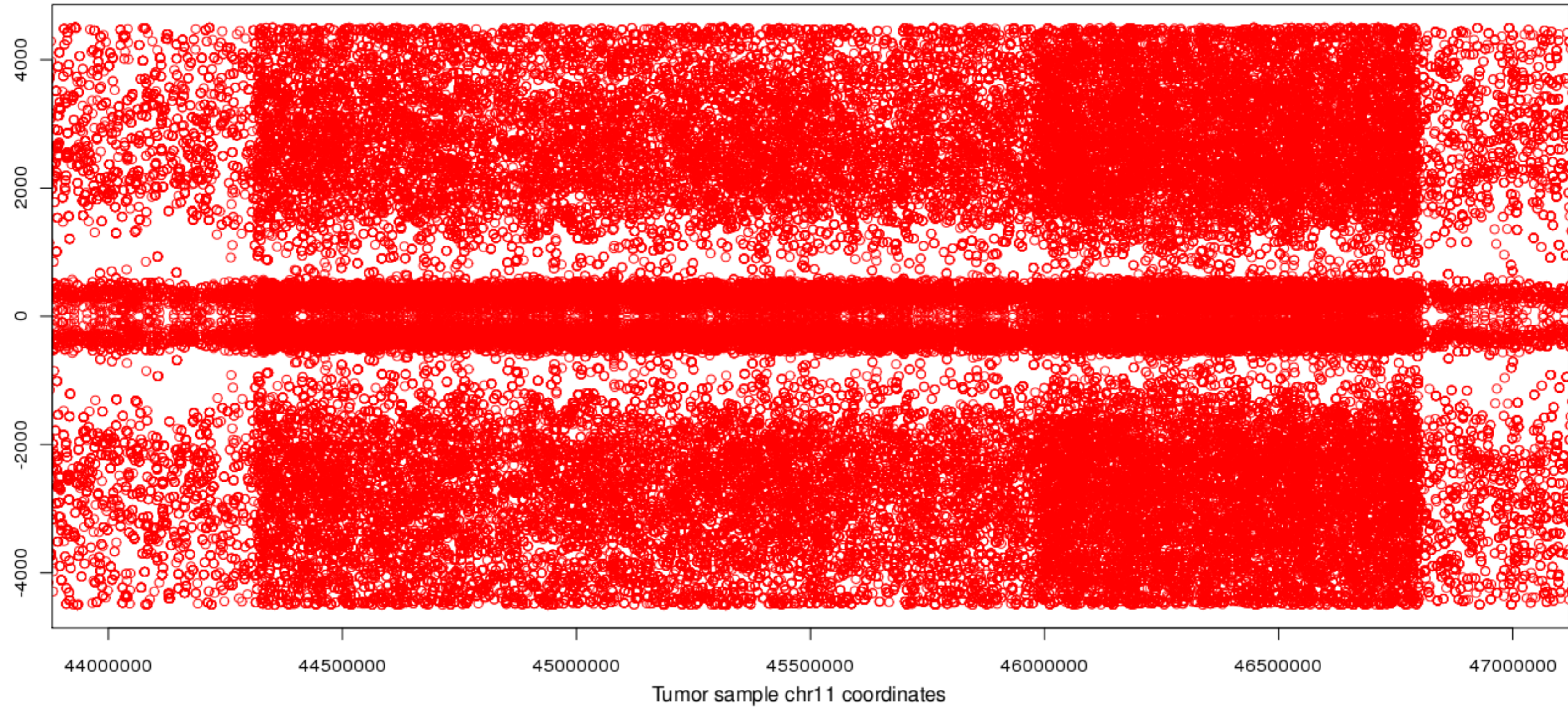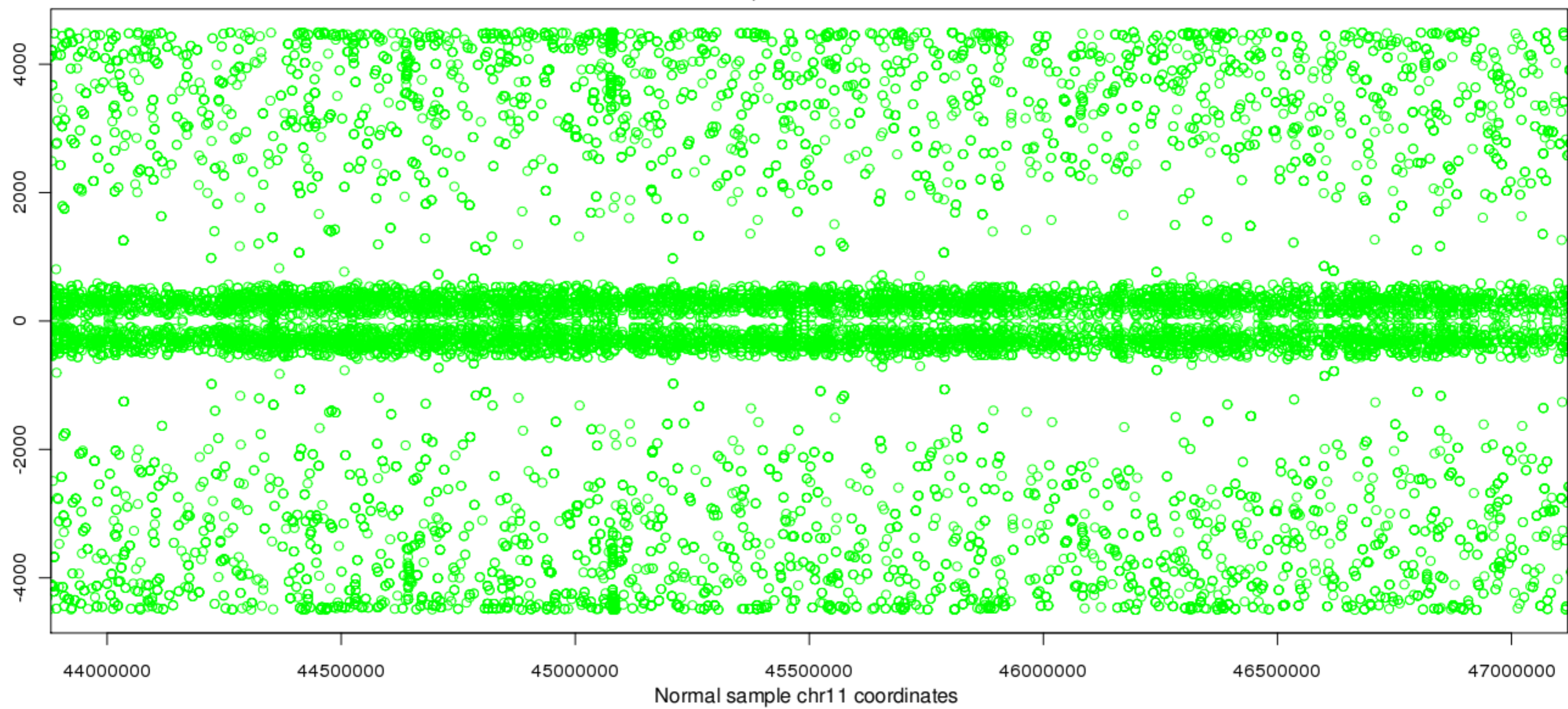

Supplement: Figure S7 — A differential density of anomalous reads near SCNA boundaries. The insert sizes of anomalously mapped paired reads below the expected insert size of 5000 nts including a standard deviation of 500 nts are plotted for a region of chromosome 11 harboring two amplification-type SCNAs, individually for the tumor and the normal sample. (PDF) [file pone.0047812.s007.pdf]
