# Supplementary material for: COPS: A Sensitive and Accurate Tool for Detecting Somatic Copy Number Alterations Using Short-Read Sequence Data from Paired Samples
Source: PLoS One. 2012 Oct 22;7(10):e47812. doi: 10.1371/journal.pone.0047812 (PMC3478291; doi:10.1371/journal.pone.0047812)
Supplement: Table S1 — Performance of COPS at varying bin sizes for binning read depths and step sizes for smoothening the log2ratios. (PDF) [file pone.0047812.s008.pdf]

Supporting Table 1. Performance of COPS at varying bin sizes for binning read depths and step sizes for smoothening the $log_2$ratios

A

| **0.05% (1-10kb)** | | | **36** | | | **50** | | | **76** | | | **100** | | | **150** | | |
|---|---|---|---|---|---|---|---|---|---|---|---|---|---|---|---|---|---|
| **Aligner** | **Bin size** | **Step size** | **TP** | **FP** | **SD** | **TP** | **FP** | **SD** | **TP** | **FP** | **SD** | **TP** | **FP** | **SD** | **TP** | **FP** | **SD** |
| Bfast | 60 | 3 | 2.94 | 0.19 | 98.65 | 100.00 | 0.05 | 7.84 | 100.00 | 0.08 | 7.87 | 2.94 | 0.27 | 98.80 | 100.00 | 0.07 | 7.50 |
| | | 4 | 2.94 | 0.19 | 98.65 | 100.00 | 0.06 | 7.35 | 100.00 | 0.08 | 7.87 | 2.94 | 0.27 | 98.80 | 100.00 | 0.07 | 7.50 |
| | | 5 | 2.94 | 0.19 | 98.65 | 100.00 | 0.08 | 6.16 | 100.00 | 0.08 | 7.87 | 2.94 | 0.27 | 98.80 | 100.00 | 0.07 | 7.50 |
| | | 6 | 2.94 | 0.19 | 98.65 | 100.00 | 0.08 | 6.09 | 100.00 | 0.08 | 7.87 | 2.94 | 0.27 | 98.80 | 100.00 | 0.07 | 7.50 |
| | 50 | 3 | 100.00 | 0.10 | 6.85 | 100.00 | 0.07 | 7.80 | 100.00 | 0.10 | 5.25 | 97.06 | 0.17 | 9.09 | 100.00 | 0.11 | 7.11 |
| | | 4 | 100.00 | 0.10 | 6.85 | 100.00 | 0.09 | 7.42 | 100.00 | 0.10 | 5.25 | 97.06 | 0.17 | 9.09 | 100.00 | 0.11 | 7.11 |
| | | 5 | 100.00 | 0.10 | 6.85 | 100.00 | 0.10 | 7.47 | 100.00 | 0.10 | 5.25 | 97.06 | 0.17 | 9.09 | 100.00 | 0.11 | 7.11 |
| | | 6 | 100.00 | 0.10 | 6.85 | 100.00 | 0.11 | 7.26 | 100.00 | 0.10 | 5.25 | 97.06 | 0.17 | 9.09 | 100.00 | 0.11 | 7.11 |
| BWA | 60 | 3 | 97.06 | 0.02 | 11.78 | 100.00 | 0.00 | 4.77 | 100.00 | 0.00 | 8.31 | 97.06 | 0.00 | 9.59 | 100.00 | 0.01 | 7.93 |
| | | 4 | 97.06 | 0.02 | 11.78 | 100.00 | 0.00 | 4.77 | 100.00 | 0.00 | 8.31 | 97.06 | 0.00 | 9.59 | 100.00 | 0.01 | 7.93 |
| | | 5 | 97.06 | 0.02 | 11.78 | 100.00 | 0.00 | 4.50 | 100.00 | 0.00 | 8.31 | 97.06 | 0.00 | 9.59 | 100.00 | 0.01 | 7.93 |
| | | 6 | 97.06 | 0.02 | 11.78 | 100.00 | 0.00 | 4.48 | 100.00 | 0.00 | 8.31 | 97.06 | 0.00 | 9.59 | 100.00 | 0.01 | 7.93 |
| | 50 | 3 | 97.06 | 0.03 | 11.20 | 100.00 | 0.00 | 5.22 | 100.00 | 0.00 | 5.22 | 97.06 | 0.00 | 8.95 | 100.00 | 0.03 | 7.31 |
| | | 4 | 97.06 | 0.03 | 11.20 | 100.00 | 0.00 | 4.94 | 100.00 | 0.00 | 5.22 | 97.06 | 0.00 | 8.95 | 100.00 | 0.03 | 7.31 |
| | | 5 | 97.06 | 0.03 | 11.20 | 100.00 | 0.00 | 4.08 | 100.00 | 0.00 | 5.22 | 97.06 | 0.00 | 8.95 | 100.00 | 0.03 | 7.31 |
| | | 6 | 97.06 | 0.03 | 11.20 | 100.00 | 0.00 | 3.86 | 100.00 | 0.00 | 5.22 | 97.06 | 0.00 | 8.95 | 100.00 | 0.03 | 7.31 |
| Novo | 60 | 3 | 2.94 | 0.09 | 98.65 | 97.06 | 0.00 | 8.69 | 100.00 | 0.01 | 7.95 | 2.94 | 0.10 | 98.86 | 97.06 | 0.04 | 8.67 |
| | | 4 | 2.94 | 0.09 | 98.65 | 100.00 | 0.00 | 4.13 | 100.00 | 0.01 | 7.95 | 2.94 | 0.10 | 98.86 | 97.06 | 0.04 | 8.67 |
| | | 5 | 2.94 | 0.09 | 98.65 | 100.00 | 0.00 | 4.40 | 100.00 | 0.01 | 7.95 | 2.94 | 0.10 | 98.86 | 97.06 | 0.04 | 8.67 |
| | | 6 | 2.94 | 0.09 | 98.65 | 100.00 | 0.00 | 4.14 | 100.00 | 0.01 | 7.95 | 2.94 | 0.10 | 98.86 | 97.06 | 0.04 | 8.67 |
| | 50 | 3 | 100.00 | 0.00 | 3.63 | 100.00 | 0.01 | 4.25 | 100.00 | 0.02 | 5.07 | 97.06 | 0.02 | 8.78 | 100.00 | 0.07 | 7.26 |
| | | 4 | 100.00 | 0.00 | 3.63 | 100.00 | 0.01 | 3.80 | 100.00 | 0.02 | 5.07 | 97.06 | 0.02 | 8.78 | 100.00 | 0.07 | 7.26 |
| | | 5 | 100.00 | 0.00 | 3.63 | 100.00 | 0.01 | 4.70 | 100.00 | 0.02 | 5.07 | 97.06 | 0.02 | 8.78 | 100.00 | 0.07 | 7.26 |
| | | 6 | 100.00 | 0.00 | 3.63 | 100.00 | 0.01 | 3.93 | 100.00 | 0.02 | 5.07 | 97.06 | 0.02 | 8.78 | 100.00 | 0.07 | 7.26 |
| Smalt | 60 | 3 | 100.00 | 2.46 | 8.76 | 100.00 | 0.38 | 6.59 | 100.00 | 0.02 | 8.52 | 94.12 | 0.00 | 11.68 | 100.00 | 0.02 | 7.65 |
| | | 4 | 100.00 | 2.46 | 8.76 | 100.00 | 0.42 | 6.78 | 100.00 | 0.02 | 8.52 | 94.12 | 0.00 | 11.68 | 100.00 | 0.02 | 7.65 |
| | | 5 | 100.00 | 2.46 | 8.76 | 100.00 | 0.48 | 7.01 | 100.00 | 0.02 | 8.52 | 94.12 | 0.00 | 11.68 | 100.00 | 0.02 | 7.65 |
| | | 6 | 100.00 | 2.46 | 8.76 | 100.00 | 0.52 | 6.92 | 100.00 | 0.02 | 8.52 | 94.12 | 0.00 | 11.68 | 100.00 | 0.02 | 7.65 |

| | | | | | | | | | | | | | | | | | |
|---|---|---|---|---|---|---|---|---|---|---|---|---|---|---|---|---|---|
| | 50 | 3 | 100.00 | 2.89 | 7.08 | 100.00 | 0.60 | 5.39 | 100.00 | 0.02 | 5.70 | 97.06 | 0.03 | 9.04 | 100.00 | 0.05 | 7.29 |
| | | 4 | 100.00 | 2.89 | 7.08 | 100.00 | 0.66 | 5.47 | 100.00 | 0.02 | 5.70 | 97.06 | 0.03 | 9.04 | 100.00 | 0.05 | 7.29 |
| | | 5 | 100.00 | 2.89 | 7.08 | 100.00 | 0.70 | 5.22 | 100.00 | 0.02 | 5.70 | 97.06 | 0.03 | 9.04 | 100.00 | 0.05 | 7.29 |
| | | 6 | 100.00 | 2.89 | 7.08 | 100.00 | 0.75 | 5.52 | 100.00 | 0.02 | 5.70 | 97.06 | 0.03 | 9.04 | 100.00 | 0.05 | 7.29 |
| Stampy | 60 | 3 | 94.12 | 0.00 | 11.35 | 100.00 | 0.00 | 7.71 | 100.00 | 0.01 | 8.00 | 94.12 | 0.00 | 10.23 | 100.00 | 0.05 | 8.52 |
| | | 4 | 94.12 | 0.00 | 11.35 | 100.00 | 0.00 | 7.37 | 100.00 | 0.01 | 8.00 | 94.12 | 0.00 | 10.23 | 100.00 | 0.05 | 8.52 |
| | | 5 | 94.12 | 0.00 | 11.35 | 100.00 | 0.00 | 6.92 | 100.00 | 0.01 | 8.00 | 94.12 | 0.00 | 10.23 | 100.00 | 0.05 | 8.52 |
| | | 6 | 94.12 | 0.00 | 11.35 | 100.00 | 0.00 | 6.79 | 100.00 | 0.01 | 8.00 | 94.12 | 0.00 | 10.23 | 100.00 | 0.05 | 8.52 |
| | 50 | 3 | 100.00 | 0.00 | 9.18 | 100.00 | 0.00 | 7.60 | 100.00 | 0.01 | 5.37 | 97.06 | 0.00 | 8.67 | 100.00 | 0.08 | 9.24 |
| | | 4 | 100.00 | 0.00 | 9.18 | 100.00 | 0.00 | 7.38 | 100.00 | 0.01 | 5.37 | 97.06 | 0.00 | 8.67 | 100.00 | 0.08 | 9.24 |
| | | 5 | 100.00 | 0.00 | 9.18 | 100.00 | 0.00 | 7.01 | 100.00 | 0.01 | 5.37 | 97.06 | 0.00 | 8.67 | 100.00 | 0.08 | 9.24 |
| | | 6 | 100.00 | 0.00 | 9.18 | 100.00 | 0.00 | 6.75 | 100.00 | 0.01 | 5.37 | 97.06 | 0.00 | 8.67 | 100.00 | 0.08 | 9.20 |

B

| **0.10% (10-50kb)** | | | **36** | | | **50** | | | **76** | | | **100** | | | **150** | | |
|---|---|---|---|---|---|---|---|---|---|---|---|---|---|---|---|---|---|
| **Aligner** | **Bin size** | **Step size** | **TP** | **FP** | **SD** | **TP** | **FP** | **SD** | **TP** | **FP** | **SD** | **TP** | **FP** | **SD** | **TP** | **FP** | **SD** |
| Bfast | 60 | 3 | 100.00 | 0.82 | 2.08 | 100.00 | 0.36 | 2.34 | 100.00 | 0.14 | 2.62 | 100.00 | 0.27 | 3.32 | 100.00 | 0.22 | 3.06 |
| | | 4 | 100.00 | 0.82 | 2.08 | 100.00 | 0.36 | 2.34 | 100.00 | 0.14 | 2.62 | 100.00 | 0.27 | 3.32 | 100.00 | 0.22 | 3.06 |
| | | 5 | 100.00 | 0.82 | 2.08 | 100.00 | 0.36 | 2.34 | 100.00 | 0.14 | 2.62 | 100.00 | 0.27 | 3.32 | 100.00 | 0.22 | 3.06 |
| | | 6 | 100.00 | 0.82 | 2.08 | 100.00 | 0.36 | 2.34 | 100.00 | 0.14 | 2.62 | 100.00 | 0.27 | 3.32 | 100.00 | 0.22 | 3.06 |
| | 50 | 3 | 100.00 | 0.84 | 1.91 | 100.00 | 0.37 | 1.65 | 100.00 | 0.16 | 2.32 | 100.00 | 0.29 | 3.44 | 100.00 | 0.27 | 2.92 |
| | | 4 | 100.00 | 0.84 | 1.91 | 100.00 | 0.37 | 1.65 | 100.00 | 0.16 | 2.32 | 100.00 | 0.29 | 3.44 | 100.00 | 0.27 | 2.92 |
| | | 5 | 100.00 | 0.84 | 1.91 | 100.00 | 0.37 | 1.65 | 100.00 | 0.16 | 2.32 | 100.00 | 0.29 | 3.44 | 100.00 | 0.27 | 2.92 |
| | | 6 | 100.00 | 0.84 | 1.91 | 100.00 | 0.37 | 1.65 | 100.00 | 0.16 | 2.32 | 100.00 | 0.29 | 3.44 | 100.00 | 0.27 | 2.92 |
| BWA | 60 | 3 | 100.00 | 0.02 | 2.38 | 100.00 | 0.00 | 2.08 | 100.00 | 0.00 | 2.26 | 100.00 | 0.00 | 2.32 | 100.00 | 0.01 | 3.03 |
| | | 4 | 100.00 | 0.02 | 2.38 | 100.00 | 0.00 | 2.08 | 100.00 | 0.00 | 2.26 | 100.00 | 0.00 | 2.32 | 100.00 | 0.01 | 3.03 |
| | | 5 | 100.00 | 0.02 | 2.38 | 100.00 | 0.00 | 2.08 | 100.00 | 0.00 | 2.26 | 100.00 | 0.00 | 2.32 | 100.00 | 0.01 | 3.03 |
| | | 6 | 100.00 | 0.02 | 2.38 | 100.00 | 0.00 | 2.08 | 100.00 | 0.00 | 2.26 | 100.00 | 0.00 | 2.32 | 100.00 | 0.01 | 3.03 |
| | 50 | 3 | 100.00 | 0.03 | 2.12 | 100.00 | 0.00 | 2.25 | 100.00 | 0.00 | 2.07 | 100.00 | 0.00 | 2.45 | 100.00 | 0.04 | 2.60 |
| | | 4 | 100.00 | 0.03 | 2.12 | 100.00 | 0.00 | 2.25 | 100.00 | 0.00 | 2.07 | 100.00 | 0.00 | 2.45 | 100.00 | 0.04 | 2.60 |
| | | 5 | 100.00 | 0.03 | 2.12 | 100.00 | 0.00 | 2.25 | 100.00 | 0.00 | 2.07 | 100.00 | 0.00 | 2.45 | 100.00 | 0.04 | 2.60 |

| | | | | | | | | | | | | | | | | | |
|---|---|---|---|---|---|---|---|---|---|---|---|---|---|---|---|---|---|
| | | 6 | 100.00 | 0.03 | 2.12 | 100.00 | 0.00 | 2.25 | 100.00 | 0.00 | 2.07 | 100.00 | 0.00 | 2.45 | 100.00 | 0.04 | 2.60 |
| Novo | 60 | 3 | 100.00 | 0.00 | 1.89 | 100.00 | 0.00 | 1.25 | 100.00 | 0.01 | 2.51 | 100.00 | 0.01 | 2.37 | 100.00 | 0.04 | 2.70 |
| | | 4 | 100.00 | 0.00 | 1.89 | 100.00 | 0.00 | 1.25 | 100.00 | 0.01 | 2.51 | 100.00 | 0.01 | 2.37 | 100.00 | 0.04 | 2.70 |
| | | 5 | 100.00 | 0.00 | 1.89 | 100.00 | 0.00 | 1.25 | 100.00 | 0.01 | 2.51 | 100.00 | 0.01 | 2.37 | 100.00 | 0.04 | 2.70 |
| | | 6 | 100.00 | 0.00 | 1.89 | 100.00 | 0.00 | 1.25 | 100.00 | 0.01 | 2.51 | 100.00 | 0.01 | 2.37 | 100.00 | 0.04 | 2.70 |
| | 50 | 3 | 100.00 | 0.00 | 1.51 | 100.00 | 0.00 | 1.51 | 100.00 | 0.01 | 2.41 | 100.00 | 0.02 | 2.50 | 100.00 | 0.08 | 2.26 |
| | | 4 | 100.00 | 0.00 | 1.51 | 100.00 | 0.00 | 1.51 | 100.00 | 0.01 | 2.41 | 100.00 | 0.02 | 2.50 | 100.00 | 0.08 | 2.26 |
| | | 5 | 100.00 | 0.00 | 1.51 | 100.00 | 0.00 | 1.51 | 100.00 | 0.01 | 2.41 | 100.00 | 0.02 | 2.50 | 100.00 | 0.08 | 2.26 |
| | | 6 | 100.00 | 0.00 | 1.51 | 100.00 | 0.00 | 1.51 | 100.00 | 0.01 | 2.41 | 100.00 | 0.02 | 2.50 | 100.00 | 0.08 | 2.26 |
| Smalt | 60 | 3 | 100.00 | 0.03 | 1.48 | 100.00 | 0.01 | 3.35 | 26.67 | 88.53 | 207.56 | 100.00 | 0.00 | 3.86 | 100.00 | 0.03 | 2.79 |
| | | 4 | 100.00 | 0.03 | 1.48 | 100.00 | 0.01 | 3.35 | 26.67 | 88.53 | 207.56 | 100.00 | 0.00 | 3.86 | 100.00 | 0.03 | 2.79 |
| | | 5 | 100.00 | 0.03 | 1.48 | 100.00 | 0.01 | 3.35 | 26.67 | 88.53 | 207.56 | 100.00 | 0.00 | 3.86 | 100.00 | 0.03 | 2.79 |
| | | 6 | 100.00 | 0.03 | 1.48 | 100.00 | 0.01 | 3.35 | 26.67 | 88.53 | 207.56 | 100.00 | 0.00 | 3.86 | 100.00 | 0.03 | 2.79 |
| | 50 | 3 | 100.00 | 0.04 | 2.23 | 100.00 | 0.06 | 3.11 | 26.67 | 88.41 | 202.08 | 100.00 | 0.00 | 3.66 | 100.00 | 0.18 | 2.73 |
| | | 4 | 100.00 | 0.04 | 2.23 | 100.00 | 0.06 | 3.11 | 26.67 | 88.41 | 202.08 | 100.00 | 0.00 | 3.66 | 100.00 | 0.18 | 2.73 |
| | | 5 | 100.00 | 0.04 | 2.23 | 100.00 | 0.06 | 3.11 | 26.67 | 88.41 | 202.08 | 100.00 | 0.00 | 3.66 | 100.00 | 0.18 | 2.73 |
| | | 6 | 100.00 | 0.04 | 2.23 | 100.00 | 0.06 | 3.11 | 26.67 | 88.41 | 202.08 | 100.00 | 0.00 | 3.66 | 100.00 | 0.18 | 2.73 |
| Stampy | 60 | 3 | 100.00 | 0.00 | 2.08 | 100.00 | 0.00 | 1.55 | 100.00 | 0.00 | 2.54 | 100.00 | 0.01 | 2.43 | 100.00 | 0.06 | 3.24 |
| | | 4 | 100.00 | 0.00 | 2.08 | 100.00 | 0.00 | 1.55 | 100.00 | 0.00 | 2.54 | 100.00 | 0.01 | 2.43 | 100.00 | 0.06 | 3.24 |
| | | 5 | 100.00 | 0.00 | 2.08 | 100.00 | 0.00 | 1.55 | 100.00 | 0.00 | 2.54 | 100.00 | 0.01 | 2.43 | 100.00 | 0.06 | 3.24 |
| | | 6 | 100.00 | 0.00 | 2.08 | 100.00 | 0.00 | 1.55 | 100.00 | 0.00 | 2.54 | 100.00 | 0.01 | 2.43 | 100.00 | 0.06 | 3.24 |
| | 50 | 3 | 100.00 | 0.00 | 1.89 | 100.00 | 0.00 | 2.33 | 100.00 | 0.01 | 2.22 | 100.00 | 0.01 | 3.43 | 100.00 | 0.09 | 3.09 |
| | | 4 | 100.00 | 0.00 | 1.89 | 100.00 | 0.00 | 2.33 | 100.00 | 0.01 | 2.22 | 100.00 | 0.01 | 3.43 | 100.00 | 0.09 | 3.09 |
| | | 5 | 100.00 | 0.00 | 1.89 | 100.00 | 0.00 | 2.33 | 100.00 | 0.01 | 2.22 | 100.00 | 0.01 | 3.43 | 100.00 | 0.09 | 3.09 |
| | | 6 | 100.00 | 0.00 | 1.89 | 100.00 | 0.00 | 2.33 | 100.00 | 0.01 | 2.22 | 100.00 | 0.01 | 3.43 | 100.00 | 0.09 | 3.09 |

C

| **1.50% (1-10kb)** | | | **36** | | | **50** | | | **76** | | | **100** | | | **150** | | |
|---|---|---|---|---|---|---|---|---|---|---|---|---|---|---|---|---|---|
| **Aligner** | **Bin size** | **Step size** | **TP** | **FP** | **SD** | **TP** | **FP** | **SD** | **TP** | **FP** | **SD** | **TP** | **FP** | **SD** | **TP** | **FP** | **SD** |
| Bfast | 60 | 3 | 87.73 | 0.78 | 15.00 | 87.49 | 0.77 | 14.86 | 87.81 | 0.72 | 14.63 | 87.81 | 0.53 | 16.15 | 87.57 | 0.38 | 17.62 |
| | | 4 | 87.73 | 0.78 | 15.00 | 87.49 | 0.77 | 14.86 | 87.81 | 0.72 | 14.63 | 87.81 | 0.53 | 16.15 | 87.57 | 0.38 | 17.62 |
| | | 5 | 87.73 | 0.78 | 15.00 | 87.49 | 0.77 | 14.86 | 87.81 | 0.72 | 14.63 | 87.81 | 0.53 | 16.15 | 87.57 | 0.38 | 17.62 |

| | | | | | | | | | | | | | | | | | |
|---|---|---|---|---|---|---|---|---|---|---|---|---|---|---|---|---|---|
| | | 6 | 87.73 | 0.78 | 15.00 | 87.49 | 0.77 | 14.86 | 87.81 | 0.72 | 14.63 | 87.81 | 0.53 | 16.15 | 87.57 | 0.38 | 17.62 |
| | 50 | 3 | 90.62 | 0.80 | 13.72 | 90.94 | 0.79 | 13.21 | 91.26 | 0.76 | 13.06 | 90.86 | 0.56 | 14.74 | 90.70 | 0.43 | 16.04 |
| | | 4 | 90.62 | 0.80 | 13.72 | 90.94 | 0.79 | 13.21 | 91.26 | 0.76 | 13.06 | 90.86 | 0.56 | 14.74 | 90.70 | 0.43 | 16.04 |
| | | 5 | 90.62 | 0.80 | 13.72 | 90.94 | 0.79 | 13.21 | 91.26 | 0.76 | 13.06 | 90.86 | 0.56 | 14.74 | 90.70 | 0.43 | 16.04 |
| | | 6 | 90.62 | 0.80 | 13.72 | 90.94 | 0.79 | 13.21 | 91.26 | 0.76 | 13.06 | 90.86 | 0.56 | 14.74 | 90.70 | 0.43 | 16.04 |
| BWA | 60 | 3 | 86.69 | 0.05 | 14.01 | 87.73 | 0.02 | 13.02 | 88.45 | 0.02 | 13.35 | 88.37 | 0.02 | 14.95 | 87.97 | 0.04 | 16.99 |
| | | 4 | 86.69 | 0.05 | 14.01 | 87.73 | 0.02 | 13.02 | 88.45 | 0.02 | 13.35 | 88.37 | 0.02 | 14.95 | 87.97 | 0.04 | 16.99 |
| | | 5 | 86.69 | 0.05 | 14.01 | 87.73 | 0.02 | 13.02 | 88.45 | 0.02 | 13.35 | 88.37 | 0.02 | 14.95 | 87.97 | 0.04 | 16.99 |
| | | 6 | 86.69 | 0.05 | 14.01 | 87.73 | 0.02 | 13.02 | 88.45 | 0.02 | 13.35 | 88.37 | 0.02 | 14.95 | 87.97 | 0.04 | 16.99 |
| | 50 | 3 | 90.54 | 0.06 | 12.14 | 90.94 | 0.03 | 11.41 | 91.26 | 0.03 | 11.73 | 91.26 | 0.04 | 13.35 | 91.18 | 0.08 | 15.39 |
| | | 4 | 90.54 | 0.06 | 12.14 | 90.94 | 0.03 | 11.41 | 91.26 | 0.03 | 11.73 | 91.26 | 0.04 | 13.35 | 91.18 | 0.08 | 15.39 |
| | | 5 | 90.54 | 0.06 | 12.14 | 90.94 | 0.03 | 11.41 | 91.26 | 0.03 | 11.73 | 91.26 | 0.04 | 13.35 | 91.18 | 0.08 | 15.39 |
| | | 6 | 90.54 | 0.06 | 12.14 | 90/94 | 0.03 | 11.41 | 91.26 | 0.03 | 11.73 | 91.26 | 0.04 | 13.35 | 91.18 | 0.08 | 15.39 |
| Novo | 60 | 3 | 88.29 | 0.01 | 11.39 | 88.05 | 0.01 | 11.91 | 88.05 | 0.53 | 13.48 | 88.37 | 0.44 | 15.45 | 88.05 | 0.32 | 17.67 |
| | | 4 | 88.29 | 0.01 | 11.39 | 88.05 | 0.01 | 11.91 | 88.05 | 0.53 | 13.48 | 88.37 | 0.44 | 15.45 | 88.05 | 0.32 | 17.67 |
| | | 5 | 88.29 | 0.01 | 11.39 | 88.05 | 0.01 | 11.91 | 88.05 | 0.53 | 13.48 | 88.37 | 0.44 | 15.45 | 88.05 | 0.32 | 17.67 |
| | | 6 | 88.29 | 0.01 | 11.39 | 88.05 | 0.01 | 11.91 | 88.05 | 0.53 | 13.48 | 88.37 | 0.44 | 15.45 | 88.05 | 0.32 | 17.67 |
| | 50 | 3 | 91.50 | 0.02 | 10.05 | 91.66 | 0.03 | 10.38 | 91.50 | 0.55 | 11.77 | 91.26 | 0.45 | 14.01 | 91.42 | 0.37 | 15.97 |
| | | 4 | 91.50 | 0.02 | 10.05 | 91.66 | 0.03 | 10.38 | 91.50 | 0.55 | 11.77 | 91.26 | 0.45 | 14.01 | 91.42 | 0.37 | 15.97 |
| | | 5 | 91.50 | 0.02 | 10.05 | 91.66 | 0.03 | 10.38 | 91.50 | 0.55 | 11.77 | 91.26 | 0.45 | 14.01 | 91.42 | 0.37 | 15.97 |
| | | 6 | 91.50 | 0.02 | 10.05 | 91.66 | 0.03 | 10.38 | 91.50 | 0.55 | 11.77 | 91.26 | 0.45 | 14.01 | 91.42 | 0.37 | 15.97 |
| Smalt | 60 | 3 | 87.81 | 0.04 | 12.91 | 87.73 | 0.02 | 13.09 | 87.81 | 0.02 | 13.97 | 87.41 | 0.02 | 16.48 | 86.77 | 0.04 | 17.60 |
| | | 4 | 87.81 | 0.04 | 12.91 | 87.73 | 0.02 | 13.09 | 87.81 | 0.02 | 13.97 | 87.41 | 0.02 | 16.48 | 86.77 | 0.04 | 17.60 |
| | | 5 | 87.81 | 0.00 | 12.91 | 87.73 | 0.02 | 13.09 | 87.81 | 0.02 | 13.97 | 87.41 | 0.02 | 16.48 | 86.77 | 0.04 | 17.60 |
| | | 6 | 87.81 | 0.00 | 12.91 | 87.73 | 0.02 | 13.09 | 87.81 | 0.02 | 13.97 | 87.41 | 0.02 | 16.48 | 86.77 | 0.04 | 17.60 |
| | 50 | 3 | 91.66 | 0.00 | 10.94 | 91.26 | 0.03 | 11.43 | 90.62 | 0.02 | 12.49 | 89.90 | 0.03 | 15.05 | 89.90 | 0.08 | 16.22 |
| | | 4 | 91.66 | 0.00 | 10.94 | 91.26 | 0.03 | 11.43 | 90.62 | 0.02 | 12.49 | 89.90 | 0.03 | 15.05 | 89.90 | 0.08 | 16.22 |
| | | 5 | 91.66 | 0.00 | 10.94 | 91.26 | 0.03 | 11.43 | 90.62 | 0.02 | 12.49 | 89.90 | 0.03 | 15.05 | 89.90 | 0.08 | 16.22 |
| | | 6 | 91.66 | 0.00 | 10.94 | 91.26 | 0.03 | 11.43 | 90.62 | 0.02 | 12.49 | 89.90 | 0.03 | 15.05 | 89.90 | 0.08 | 16.22 |
| Stampy | 60 | 3 | 87.33 | 0.00 | 12.96 | 87.73 | 0.03 | 12.79 | 88.13 | 0.03 | 13.35 | 88.05 | 0.03 | 14.92 | 87.33 | 0.04 | 17.29 |
| | | 4 | 87.33 | 0.00 | 12.96 | 87.73 | 0.03 | 12.79 | 88.13 | 0.03 | 13.35 | 88.05 | 0.03 | 14.92 | 87.33 | 0.04 | 17.29 |
| | | 5 | 87.33 | 0.00 | 12.96 | 87.73 | 0.03 | 12.79 | 88.13 | 0.03 | 13.35 | 88.05 | 0.03 | 14.92 | 87.33 | 0.04 | 17.29 |
| | | 6 | 87.33 | 0.00 | 12.96 | 87.73 | 0.03 | 12.79 | 88.13 | 0.03 | 13.35 | 88.05 | 0.03 | 14.92 | 87.33 | 0.04 | 17.29 |

| | | | | | | | | | | | | | | | | | |
|---|---|---|---|---|---|---|---|---|---|---|---|---|---|---|---|---|---|
| | 50 | 3 | 90.46 | 0.00 | 11.34 | 91.18 | 0.03 | 10.95 | 91.02 | 0.03 | 11.77 | 90/78 | 0.04 | 13.45 | 90.54 | 0.08 | 15.76 |
| | | 4 | 90.46 | 0.00 | 11.34 | 91.18 | 0.03 | 10.95 | 91.02 | 0.03 | 11.77 | 90.78 | 0.04 | 13.45 | 90.54 | 0.08 | 15.76 |
| | | 5 | 90.46 | 0.00 | 11.34 | 91.18 | 0.03 | 10.95 | 91.02 | 0.03 | 11.77 | 90.78 | 0.04 | 13.45 | 90.54 | 0.08 | 15.76 |
| | | 6 | 90.46 | 0.00 | 11.34 | 91.18 | 0.03 | 10.95 | 91.02 | 0.03 | 11.77 | 90.78 | 0.04 | 13.45 | 90.54 | 0.08 | 15.76 |

D

| **1.50% (10-50kb)** | | | **36** | | | **50** | | | **76** | | | **100** | | | **150** | | |
|---|---|---|---|---|---|---|---|---|---|---|---|---|---|---|---|---|---|
| **Aligner** | **Bin size** | **Step size** | **TP** | **FP** | **SD** | **TP** | **FP** | **SD** | **TP** | **FP** | **SD** | **TP** | **FP** | **SD** | **TP** | **FP** | **SD** |
| Bfast | 60 | 3 | 95.83 | 0.89 | 13.47 | 95.83 | 0.85 | 13.56 | 98.33 | 0.66 | 10.81 | 97.08 | 0.56 | 11.55 | 98.33 | 0.43 | 13.17 |
| | | 4 | 95.83 | 0.89 | 13.47 | 95.83 | 0.85 | 13.56 | 98.33 | 0.66 | 10.81 | 97.08 | 0.56 | 11.55 | 98.33 | 0.43 | 13.17 |
| | | 5 | 95.83 | 0.89 | 13.47 | 95.83 | 0.85 | 13.56 | 98.33 | 0.66 | 10.81 | 97.08 | 0.56 | 11.55 | 98.33 | 0.43 | 13.17 |
| | | 6 | 95.83 | 0.89 | 13.47 | 95.83 | 0.85 | 13.56 | 98.33 | 0.66 | 10.81 | 97.08 | 0.56 | 11.55 | 98.33 | 0.43 | 13.17 |
| | 50 | 3 | 95.83 | 0.90 | 13.35 | 96.25 | 0.88 | 13.47 | 98.33 | 0.68 | 10.72 | 97.08 | 0.59 | 11.55 | 98.33 | 0.47 | 12.90 |
| | | 4 | 95.83 | 0.90 | 13.35 | 96.25 | 0.88 | 13.47 | 98.33 | 0.68 | 10.72 | 97.08 | 0.59 | 11.55 | 98.33 | 0.47 | 12.90 |
| | | 5 | 95.83 | 0.90 | 13.35 | 96.25 | 0.88 | 13.47 | 98.33 | 0.68 | 10.72 | 97.08 | 0.59 | 11.55 | 98.33 | 0.47 | 12.90 |
| | | 6 | 95.83 | 0.90 | 13.35 | 96.25 | 0.88 | 13.47 | 98.33 | 0.68 | 10.71 | 97.08 | 0.59 | 11.55 | 98.33 | 0.47 | 12.90 |
| BWA | 60 | 3 | 96.67 | 0.02 | 13.03 | 95.42 | 0.16 | 13.30 | 97.92 | 0.00 | 11.18 | 97.08 | 0.00 | 11.70 | 98.33 | 0.01 | 12.98 |
| | | 4 | 96.67 | 0.02 | 13.03 | 95.42 | 0.16 | 13.20 | 97.92 | 0.00 | 11.18 | 97.08 | 0.00 | 11.70 | 98.33 | 0.01 | 12.98 |
| | | 5 | 96.67 | 0.02 | 13.03 | 95.42 | 0.16 | 13.20 | 97.92 | 0.00 | 11.18 | 97.08 | 0.00 | 11.70 | 98.33 | 0.01 | 12.98 |
| | | 6 | 96.67 | 0.02 | 13.03 | 95.42 | 0.16 | 13.20 | 97.92 | 0.00 | 11.18 | 97.08 | 0.00 | 11.70 | 98.33 | 0.01 | 12.98 |
| | 50 | 3 | 96.67 | 0.03 | 12.98 | 95.83 | 0.00 | 13.01 | 98.33 | 0.00 | 10.98 | 97.50 | 0.00 | 11.74 | 98.33 | 0.04 | 12.75 |
| | | 4 | 96.67 | 0.03 | 12.98 | 95.83 | 0.00 | 13.01 | 98.33 | 0.00 | 10.98 | 97.50 | 0.00 | 11.74 | 98.33 | 0.04 | 12.75 |
| | | 5 | 96.67 | 0.03 | 12.98 | 95.83 | 0.00 | 13.01 | 98.33 | 0.00 | 10.98 | 97.50 | 0.00 | 11.74 | 98.33 | 0.04 | 12.75 |
| | | 6 | 96.67 | 0.03 | 12.98 | 95.83 | 0.00 | 13.01 | 98.33 | 0.00 | 10.98 | 97.50 | 0.00 | 11.74 | 98.33 | 0.04 | 12.75 |
| Novo | 60 | 3 | 95.83 | 0.94 | 13.74 | 96.25 | 0.65 | 13.05 | 77.92 | 44.13 | 71.86 | 97.08 | 0.43 | 11.52 | 98.33 | 0.03 | 12.88 |
| | | 4 | 95.83 | 0.94 | 13.74 | 96.25 | 0.65 | 13.05 | 77.92 | 44.13 | 71.86 | 97.08 | 0.43 | 11.52 | 98.33 | 0.03 | 12.88 |
| | | 5 | 95.83 | 0.94 | 13.74 | 96.25 | 0.65 | 13.05 | 77.92 | 44.13 | 71.86 | 97.08 | 0.43 | 11.52 | 98.33 | 0.03 | 12.88 |
| | | 6 | 95.83 | 0.94 | 13.74 | 96.25 | 0.65 | 13.05 | 77.92 | 44.13 | 71.86 | 97.08 | 0.43 | 11.52 | 98.33 | 0.03 | 12.88 |
| | 50 | 3 | 96.25 | 0.97 | 13.49 | 96.25 | 0.66 | 12.91 | 82.50 | 45.99 | 71.72 | 97.08 | 0.45 | 11.48 | 98.33 | 0.08 | 12.71 |
| | | 4 | 96.25 | 0.97 | 13.49 | 96.25 | 0.66 | 12.91 | 82.50 | 45.99 | 71.72 | 97.08 | 0.45 | 11.48 | 98.33 | 0.08 | 12.71 |
| | | 5 | 96.25 | 0.97 | 13.49 | 96.25 | 0.66 | 12.91 | 82.50 | 45.99 | 71.72 | 97.08 | 0.45 | 11.48 | 98.33 | 0.08 | 12.71 |

| | | | | | | | | | | | | | | | | | |
|---|---|---|---|---|---|---|---|---|---|---|---|---|---|---|---|---|---|
| | | 6 | 96.25 | 0.97 | 13.49 | 96.25 | 0.66 | 12.91 | 82.50 | 45.99 | 71.72 | 97.08 | 0.45 | 11.48 | 98.33 | 0.08 | 12.71 |
| Smalt | 60 | 3 | 96.67 | 0.03 | 12.95 | 96.67 | 0.04 | 12.75 | 97.50 | 0.00 | 10.88 | 97.92 | 0.00 | 11.34 | 97.92 | 0.01 | 13.13 |
| | | 4 | 96.67 | 0.03 | 12.95 | 96.67 | 0.04 | 12.75 | 97.50 | 0.00 | 10.88 | 97.92 | 0.00 | 11.34 | 97.92 | 0.01 | 13.13 |
| | | 5 | 96.67 | 0.03 | 12.95 | 96.67 | 0.04 | 12.75 | 97.50 | 0.00 | 10.88 | 97.92 | 0.00 | 11.34 | 97.92 | 0.01 | 13.13 |
| | | 6 | 96.67 | 0.03 | 12.95 | 96.67 | 0.04 | 12.75 | 97.50 | 0.00 | 10.88 | 97.92 | 0.00 | 11.34 | 97.92 | 0.01 | 13.13 |
| | 50 | 3 | 96.67 | 0.05 | 12.94 | 97.08 | 0.05 | 12.51 | 97.92 | 0.01 | 11.36 | 97.92 | 0.01 | 11.34 | 97.92 | 0.05 | 12.87 |
| | | 4 | 96.67 | 0.05 | 12.94 | 97.08 | 0.05 | 12.51 | 97.92 | 0.01 | 11.36 | 97.92 | 0.01 | 11.34 | 97.92 | 0.05 | 12.87 |
| | | 5 | 96.67 | 0.05 | 12.94 | 97.08 | 0.05 | 12.51 | 97.92 | 0.01 | 11.36 | 97.92 | 0.01 | 11.34 | 97.92 | 0.05 | 12.87 |
| | | 6 | 96.67 | 0.05 | 12.94 | 97.08 | 0.05 | 12.51 | 97.92 | 0.01 | 11.36 | 97.92 | 0.01 | 11.34 | 97.92 | 0.05 | 12.87 |
| Stampy | 60 | 3 | 95.83 | 0.00 | 13.29 | 95.42 | 0.00 | 13.10 | 97.92 | 0.00 | 11.21 | 97.50 | 0.01 | 11.73 | 98.33 | 0.05 | 13.15 |
| | | 4 | 95.83 | 0.00 | 13.29 | 95.42 | 0.00 | 13.10 | 97.92 | 0.00 | 11.21 | 97.50 | 0.01 | 11.73 | 98.33 | 0.05 | 13.15 |
| | | 5 | 95.83 | 0.00 | 13.29 | 95.42 | 0.00 | 13.10 | 97.92 | 0.00 | 11.21 | 97.50 | 0.01 | 11.73 | 98.33 | 0.05 | 13.15 |
| | | 6 | 95.83 | 0.00 | 13.29 | 95.42 | 0.00 | 13.10 | 97.92 | 0.00 | 11.21 | 97.50 | 0.01 | 11.73 | 98.33 | 0.05 | 13.15 |
| | 50 | 3 | 95.83 | 0.00 | 13.10 | 96.25 | 0.00 | 12.97 | 98.33 | 0.01 | 11.45 | 97.50 | 0.01 | 11.72 | 98.33 | 0.08 | 13.02 |
| | | 4 | 95.83 | 0.00 | 13.10 | 96.25 | 0.00 | 12.97 | 98.33 | 0.01 | 11.45 | 97.50 | 0.01 | 11.72 | 98.33 | 0.08 | 13.02 |
| | | 5 | 95.83 | 0.00 | 13.10 | 96.25 | 0.00 | 12.97 | 98.33 | 0.01 | 11.45 | 97.50 | 0.01 | 11.72 | 98.33 | 0.08 | 13.02 |
| | | 6 | 95.83 | 0.00 | 13.10 | 96.25 | 0.00 | 12.97 | 98.33 | 0.01 | 11.45 | 97.50 | 0.01 | 11.72 | 98.33 | 0.08 | 13.02 |

E

| **1.50% (50kb-1mb)** | | | **36** | | | **50** | | | **76** | | | **100** | | | **150** | | |
|---|---|---|---|---|---|---|---|---|---|---|---|---|---|---|---|---|---|
| **Aligner** | **Bin size** | **Step size** | **TP** | **FP** | **SD** | **TP** | **FP** | **SD** | **TP** | **FP** | **SD** | **TP** | **FP** | **SD** | **TP** | **FP** | **SD** |
| Bfast | 60 | 3 | 100.00 | 0.00 | 0.51 | 100.00 | 0.01 | 0.36 | 100.00 | 0.01 | 0.72 | 100.00 | 0.01 | 1.44 | 100.00 | 0.04 | 2.35 |
| | | 4 | 100.00 | 0.00 | 0.51 | 100.00 | 0.01 | 0.36 | 100.00 | 0.01 | 0.72 | 100.00 | 0.01 | 1.44 | 100.00 | 0.04 | 2.35 |
| | | 5 | 100.00 | 0.00 | 0.51 | 100.00 | 0.01 | 0.36 | 100.00 | 0.01 | 0.72 | 100.00 | 0.01 | 1.44 | 100.00 | 0.04 | 2.35 |
| | | 6 | 100.00 | 0.00 | 0.51 | 100.00 | 0.01 | 0.36 | 100.00 | 0.01 | 0.72 | 100.00 | 0.01 | 1.44 | 100.00 | 0.04 | 2.35 |
| | 50 | 3 | 100.00 | 0.01 | 0.50 | 100.00 | 0.01 | 0.38 | 100.00 | 0.01 | 0.87 | 100.00 | 0.03 | 1.61 | 100.00 | 0.09 | 2.55 |
| | | 4 | 100.00 | 0.01 | 0.50 | 100.00 | 0.01 | 0.38 | 100.00 | 0.01 | 0.87 | 100.00 | 0.03 | 1.61 | 100.00 | 0.09 | 2.55 |
| | | 5 | 100.00 | 0.01 | 0.50 | 100.00 | 0.01 | 0.38 | 100.00 | 0.01 | 0.87 | 100.00 | 0.03 | 1.61 | 100.00 | 0.09 | 2.55 |
| | | 6 | 100.00 | 0.01 | 0.50 | 100.00 | 0.01 | 0.38 | 100.00 | 0.01 | 0.87 | 100.00 | 0.03 | 1.61 | 100.00 | 0.09 | 2.55 |
| BWA | 60 | 3 | 100.00 | 0.01 | 0.66 | 100.00 | 0.00 | 0.36 | 100.00 | 0.00 | 0.72 | 100.00 | 0.00 | 1.41 | 100.00 | 0.01 | 2.26 |
| | | 4 | 100.00 | 0.01 | 0.66 | 100.00 | 0.00 | 0.36 | 100.00 | 0.00 | 0.72 | 100.00 | 0.00 | 1.41 | 100.00 | 0.01 | 2.26 |
| | | 5 | 100.00 | 0.01 | 0.66 | 100.00 | 0.00 | 0.36 | 100.00 | 0.00 | 0.72 | 100.00 | 0.00 | 1.41 | 100.00 | 0.01 | 2.26 |

| | | | | | | | | | | | | | | | | | |
|---|---|---|---|---|---|---|---|---|---|---|---|---|---|---|---|---|---|
| | | 6 | 100.00 | 0.01 | 0.66 | 100.00 | 0.00 | 0.36 | 100.00 | 0.00 | 0.72 | 100.00 | 0.00 | 1.41 | 100.00 | 0.01 | 2.26 |
| | 50 | 3 | 100.00 | 0.02 | 0.72 | 100.00 | 0.00 | 0.38 | 100.00 | 0.00 | 0.87 | 100.00 | 0.00 | 1.58 | 100.00 | 0.05 | 2.51 |
| | | 4 | 100.00 | 0.02 | 0.72 | 100.00 | 0.00 | 0.38 | 100.00 | 0.00 | 0.87 | 100.00 | 0.00 | 1.58 | 100.00 | 0.05 | 2.51 |
| | | 5 | 100.00 | 0.02 | 0.72 | 100.00 | 0.00 | 0.38 | 100.00 | 0.00 | 0.87 | 100.00 | 0.00 | 1.58 | 100.00 | 0.05 | 2.51 |
| | | 6 | 100.00 | 0.02 | 0.72 | 100.00 | 0.00 | 0.38 | 100.00 | 0.00 | 0.87 | 100.00 | 0.00 | 1.58 | 100.00 | 0.05 | 2.51 |
| Novo | 60 | 3 | 100.00 | 0.00 | 0.15 | 100.00 | 0.00 | 0.28 | 100.00 | 0.00 | 0.11 | 100.00 | 0.01 | 1.36 | 100.00 | 0.04 | 2.28 |
| | | 4 | 100.00 | 0.00 | 0.15 | 100.00 | 0.00 | 0.28 | 100.00 | 0.00 | 0.11 | 100.00 | 0.01 | 1.36 | 100.00 | 0.04 | 2.28 |
| | | 5 | 100.00 | 0.00 | 0.15 | 100.00 | 0.00 | 0.28 | 100.00 | 0.00 | 0.11 | 100.00 | 0.01 | 1.36 | 100.00 | 0.04 | 2.28 |
| | | 6 | 100.00 | 0.00 | 0.15 | 100.00 | 0.00 | 0.28 | 100.00 | 0.00 | 0.11 | 100.00 | 0.01 | 1.36 | 100.00 | 0.04 | 2.28 |
| | 50 | 3 | 100.00 | 0.00 | 0.20 | 100.00 | 0.01 | 0.26 | 100.00 | 0.00 | 0.13 | 100.00 | 0.01 | 1.54 | 100.00 | 0.09 | 2.48 |
| | | 4 | 100.00 | 0.00 | 0.20 | 100.00 | 0.01 | 0.26 | 100.00 | 0.00 | 0.13 | 100.00 | 0.01 | 1.54 | 100.00 | 0.09 | 2.48 |
| | | 5 | 100.00 | 0.00 | 0.20 | 100.00 | 0.01 | 0.26 | 100.00 | 0.00 | 0.13 | 100.00 | 0.01 | 1.54 | 100.00 | 0.09 | 2.48 |
| | | 6 | 100.00 | 0.00 | 0.20 | 100.00 | 0.01 | 0.26 | 100.00 | 0.00 | 0.13 | 100.00 | 0.01 | 1.54 | 100.00 | 0.09 | 2.48 |
| Smalt | 60 | 3 | 100.00 | 0.02 | 0.37 | 100.00 | 0.01 | 0.72 | 100.00 | 0.00 | 0.76 | 100.00 | 0.00 | 1.98 | 100.00 | 0.01 | 2.35 |
| | | 4 | 100.00 | 0.02 | 0.37 | 100.00 | 0.01 | 0.72 | 100.00 | 0.00 | 0.76 | 100.00 | 0.00 | 1.98 | 100.00 | 0.01 | 2.35 |
| | | 5 | 100.00 | 0.02 | 0.37 | 100.00 | 0.01 | 0.72 | 100.00 | 0.00 | 0.76 | 100.00 | 0.00 | 1.98 | 100.00 | 0.01 | 2.35 |
| | | 6 | 100.00 | 0.02 | 0.37 | 100.00 | 0.01 | 0.72 | 100.00 | 0.00 | 0.76 | 100.00 | 0.00 | 1.98 | 100.00 | 0.01 | 2.35 |
| | 50 | 3 | 100.00 | 0.03 | 0.38 | 100.00 | 0.02 | 0.75 | 100.00 | 0.00 | 0.94 | 100.00 | 0.01 | 2.08 | 100.00 | 0.05 | 2.53 |
| | | 4 | 100.00 | 0.03 | 0.38 | 100.00 | 0.02 | 0.75 | 100.00 | 0.00 | 0.94 | 100.00 | 0.01 | 2.08 | 100.00 | 0.05 | 2.53 |
| | | 5 | 100.00 | 0.03 | 0.38 | 100.00 | 0.02 | 0.75 | 100.00 | 0.00 | 0.94 | 100.00 | 0.01 | 2.08 | 100.00 | 0.05 | 2.53 |
| | | 6 | 100.00 | 0.03 | 0.38 | 100.00 | 0.02 | 0.75 | 100.00 | 0.00 | 0.94 | 100.00 | 0.01 | 2.08 | 100.00 | 0.05 | 2.53 |
| Stampy | 60 | 3 | 100.00 | 0.00 | 0.56 | 100.00 | 0.00 | 0.35 | 100.00 | 0.00 | 0.76 | 100.00 | 0.01 | 1.42 | 100.00 | 0.01 | 2.38 |
| | | 4 | 100.00 | 0.00 | 0.56 | 100.00 | 0.00 | 0.35 | 100.00 | 0.00 | 0.76 | 100.00 | 0.01 | 1.42 | 100.00 | 0.01 | 2.38 |
| | | 5 | 100.00 | 0.00 | 0.56 | 100.00 | 0.00 | 0.35 | 100.00 | 0.00 | 0.76 | 100.00 | 0.01 | 1.42 | 100.00 | 0.01 | 2.38 |
| | | 6 | 100.00 | 0.00 | 0.56 | 100.00 | 0.00 | 0.35 | 100.00 | 0.00 | 0.76 | 100.00 | 0.01 | 1.42 | 100.00 | 0.01 | 2.38 |
| | 50 | 3 | 100.00 | 0.00 | 0.58 | 100.00 | 0.00 | 0.36 | 100.00 | 0.00 | 0.96 | 100.00 | 0.01 | 1.59 | 100.00 | 0.04 | 2.62 |
| | | 4 | 100.00 | 0.00 | 0.58 | 100.00 | 0.00 | 0.36 | 100.00 | 0.00 | 0.96 | 100.00 | 0.01 | 1.59 | 100.00 | 0.04 | 2.62 |
| | | 5 | 100.00 | 0.00 | 0.58 | 100.00 | 0.00 | 0.36 | 100.00 | 0.00 | 0.96 | 100.00 | 0.01 | 1.59 | 100.00 | 0.04 | 2.62 |
| | | 6 | 100.00 | 0.00 | 0.58 | 100.00 | 0.00 | 0.36 | 100.00 | 0.00 | 0.96 | 100.00 | 0.01 | 1.59 | 100.00 | 0.04 | 2.62 |

F

| **3.00% (1-5mb)** | | | **36** | | | **50** | | | **76** | | | **100** | | | **150** | | |
|---|---|---|---|---|---|---|---|---|---|---|---|---|---|---|---|---|---|
| **Aligner** | **Bin size** | **Step size** | **TP** | **FP** | **SD** | **TP** | **FP** | **SD** | **TP** | **FP** | **SD** | **TP** | **FP** | **SD** | **TP** | **FP** | **SD** |
| Bfast | 60 | 3 | 100.00 | 0.00 | 0.33 | 100.00 | 0.00 | 0.32 | 100.00 | 0.01 | 0.39 | 100.00 | 0.58 | 0.98 | 100.00 | 0.05 | 2.22 |
| | | 4 | 100.00 | 0.00 | 0.33 | 100.00 | 0.00 | 0.32 | 100.00 | 0.01 | 0.39 | 100.00 | 0.58 | 0.98 | 100.00 | 0.05 | 2.22 |
| | | 5 | 100.00 | 0.00 | 0.33 | 100.00 | 0.00 | 0.32 | 100.00 | 0.01 | 0.39 | 100.00 | 0.58 | 0.98 | 100.00 | 0.05 | 2.22 |
| | | 6 | 100.00 | 0.00 | 0.33 | 100.00 | 0.00 | 0.32 | 100.00 | 0.01 | 0.39 | 100.00 | 0.58 | 0.98 | 100.00 | 0.05 | 2.72 |
| | 50 | 3 | 100.00 | 0.00 | 0.35 | 100.00 | 0.01 | 0.34 | 100.00 | 0.01 | 0.49 | 100.00 | 0.61 | 1.10 | 100.00 | 0.11 | 2.43 |
| | | 4 | 100.00 | 0.00 | 0.35 | 100.00 | 0.01 | 0.34 | 100.00 | 0.01 | 0.49 | 100.00 | 0.61 | 1.10 | 100.00 | 0.11 | 2.43 |
| | | 5 | 100.00 | 0.00 | 0.35 | 100.00 | 0.01 | 0.34 | 100.00 | 0.01 | 0.49 | 100.00 | 0.61 | 1.10 | 100.00 | 0.11 | 2.43 |
| | | 6 | 100.00 | 0.00 | 0.35 | 100.00 | 0.01 | 0.34 | 100.00 | 0.01 | 0.49 | 100.00 | 0.61 | 1.10 | 100.00 | 0.11 | 2.43 |
| BWA | 60 | 3 | 100.00 | 0.01 | 0.71 | 100.00 | 0.00 | 0.34 | 100.00 | 0.00 | 0.46 | 100.00 | 0.00 | 0.89 | 100.00 | 0.02 | 2.19 |
| | | 4 | 100.00 | 0.01 | 0.71 | 100.00 | 0.00 | 0.34 | 100.00 | 0.00 | 0.46 | 100.00 | 0.00 | 0.89 | 100.00 | 0.02 | 2.19 |
| | | 5 | 100.00 | 0.01 | 0.71 | 100.00 | 0.00 | 0.34 | 100.00 | 0.00 | 0.46 | 100.00 | 0.00 | 0.89 | 100.00 | 0.02 | 2.19 |
| | | 6 | 100.00 | 0.01 | 0.71 | 100.00 | 0.00 | 0.34 | 100.00 | 0.00 | 0.46 | 100.00 | 0.00 | 0.89 | 100.00 | 0.02 | 2.19 |
| | 50 | 3 | 100.00 | 0.03 | 0.80 | 100.00 | 0.00 | 0.36 | 100.00 | 0.00 | 0.55 | 100.00 | 0.00 | 1.04 | 100.00 | 0.07 | 2.39 |
| | | 4 | 100.00 | 0.03 | 0.80 | 100.00 | 0.00 | 0.36 | 100.00 | 0.00 | 0.55 | 100.00 | 0.00 | 1.04 | 100.00 | 0.07 | 2.39 |
| | | 5 | 100.00 | 0.03 | 0.80 | 100.00 | 0.00 | 0.36 | 100.00 | 0.00 | 0.55 | 100.00 | 0.00 | 1.04 | 100.00 | 0.07 | 2.39 |
| | | 6 | 100.00 | 0.03 | 0.80 | 100.00 | 0.00 | 0.36 | 100.00 | 0.00 | 0.55 | 100.00 | 0.00 | 1.04 | 100.00 | 0.07 | 2.39 |
| Novo | 60 | 3 | 100.00 | 0.00 | 0.09 | 100.00 | 0.00 | 0.19 | 100.00 | 0.01 | 0.39 | 100.00 | 0.01 | 0.88 | 100.00 | 0.05 | 2.16 |
| | | 4 | 100.00 | 0.00 | 0.09 | 100.00 | 0.00 | 0.19 | 100.00 | 0.01 | 0.39 | 100.00 | 0.01 | 0.88 | 100.00 | 0.05 | 2.16 |
| | | 5 | 100.00 | 0.00 | 0.09 | 100.00 | 0.00 | 0.19 | 100.00 | 0.01 | 0.39 | 100.00 | 0.01 | 0.88 | 100.00 | 0.05 | 2.16 |
| | | 6 | 100.00 | 0.00 | 0.09 | 100.00 | 0.00 | 0.19 | 100.00 | 0.01 | 0.39 | 100.00 | 0.01 | 0.88 | 100.00 | 0.05 | 2.16 |
| | 50 | 3 | 100.00 | 0.00 | 0.10 | 100.00 | 0.00 | 0.20 | 100.00 | 0.01 | 0.49 | 100.00 | 0.02 | 1.00 | 100.00 | 0.11 | 2.35 |
| | | 4 | 100.00 | 0.00 | 0.10 | 100.00 | 0.00 | 0.20 | 100.00 | 0.01 | 0.49 | 100.00 | 0.02 | 1.00 | 100.00 | 0.11 | 2.35 |
| | | 5 | 100.00 | 0.00 | 0.10 | 100.00 | 0.00 | 0.20 | 100.00 | 0.01 | 0.49 | 100.00 | 0.02 | 1.00 | 100.00 | 0.11 | 2.35 |
| | | 6 | 100.00 | 0.00 | 0.10 | 100.00 | 0.00 | 0.20 | 100.00 | 0.01 | 0.49 | 100.00 | 0.02 | 1.00 | 100.00 | 0.11 | 2.35 |
| Smalt | 60 | 3 | 100.00 | 0.02 | 0.37 | 100.00 | 0.01 | 0.71 | 100.00 | 0.00 | 0.59 | 100.00 | 0.00 | 1.34 | 100.00 | 0.02 | 2.26 |
| | | 4 | 100.00 | 0.02 | 0.37 | 100.00 | 0.01 | 0.71 | 100.00 | 0.00 | 0.59 | 100.00 | 0.00 | 1.34 | 100.00 | 0.02 | 2.26 |
| | | 5 | 100.00 | 0.02 | 0.37 | 100.00 | 0.01 | 0.71 | 100.00 | 0.00 | 0.59 | 100.00 | 0.00 | 1.34 | 100.00 | 0.02 | 2.26 |
| | | 6 | 100.00 | 0.02 | 0.37 | 100.00 | 0.01 | 0.71 | 100.00 | 0.00 | 0.59 | 100.00 | 0.00 | 1.34 | 100.00 | 0.02 | 2.26 |
| | 50 | 3 | 100.00 | 0.03 | 0.40 | 100.00 | 0.01 | 0.85 | 100.00 | 0.00 | 0.70 | 100.00 | 0.01 | 1.59 | 100.00 | 0.08 | 2.51 |
| | | 4 | 100.00 | 0.03 | 0.40 | 100.00 | 0.01 | 0.85 | 100.00 | 0.00 | 0.70 | 100.00 | 0.01 | 1.59 | 100.00 | 0.08 | 2.51 |

| | | | | | | | | | | | | | | | | | |
|---|---|---|---|---|---|---|---|---|---|---|---|---|---|---|---|---|---|
| | | 5 | 100.00 | 0.03 | 0.40 | 100.00 | 0.01 | 0.85 | 100.00 | 0.00 | 0.70 | 100.00 | 0.01 | 1.59 | 100.00 | 0.08 | 2.51 |
| | | 6 | 100.00 | 0.03 | 0.40 | 100.00 | 0.01 | 0.85 | 100.00 | 0.00 | 0.70 | 100.00 | 0.01 | 1.59 | 100.00 | 0.08 | 2.51 |
| Stampy | 60 | 3 | 100.00 | 0.00 | 0.46 | 100.00 | 0.00 | 0.33 | 100.00 | 0.00 | 0.47 | 100.00 | 0.00 | 0.88 | 100.00 | 0.01 | 3.91 |
| | | 4 | 100.00 | 0.00 | 0.46 | 100.00 | 0.00 | 0.33 | 100.00 | 0.00 | 0.47 | 100.00 | 0.00 | 0.88 | 100.00 | 0.01 | 3.17 |
| | | 5 | 100.00 | 0.00 | 0.46 | 100.00 | 0.00 | 0.33 | 100.00 | 0.00 | 0.47 | 100.00 | 0.00 | 0.88 | 100.00 | 0.02 | 2.64 |
| | | 6 | 100.00 | 0.00 | 0.46 | 100.00 | 0.00 | 0.33 | 100.00 | 0.00 | 0.47 | 100.00 | 0.00 | 0.88 | 100.00 | 0.02 | 2.28 |
| | 50 | 3 | 100.00 | 0.00 | 0.49 | 100.00 | 0.00 | 0.37 | 100.00 | 0.00 | 0.56 | 100.00 | 0.00 | 1.03 | 100.00 | 0.03 | 3.78 |
| | | 4 | 100.00 | 0.00 | 0.49 | 100.00 | 0.00 | 0.37 | 100.00 | 0.00 | 0.56 | 100.00 | 0.00 | 1.03 | 100.00 | 0.04 | 3.18 |
| | | 5 | 100.00 | 0.00 | 0.49 | 100.00 | 0.00 | 0.37 | 100.00 | 0.00 | 0.56 | 100.00 | 0.00 | 1.03 | 100.00 | 0.05 | 2.82 |
| | | 6 | 100.00 | 0.00 | 0.49 | 100.00 | 0.00 | 0.37 | 100.00 | 0.00 | 0.56 | 100.00 | 0.00 | 1.03 | 100.00 | 0.06 | 2.40 |

COPS found SCNAs with varying numbers of true positives, TP, false positives, FP and size deviation, SD, when tried with simulated data of varying read lengths, 36, 50, 76, 100 and 150 at two bin sizes, 50 and 60, and four step sizes for smoothening $log_2$ratios, 3, 4, 5 and 6, respectively. The simulated data covered 0.05% (A) , 0.10% (B), 1.50% (C-E) and 3% (F) of chr1 with the indicated size ranges of simulated SCNAs, with an average coverage of 15X paired-end reads aligned using five different tools, Bfast, BWA, Novo, Smalt and Stampy.
