# Supplementary material for: COPS: A Sensitive and Accurate Tool for Detecting Somatic Copy Number Alterations Using Short-Read Sequence Data from Paired Samples
Source: PLoS One. 2012 Oct 22;7(10):e47812. doi: 10.1371/journal.pone.0047812 (PMC3478291; doi:10.1371/journal.pone.0047812)
Supplement: Table S2 — Chromosome-wise validation of SCNAs detected by COPS using whole genome SNP microarray. (PDF) [file pone.0047812.s009.pdf]

Supporting Table 2. Chromosome-wise validation of SCNAs detected by COPS using whole genome SNP microarray.

| Chr | CNA sizes from sequencing reads using COPS | CNA sizes found by using whole genome SNP microarray | % Overlap after adjusting coverage >= 1.5X | % Overlap after adjusting coverage >= 2.5X | % Overlap after adjusting coverage >= 5X | % Overlap after adjusting coverage >= 7.5X | % Overlap after adjusting coverage >= 10X | % Overlap after adjusting coverage >= 15X |
|---|---|---|---|---|---|---|---|---|
| 1 | 39456276 | 10773720 | 87.43 | 93.97 | 98.99 | 99.84 | 99.97 | 100.00 |
| 2 | 55481916 | 23368947 | 87.19 | 93.73 | 98.92 | 99.81 | 99.97 | 100.00 |
| 3 | 32994154 | 198544 | 68.59 | 78.09 | 89.17 | 93.75 | 96.12 | 97.81 |
| 4 | 123756785 | 112241762 | 87.68 | 93.88 | 98.83 | 99.70 | 99.87 | 99.91 |
| 5 | 36875208 | 8142814 | 89.63 | 94.94 | 99.09 | 99.83 | 99.96 | 99.99 |
| 6 | 25221070 | 835279 | 70.19 | 79.35 | 91.91 | 96.92 | 98.97 | 99.89 |
| 7 | 2973684 | 8751693 | 84.33 | 92.36 | 98.64 | 99.74 | 99.93 | 99.98 |
| 8 | 20361798 | 388438 | 83.51 | 90.70 | 97.11 | 98.84 | 99.43 | 99.81 |
| 9 | 28267798 | 22242420 | 90.67 | 95.65 | 99.29 | 99.81 | 99.89 | 99.93 |
| 10 | 52298975 | 34672569 | 88.25 | 94.07 | 98.79 | 99.64 | 99.80 | 99.87 |
| 11 | 30253616 | 6978704 | 75.56 | 86.68 | 97.05 | 99.38 | 99.87 | 99.99 |
| 12 | 19433592 | 8511 | 89.27 | 94.13 | 99.41 | 100.00 | 100.00 | 100.00 |
| 13 | 15064650 | NA* | NA | NA | NA | NA | NA | NA |
| 14 | 14471659 | 745161 | 88.88 | 93.59 | 98.26 | 99.58 | 99.88 | 99.97 |
| 15 | 30176985 | 15184678 | 88.70 | 94.53 | 99.07 | 99.85 | 99.98 | 100.00 |
| 16 | 12237383 | 177784 | 61.05 | 71.88 | 85.57 | 91.37 | 94.32 | 97.19 |
| 17 | 23814203 | 15674745 | 87.48 | 93.98 | 99.00 | 99.83 | 99.97 | 100.00 |
| 18 | 11703900 | NA | NA | NA | NA | NA | NA | NA |
| 19 | 13122559 | 3015860 | 83.03 | 91.30 | 98.27 | 99.55 | 99.87 | 99.97 |
| 20 | 21331257 | 14209770 | 85.15 | 92.56 | 98.63 | 99.75 | 99.96 | 100.00 |
| 21 | 5182301 | 66771 | 9.81 | 14.23 | 26.13 | 37.11 | 45.50 | 61.81 |
| 22 | 5395036 | NA | NA | NA | NA | NA | NA | NA |
| X | 3811262 | 20231 | 93.60 | 96.79 | 98.76 | 99.51 | 99.75 | 100.00 |
| Y | 1074159 | 470352 | 97.01 | 98.75 | 99.84 | 99.99 | 100.00 | 100.00 |
| All | 651523026 | 278168753 | 80.81 | 87.39 | 93.85 | 95.90 | 96.81 | 97.91 |

*Not Applicable

Overlapping loci involved in SCNA events were estimated using COPS on tumor:normal mate-pair sequencing data and CNVPartitition2.4.4 plugin in GenomeStudio on Omni2.5M SNP array data for the same samples. The overlap was also estimated after systematically omitting reads not satisfying sequencing coverage thresholds of 1.5X, 2.5X, 5X, 7.5X, 10X and 15X, respectively, for either normal or tumor samples.
