# Supplementary material for: COPS: A Sensitive and Accurate Tool for Detecting Somatic Copy Number Alterations Using Short-Read Sequence Data from Paired Samples
Source: PLoS One. 2012 Oct 22;7(10):e47812. doi: 10.1371/journal.pone.0047812 (PMC3478291; doi:10.1371/journal.pone.0047812)
Supplement: Table S3 — Number of SCNAs in each simulated dataset. (PDF) [file pone.0047812.s010.pdf]

Supporting Table 3 Number of SCNAs in each simulated dataset.

The % indicates the %coverage of SCNAs in chr1. The size range of the simulated SCNAs is indicated within parantheses.

**Data simulations**	**No. of SCNAs**
0.05% (1-10kb)	34 [32^{D};2^{A}]
0.10% (10-50 kb)	15 [11^{D};4^{A}]
1.50% (1-10kb)	1247 [1094^{D};153^{A}]
1.50% (10-50kb)	240 [195^{D};45^{A}]
1.50% (50kb-1mb)	13 [11^{D};2^{A}]
3.00% (1-5mb)	20 [13^{D};7^{A}]

^{A}amplification; ^{D}deletion
