# Supplementary material for: COPS: A Sensitive and Accurate Tool for Detecting Somatic Copy Number Alterations Using Short-Read Sequence Data from Paired Samples
Source: PLoS One. 2012 Oct 22;7(10):e47812. doi: 10.1371/journal.pone.0047812 (PMC3478291; doi:10.1371/journal.pone.0047812)
Supplement: Text S1 — Commands used to run various CNA/CNV detection tools for performance comparison with COPS. (PDF) [file pone.0047812.s011.pdf]

Supporting Text 1

Commands used to run various CNA/CNV detection tools for performance comparison with COPS:

1. CNVNator
   /Apps/serial/CNVnator/cnvnator -chrom chr1 -root stampy--chr1_0.05_1000_10000_36_A12_sorted.bam.root -tree stampy--chr1_0.05_1000_10000_36_A12_sorted.bam

   /Apps/serial/CNVnator/cnvnator -chrom chr1 -root stampy--chr1_0.05_1000_10000_36_A12_sorted.bam.root -his 100 -d /common/Data/Internal/Others/Reference_genome/Hu_ref/

   /Apps/serial/CNVnator/cnvnator -chrom chr1 -root stampy--chr1_0.05_1000_10000_36_A12_sorted.bam.root -stat 100

   /Apps/serial/CNVnator/cnvnator -chrom chr1 -root stampy--chr1_0.05_1000_10000_36_A12_sorted.bam.root -partition 100

   /Apps/serial/CNVnator/cnvnator -chrom chr1 -root stampy--chr1_0.05_1000_10000_36_A12_sorted.bam.root -call 100 > stampy--chr1_0.05_1000_10000_36_A12_sorted.bam.cnv_calls.out

2. RDXplorer
   python rdxplorer.py

   Parameters set in run.sh:

   path2bam= 'stampy--chr1_ 0.05_1000_10000_36_A12_sorted.bam'
   reference= 'chr1.fa'
   wrkgdir='stampy_0.05_36_rdxOutput'

   chromOfInterest='1'
   hg= 'hg18'

3. Freec

mkdir freec_out-stampy

samtools view stampy--chr1_0.05_1000_10000_36_A12_sorted.bam > stampy--chr1_0.05_1000_10000_36_A12_sorted.bam.sam

./freec -conf stampy-config.txt

stampy-config.txt:

```
chrLenFile = chr1.len

ploidy = 2

breakPointThreshold = -.0001

window = 1000

chrFiles = /common/Data/Internal/Others/Reference_genome/Hu_ref/


#numberOfProcesses = 2

#coefficientOfVariation = 0.01

#GCcontentProfile = test/GC_profile_50kb.cnp

outputDir = freec_out-stampy/

#contaminationAdjustment = TRUE

step=500

#minMappabilityPerWindow = 0.95

#gemMappabilityFile = /GEM_mappability/out76.gem

#minExpectedGC=0.35

#maxExpectedGC=0.55


[sample]


mateFile = stampy--chr1_0.05_1000_10000_36_A12_sorted.bam.sam


#mateCopyNumberFile = test/sample.cpn

inputFormat = SAM

mateOrientation = FR


[control]
```

#mateFile = /path/control.sam

#mateCopyNumberFile = path/control.cpn

#inputFormat = SAM

#mateOrientation = FR

4. CNV-Seq

samtools view bwa--chr1_0.05_1000_10000_36_A12_sorted.bam | perl -lane 'print $F[2]\t$F[3]' > bwa--chr1_0.05_1000_10000_36_A12_sorted.bam.hits

/Apps/serial/cnv-seq/cnv-seq.pl --test bwa--chr1_0.05_1000_10000_36_A12_sorted.bam.hits --ref /ref/36/bwa--ref_chr1_36_sorted.hits --genome chrom1

R CMD BATCH cnv_seg.R > bwa—chr1_0.05_1000_10000_36_A12_sorted.bam.cnv-seg.txt

cnv_seg.R:

library(cnv)

ta <- read.delim(bwa--chr1_0.05_1000_10000_36_A12_sorted.bam.hits-vs-novo--ref_chr1_36_sorted.hits.log2-0.6.pvalue-0.001.minw-4.cnv)

cnv.print(ta)

5. SVDetect

/SVDetect_r0.7f/bin/SVDetect cnv -conf stampy-sample.cnv.conf

stampy-sample.cnv.conf
:

<general>

input_format = bam

sv_type = all

mates_orientation=FR

output_dir=stampy-result_200base-bin

</general>

<detection>

tag_lenght=150

read1_length=150

read2_length=150

window_size=155

step_length=80

mates_file=stampy--chr1_0.05_1000_10000_36_A12_sorted.bam

mates_file_ref=/ref/36/stampy--ref_chr1_36_sorted.bam

cmap_file=/SVDetect_r0.7f/chr1.len

</detection>
