# Supplementary material for: COPS: A Sensitive and Accurate Tool for Detecting Somatic Copy Number Alterations Using Short-Read Sequence Data from Paired Samples
Source: PLoS One. 2012 Oct 22;7(10):e47812. doi: 10.1371/journal.pone.0047812 (PMC3478291; doi:10.1371/journal.pone.0047812)
Supplement: Text S2 — The R codes and a sample input file used to generate Figure 7 and Figure S3. (PDF) [file pone.0047812.s012.pdf]

Supplementary Text 2

In Figure 7 and Supporting Figure 4, the size of a data point is representative of the size deviation (SD) of the detected SCNA. The SD value is scaled down by a factor of 10, before being passed on to the various shapes in the symbols function. The radius of the circle, side of the square, base of the triangle, edge of the star and height of the rectangle are comparable to each other. The size deviation can thus be compared across shapes of datapoints, representative of various aligners being used. The R codes and a sample input file used to generate Figure 7 and Supporting Figure 3 are provided below.

Sample input file (Bfast_36_0.05):-

```
COPS  100     0.1     6.85

RDXplorer     97.06   0.26    11.56

CNVNator      88.24   0.21    12.37

Freec   0     0.48    100

SVDetect      100     119.19 64.21
```

R code to generate Figure 7:-

```
Bfast76_15_1_1=read.table("Bfast_76_1.50_1_1")

BWA76_15_1_1=read.table("BWA_76_1.50_1_1")

Smalt76_15_1_1=read.table("Smalt_76_1.50_1_1")

Stampy76_15_1_1=read.table("Stampy_76_1.50_1_1")

Novo76_15_1_1=read.table("Novo_76_1.50_1_1")



#par(new=TRUE)



par(mar=c(5,2,1,1),mfrow=c(2,3))

N <- nrow(Bfast76_15_1_1)

   with(Bfast76_15_1_1, {

         palette(rainbow(N, end = 0.8))

         symbols(Bfast76_15_1_1$V3, Bfast76_15_1_1$V2, circles=Bfast76_15_1_1$V4/20,
inches=FALSE, fg = 1:N, bg="transparent", main="A
",font.main=2,cex.main=1,just="left",xlab="",ylab="",xlim=c(-10,180),ylim=c(0,130))

         symbols(BWA76_15_1_1$V3, BWA76_15_1_1$V2, squares=BWA76_15_1_1$V4/10,
add=TRUE, inches=FALSE, fg = 1:N, bg="transparent")
```

```
symbols(Smalt76_15_1_1$V3, Smalt76_15_1_1$V2,
rectangles=cbind(Smalt76_15_1_1$V4/15,Smalt76_15_1_1$V4/10), add=TRUE, inches=FALSE,
fg = 1:N, bg="transparent")

symbols(Stampy76_15_1_1$V3, Stampy76_15_1_1$V2,
stars=cbind(Stampy76_15_1_1$V4/5.773502692,Stampy76_15_1_1$V4/5.773502692,Stampy76_1
5_1_1$V4/5.773502692), add=TRUE, inches=FALSE, fg = 1:N, bg="transparent")

symbols(Novo76_15_1_1$V3, Novo76_15_1_1$V2,
stars=cbind(Novo76_15_1_1$V4/8.506508084,Novo76_15_1_1$V4/8.506508084,Novo76_15_1_1
$V4/8.506508084,Novo76_15_1_1$V4/8.506508084,Novo76_15_1_1$V4/8.506508084),
add=TRUE, inches=FALSE, fg = 1:N, bg="transparent")


palette("default")

})

Bfast76_15_10_1=read.table("Bfast_76_1.50_10_1")

BWA76_15_10_1=read.table("BWA_76_1.50_10_1")

Smalt76_15_10_1=read.table("Smalt_76_1.50_10_1")

Stampy76_15_10_1=read.table("Stampy_76_1.50_10_1")

Novo76_15_10_1=read.table("Novo_76_1.50_10_1")


N <- nrow(Bfast76_15_10_1)

with(Bfast76_15_10_1, {

palette(rainbow(N, end = 0.8))

symbols(Bfast76_15_10_1$V3, Bfast76_15_10_1$V2, circles=Bfast76_15_10_1$V4/20,
inches=FALSE, fg = 1:N, bg="transparent", main="CNA detection
tools",cex.main=1,font.main=2,xlab="",ylab="",xlim=c(-10,180),ylim=c(0,130),yaxt="n")

symbols(BWA76_15_10_1$V3, BWA76_15_10_1$V2, squares=BWA76_15_10_1$V4/10,
add=TRUE, inches=FALSE, fg = 1:N, bg="transparent")

symbols(Smalt76_15_10_1$V3, Smalt76_15_10_1$V2,
rectangles=cbind(Smalt76_15_10_1$V4/15,Smalt76_15_10_1$V4/10), add=TRUE,
inches=FALSE, fg = 1:N, bg="transparent")

symbols(Stampy76_15_10_1$V3, Stampy76_15_10_1$V2,
stars=cbind(Stampy76_15_10_1$V4/5.773502692,Stampy76_15_10_1$V4/5.773502692,Stampy76
_15_10_1$V4/5.773502692), add=TRUE, inches=FALSE, fg = 1:N, bg="transparent")
```

```
        symbols(Novo76_15_10_1$V3, Novo76_15_10_1$V2, stars=cbind(Novo76_15_10_1$V4/8.506508084,Novo76_15_10_1$V4/8.506508084,Novo76_15_10_1$V4/8.506508084,Novo76_15_10_1$V4/8.506508084,Novo76_15_10_1$V4/8.506508084), add=TRUE, inches=FALSE, fg = 1:N, bg="transparent")

      palette("default")

   })

Bfast76_15_50_1=read.table("Bfast_76_1.50_50_1")

BWA76_15_50_1=read.table("BWA_76_1.50_50_1")

Smalt76_15_50_1=read.table("Smalt_76_1.50_50_1")

Stampy76_15_50_1=read.table("Stampy_76_1.50_50_1")

Novo76_15_50_1=read.table("Novo_76_1.50_50_1")

leg.txt <- c("Bfast", "BWA", "Novo", "Smalt", "Stampy")

N <- nrow(Bfast76_15_50_1)

   with(Bfast76_15_50_1, {

        palette(rainbow(N, end = 0.8))

        symbols(Bfast76_15_50_1$V3, Bfast76_15_50_1$V2, circles=Bfast76_15_50_1$V4/20, inches=FALSE, fg = 1:N, bg="transparent", xlab="",ylab="",xlim=c(-10,180),ylim=c(0,130),yaxt="n")

        legend (134,10,Bfast76_15_50_1$V1,col=1:N,box.col="transparent",text.col="black",fill=1:N,cex=0.5)

        legend (92,21,legend=leg.txt, box.col="transparent",col="black", pch=c(1,0,39,2,11),cex=0.6)

        symbols(BWA76_15_50_1$V3, BWA76_15_50_1$V2, squares=BWA76_15_50_1$V4/10, add=TRUE, inches=FALSE, fg = 1:N, bg="transparent")

        symbols(Smalt76_15_50_1$V3, Smalt76_15_50_1$V2, rectangles=cbind(Smalt76_15_50_1$V4/15,Smalt76_15_50_1$V4/10), add=TRUE, inches=FALSE, fg = 1:N, bg="transparent")

        symbols(Stampy76_15_50_1$V3, Stampy76_15_50_1$V2, stars=cbind(Stampy76_15_50_1$V4/5.773502692,Stampy76_15_50_1$V4/5.773502692,Stampy76_15_50_1$V4/5.773502692), add=TRUE, inches=FALSE, fg = 1:N, bg="transparent")
```

```
symbols(Novo76_15_50_1$V3, Novo76_15_50_1$V2, stars=cbind(Novo76_15_50_1$V4/8.506508084,Novo76_15_50_1$V4/8.506508084,Novo76_15_50_1$V4/8.506508084,Novo76_15_50_1$V4/8.506508084,Novo76_15_50_1$V4/8.506508084), add=TRUE, inches=FALSE, fg = 1:N, bg="transparent")

palette("default")

})

Bfast76_15_1_2=read.table("Bfast_76_1.50_1_2")

BWA76_15_1_2=read.table("BWA_76_1.50_1_2")

Smalt76_15_1_2=read.table("Smalt_76_1.50_1_2")

Stampy76_15_1_2=read.table("Stampy_76_1.50_1_2")

Novo76_15_1_2=read.table("Novo_76_1.50_1_2")

N <- nrow(Bfast76_15_1_2)

with(Bfast76_15_1_2, {

palette(rainbow(N, end = 0.5))

symbols(Bfast76_15_1_2$V3, Bfast76_15_1_2$V2, circles=Bfast76_15_1_2$V4/20, inches=FALSE, fg = 1:N, bg="transparent", , main="B ",font.main=2,cex.main=1,sub="1-10kb",cex.sub=1,font.sub=2,xlab="",ylab="",xlim=c(-40,110),ylim=c(-40,120))

symbols(BWA76_15_1_2$V3, BWA76_15_1_2$V2, squares=BWA76_15_1_2$V4/10, add=TRUE, inches=FALSE, fg = 1:N, bg="transparent")

symbols(Smalt76_15_1_2$V3, Smalt76_15_1_2$V2, rectangles=cbind(Smalt76_15_1_2$V4/15,Smalt76_15_1_2$V4/10), add=TRUE, inches=FALSE, fg = 1:N, bg="transparent")

symbols(Stampy76_15_1_2$V3, Stampy76_15_1_2$V2, stars=cbind(Stampy76_15_1_2$V4/5.773502692,Stampy76_15_1_2$V4/5.773502692,Stampy76_15_1_2$V4/5.773502692), add=TRUE, inches=FALSE, fg = 1:N, bg="transparent")

symbols(Novo76_15_1_2$V3, Novo76_15_1_2$V2, stars=cbind(Novo76_15_1_2$V4/8.506508084,Novo76_15_1_2$V4/8.506508084,Novo76_15_1_2$V4/8.506508084,Novo76_15_1_2$V4/8.506508084,Novo76_15_1_2$V4/8.506508084), add=TRUE, inches=FALSE, fg = 1:N, bg="transparent")
```

```
        palette("default")

    })

Bfast76_15_10_2=read.table("Bfast_76_1.50_10_2")

BWA76_15_10_2=read.table("BWA_76_1.50_10_2")

Smalt76_15_10_2=read.table("Smalt_76_1.50_10_2")

Stampy76_15_10_2=read.table("Stampy_76_1.50_10_2")

Novo76_15_10_2=read.table("Novo_76_1.50_10_2")


N <- nrow(Bfast76_15_10_2)

    with(Bfast76_15_10_2, {

            palette(rainbow(N, end = 0.5))

            symbols(Bfast76_15_10_2$V3, Bfast76_15_10_2$V2, circles=Bfast76_15_10_2$V4/20, inches=FALSE, fg = 1:N, bg="transparent", main="CNV detection tools",cex.main=1,font.main=2, sub="10-50kb",cex.sub=1,font.sub=2,xlab="",ylab="",xlim=c(-40,110),ylim=c(-40,120),yaxt="n")

            symbols(BWA76_15_10_2$V3, BWA76_15_10_2$V2, squares=BWA76_15_10_2$V4/10, add=TRUE, inches=FALSE, fg = 1:N, bg="transparent")

            symbols(Smalt76_15_10_2$V3, Smalt76_15_10_2$V2, rectangles=cbind(Smalt76_15_10_2$V4/15,Smalt76_15_10_2$V4/10), add=TRUE, inches=FALSE, fg = 1:N, bg="transparent")

            symbols(Stampy76_15_10_2$V3, Stampy76_15_10_2$V2, stars=cbind(Stampy76_15_10_2$V4/5.773502692,Stampy76_15_10_2$V4/5.773502692,Stampy76_15_10_2$V4/5.773502692), add=TRUE, inches=FALSE, fg = 1:N, bg="transparent")

            symbols(Novo76_15_10_2$V3, Novo76_15_10_2$V2, stars=cbind(Novo76_15_10_2$V4/8.506508084,Novo76_15_10_2$V4/8.506508084,Novo76_15_10_2$V4/8.506508084,Novo76_15_10_2$V4/8.506508084,Novo76_15_10_2$V4/8.506508084), add=TRUE, inches=FALSE, fg = 1:N, bg="transparent")


        palette("default")

    })

Bfast76_15_50_2=read.table("Bfast_76_1.50_50_2")

BWA76_15_50_2=read.table("BWA_76_1.50_50_2")
```

```
Smalt76_15_50_2=read.table("Smalt_76_1.50_50_2")

Stampy76_15_50_2=read.table("Stampy_76_1.50_50_2")

Novo76_15_50_2=read.table("Novo_76_1.50_50_2")

N <- nrow(Bfast76_15_50_2)

  with(Bfast76_15_50_2, {

        palette(rainbow(N, end = 0.5))

        symbols(Bfast76_15_50_2$V3, Bfast76_15_50_2$V2, circles=Bfast76_15_50_2$V4/20, 
inches=FALSE, fg = 1:N, bg="transparent", sub="50kb-
1mb",cex.sub=1,font.sub=2,xlab="",ylab="",xlim=c(-40,110),ylim=c(-40,120),yaxt="n")

        legend (80,-
25,Bfast76_15_50_2$V1,col=1:N,box.col="transparent",text.col="black",fill=1:N,cex=0.5)

        legend (55,-17,legend=leg.txt, box.col="transparent",col="black", 
pch=c(1,0,39,2,11),cex=0.6)

        symbols(BWA76_15_50_2$V3, BWA76_15_50_2$V2, squares=BWA76_15_50_2$V4/10, 
add=TRUE, inches=FALSE, fg = 1:N, bg="transparent")

        symbols(Smalt76_15_50_2$V3, Smalt76_15_50_2$V2, 
rectangles=cbind(Smalt76_15_50_2$V4/15,Smalt76_15_50_2$V4/10), add=TRUE, 
inches=FALSE, fg = 1:N, bg="transparent")

        symbols(Stampy76_15_50_2$V3, Stampy76_15_50_2$V2, 
stars=cbind(Stampy76_15_50_2$V4/5.773502692,Stampy76_15_50_2$V4/5.773502692,Stampy76
_15_50_2$V4/5.773502692), add=TRUE, inches=FALSE, fg = 1:N, bg="transparent")

        symbols(Novo76_15_50_2$V3, Novo76_15_50_2$V2, 
stars=cbind(Novo76_15_50_2$V4/8.506508084,Novo76_15_50_2$V4/8.506508084,Novo76_15_5
0_2$V4/8.506508084,Novo76_15_50_2$V4/8.506508084,Novo76_15_50_2$V4/8.506508084), 
add=TRUE, inches=FALSE, fg = 1:N, bg="transparent")



     palette("default")

  })
```

R code to generate Supporting Figure 3:

```
Bfast36_5=read.table("Bfast_36_0.05")

BWA36_5=read.table("BWA_36_0.05")

Smalt36_5=read.table("Smalt_36_0.05")
```

```
Stampy36_5=read.table("Stampy_36_0.05")

Novo36_5=read.table("Novo_36_0.05")

Bfast36_10=read.table("Bfast_36_0.10")

BWA36_10=read.table("BWA_36_0.10")

Smalt36_10=read.table("Smalt_36_0.10")

Stampy36_10=read.table("Stampy_36_0.10")

Novo36_10=read.table("Novo_36_0.10")

Bfast36_15_1=read.table("Bfast_36_1.50_1")

BWA36_15_1=read.table("BWA_36_1.50_1")

Smalt36_15_1=read.table("Smalt_36_1.50_1")

Stampy36_15_1=read.table("Stampy_36_1.50_1")

Novo36_15_1=read.table("Novo_36_1.50_1")

Bfast36_15_10=read.table("Bfast_36_1.50_10")

BWA36_15_10=read.table("BWA_36_1.50_10")

Smalt36_15_10=read.table("Smalt_36_1.50_10")

Stampy36_15_10=read.table("Stampy_36_1.50_10")

Novo36_15_10=read.table("Novo_36_1.50_10")

Bfast36_15_50=read.table("Bfast_36_1.50_50")

BWA36_15_50=read.table("BWA_36_1.50_50")

Smalt36_15_50=read.table("Smalt_36_1.50_50")

Stampy36_15_50=read.table("Stampy_36_1.50_50")

Novo36_15_50=read.table("Novo_36_1.50_50")

Bfast36_3=read.table("Bfast_36_3")

BWA36_3=read.table("BWA_36_3")

Smalt36_3=read.table("Smalt_36_3")

Stampy36_3=read.table("Stampy_36_3")
```

```
Novo36_3=read.table("Novo_36_3")

par(mar=c(5,2,1,1),mfrow=c(2,3))

leg.txt <- c("Bfast", "BWA", "Novo", "Smalt", "Stampy")

N <- nrow(Bfast36_5)

    with(Bfast36_5, {

        palette(rainbow(N+1, end = 0.8))

        symbols(Bfast36_5$V3, Bfast36_5$V2, circles=Bfast36_5$V4/20, inches=FALSE, fg = 1:N, bg="transparent", main="A              0.05% (1-10kb) ",font.main=2,cex.main=1,xlab="",ylab="",xlim=c(-15,160),ylim=c(-40,140))

        symbols(BWA36_5$V3, BWA36_5$V2, squares=BWA36_5$V4/10, add=TRUE, inches=FALSE, fg = 1:N, bg="transparent")

        symbols(Smalt36_5$V3, Smalt36_5$V2, rectangles=cbind(Smalt36_5$V4/15,Smalt36_5$V4/10), add=TRUE, inches=FALSE, fg = 1:N, bg="transparent")

        symbols(Stampy36_5$V3, Stampy36_5$V2, stars=cbind(Stampy36_5$V4/5.773502692,Stampy36_5$V4/5.773502692,Stampy36_5$V4/5.773502692), add=TRUE, inches=FALSE, fg = 1:N, bg="transparent")

        symbols(Novo36_5$V3, Novo36_5$V2, stars=cbind(Novo36_5$V4/8.506508084,Novo36_5$V4/8.506508084,Novo36_5$V4/8.506508084, Novo36_5$V4/8.506508084,Novo36_5$V4/8.506508084), add=TRUE, inches=FALSE, fg = 1:N, bg="transparent")



      palette("default")

    })

N <- nrow(Bfast36_10)

    with(Bfast36_10, {

        palette(rainbow(N+1, end = 0.8))

        symbols(Bfast36_10$V3, Bfast36_10$V2, circles=Bfast36_10$V4/20, inches=FALSE, fg = 1:N, bg="transparent", main="0.10% (10-50kb)",font.main=2,cex.main=1,xlab="",ylab="",xlim=c(-15,160),ylim=c(-40,140))

        symbols(BWA36_10$V3, BWA36_10$V2, squares=BWA36_10$V4/10, add=TRUE, inches=FALSE, fg = 1:N, bg="transparent")
```

```
        symbols(Smalt36_10$V3, Smalt36_10$V2,
rectangles=cbind(Smalt36_10$V4/15,Smalt36_10$V4/10), add=TRUE, inches=FALSE, fg = 1:N,
bg="transparent")

        symbols(Stampy36_10$V3, Stampy36_10$V2,
stars=cbind(Stampy36_10$V4/5.773502692,Stampy36_10$V4/5.773502692,Stampy36_10$V4/5.7
73502692), add=TRUE, inches=FALSE, fg = 1:N, bg="transparent")

        symbols(Novo36_10$V3, Novo36_10$V2,
stars=cbind(Novo36_10$V4/8.506508084,Novo36_10$V4/8.506508084,Novo36_10$V4/8.506508
084,Novo36_10$V4/8.506508084,Novo36_10$V4/8.506508084), add=TRUE, inches=FALSE, fg
= 1:N, bg="transparent")



      palette("default")

   })

N <- nrow(Bfast36_15_1)

   with(Bfast36_15_1, {

        palette(rainbow(N+1, end = 0.8))

        symbols(Bfast36_15_1$V3, Bfast36_15_1$V2, circles=Bfast36_15_1$V4/20,
inches=FALSE, fg = 1:N, bg="transparent", main="1.50% (1-
10kb)",font.main=2,cex.main=1,xlab="",ylab="",xlim=c(-15,160),ylim=c(-40,140))

        symbols(BWA36_15_1$V3, BWA36_15_1$V2, squares=BWA36_15_1$V4/10,
add=TRUE, inches=FALSE, fg = 1:N, bg="transparent")

        symbols(Smalt36_15_1$V3, Smalt36_15_1$V2,
rectangles=cbind(Smalt36_15_1$V4/15,Smalt36_15_1$V4/10), add=TRUE, inches=FALSE, fg =
1:N, bg="transparent")

        symbols(Stampy36_15_1$V3, Stampy36_15_1$V2,
stars=cbind(Stampy36_15_1$V4/5.773502692,Stampy36_15_1$V4/5.773502692,Stampy36_15_1$
V4/5.773502692), add=TRUE, inches=FALSE, fg = 1:N, bg="transparent")

        symbols(Novo36_15_1$V3, Novo36_15_1$V2,
stars=cbind(Novo36_15_1$V4/8.506508084,Novo36_15_1$V4/8.506508084,Novo36_15_1$V4/8.
506508084,Novo36_15_1$V4/8.506508084,Novo36_10$V4/8.506508084), add=TRUE,
inches=FALSE, fg = 1:N, bg="transparent")



      palette("default")

   })

N <- nrow(Bfast36_15_10)
```

```
    with(Bfast36_15_10, {

          palette(rainbow(N+1, end = 0.8))

          symbols(Bfast36_15_10$V3, Bfast36_15_10$V2, circles=Bfast36_15_10$V4/20,
inches=FALSE, fg = 1:N, bg="transparent", main="1.50% (10-
50kb)",font.main=2,cex.main=1,sub="Read length (36 nts)
",font.sub=2,cex.sub=1,xlab="",ylab="",xlim=c(-15,160),ylim=c(-40,140))

          symbols(BWA36_15_10$V3, BWA36_15_10$V2, squares=BWA36_15_10$V4/10,
add=TRUE, inches=FALSE, fg = 1:N, bg="transparent")

          symbols(Smalt36_15_10$V3, Smalt36_15_10$V2,
rectangles=cbind(Smalt36_15_10$V4/15,Smalt36_15_10$V4/10), add=TRUE, inches=FALSE, fg
= 1:N, bg="transparent")

          symbols(Stampy36_15_10$V3, Stampy36_15_10$V2,
stars=cbind(Stampy36_15_10$V4/5.773502692,Stampy36_15_10$V4/5.773502692,Stampy36_15_
10$V4/5.773502692), add=TRUE, inches=FALSE, fg = 1:N, bg="transparent")

          symbols(Novo36_15_10$V3, Novo36_15_10$V2,
stars=cbind(Novo36_15_10$V4/8.506508084,Novo36_15_10$V4/8.506508084,Novo36_15_10$V
4/8.506508084,Novo36_15_10$V4/8.506508084,Novo36_10$V4/8.506508084), add=TRUE,
inches=FALSE, fg = 1:N, bg="transparent")



       palette("default")

    })

N <- nrow(Bfast36_15_50)

    with(Bfast36_15_50, {

          palette(rainbow(N+1, end = 0.8))

          symbols(Bfast36_15_50$V3, Bfast36_15_50$V2, circles=Bfast36_15_50$V4/20,
inches=FALSE, fg = 1:N, bg="transparent", main="1.50% (50kb-
1mb)",font.main=2,cex.main=1,xlab="",ylab="",xlim=c(-15,160),ylim=c(-40,140))

          symbols(BWA36_15_50$V3, BWA36_15_50$V2, squares=BWA36_15_50$V4/10,
add=TRUE, inches=FALSE, fg = 1:N, bg="transparent")

          symbols(Smalt36_15_50$V3, Smalt36_15_50$V2,
rectangles=cbind(Smalt36_15_50$V4/15,Smalt36_15_50$V4/10), add=TRUE, inches=FALSE, fg
= 1:N, bg="transparent")

          symbols(Stampy36_15_50$V3, Stampy36_15_50$V2,
stars=cbind(Stampy36_15_50$V4/5.773502692,Stampy36_15_50$V4/5.773502692,Stampy36_15_
50$V4/5.773502692), add=TRUE, inches=FALSE, fg = 1:N, bg="transparent")
```

```
symbols(Novo36_15_50$V3, Novo36_15_50$V2, stars=cbind(Novo36_15_50$V4/8.506508084,Novo36_15_50$V4/8.506508084,Novo36_15_50$V4/8.506508084,Novo36_15_50$V4/8.506508084,Novo36_10$V4/8.506508084), add=TRUE, inches=FALSE, fg = 1:N, bg="transparent")

palette("default")

})

N <- nrow(Bfast36_3)

with(Bfast36_3, {

palette(rainbow(N+1, end = 0.8))

symbols(Bfast36_3$V3, Bfast36_3$V2, circles=Bfast36_3$V4/20, inches=FALSE, fg = 1:N, bg="transparent", main="3% (1-5mb)",font.main=2,cex.main=1,xlab="",ylab="",xlim=c(-15,160),ylim=c(-40,140))

legend (110,-5,Bfast36_3$V1,col=1:N,box.col="transparent",text.col="black",fill=1:N,cex=0.5)

legend (80,-4,legend=leg.txt, box.col="transparent",col="black", pch=c(1,0,39,2,11),cex=0.6)

symbols(BWA36_3$V3, BWA36_3$V2, squares=BWA36_3$V4/10, add=TRUE, inches=FALSE, fg = 1:N, bg="transparent")

symbols(Smalt36_3$V3, Smalt36_3$V2, rectangles=cbind(Smalt36_3$V4/15,Smalt36_3$V4/10), add=TRUE, inches=FALSE, fg = 1:N, bg="transparent")

symbols(Stampy36_3$V3, Stampy36_3$V2, stars=cbind(Stampy36_3$V4/5.773502692,Stampy36_3$V4/5.773502692,Stampy36_3$V4/5.773502692), add=TRUE, inches=FALSE, fg = 1:N, bg="transparent")

symbols(Novo36_3$V3, Novo36_3$V2, stars=cbind(Novo36_3$V4/8.506508084,Novo36_3$V4/8.506508084,Novo36_3$V4/8.506508084,Novo36_3$V4/8.506508084,Novo36_10$V4/8.506508084), add=TRUE, inches=FALSE, fg = 1:N, bg="transparent")

palette("default")

})

Bfast50_5=read.table("Bfast_50_0.05")
```

```
BWA50_5=read.table("BWA_50_0.05")

Smalt50_5=read.table("Smalt_50_0.05")

Stampy50_5=read.table("Stampy_50_0.05")

Novo50_5=read.table("Novo_50_0.05")

Bfast50_10=read.table("Bfast_50_0.10")

BWA50_10=read.table("BWA_50_0.10")

Smalt50_10=read.table("Smalt_50_0.10")

Stampy50_10=read.table("Stampy_50_0.10")

Novo50_10=read.table("Novo_50_0.10")

Bfast50_15_1=read.table("Bfast_50_1.50_1")

BWA50_15_1=read.table("BWA_50_1.50_1")

Smalt50_15_1=read.table("Smalt_50_1.50_1")

Stampy50_15_1=read.table("Stampy_50_1.50_1")

Novo50_15_1=read.table("Novo_50_1.50_1")

Bfast50_15_10=read.table("Bfast_50_1.50_10")

BWA50_15_10=read.table("BWA_50_1.50_10")

Smalt50_15_10=read.table("Smalt_50_1.50_10")

Stampy50_15_10=read.table("Stampy_50_1.50_10")

Novo50_15_10=read.table("Novo_50_1.50_10")

Bfast50_15_50=read.table("Bfast_50_1.50_50")

BWA50_15_50=read.table("BWA_50_1.50_50")

Smalt50_15_50=read.table("Smalt_50_1.50_50")

Stampy50_15_50=read.table("Stampy_50_1.50_50")

Novo50_15_50=read.table("Novo_50_1.50_50")

Bfast50_3=read.table("Bfast_50_3")

BWA50_3=read.table("BWA_50_3")
```

```
Smalt50_3=read.table("Smalt_50_3")

Stampy50_3=read.table("Stampy_50_3")

Novo50_3=read.table("Novo_50_3")

par(mar=c(5,2,1,1),mfrow=c(2,3))

N <- nrow(Bfast50_5)

    with(Bfast50_5, {

        palette(rainbow(N+1, end = 0.8))

        symbols(Bfast50_5$V3, Bfast50_5$V2, circles=Bfast50_5$V4/20, inches=FALSE, fg = 1:N, bg="transparent", main="B              0.05% (1-10kb)
",font.main=2,cex.main=1,xlab="",ylab="",xlim=c(-30,170),ylim=c(-50,150))

        symbols(BWA50_5$V3, BWA50_5$V2, squares=BWA50_5$V4/10, add=TRUE, inches=FALSE, fg = 1:N, bg="transparent")

        symbols(Smalt50_5$V3, Smalt50_5$V2, rectangles=cbind(Smalt50_5$V4/15,Smalt50_5$V4/10), add=TRUE, inches=FALSE, fg = 1:N, bg="transparent")

        symbols(Stampy50_5$V3, Stampy50_5$V2, stars=cbind(Stampy50_5$V4/5.773502692,Stampy50_5$V4/5.773502692,Stampy50_5$V4/5.773502692), add=TRUE, inches=FALSE, fg = 1:N, bg="transparent")

        symbols(Novo50_5$V3, Novo50_5$V2, stars=cbind(Novo50_5$V4/8.506508084,Novo50_5$V4/8.506508084,Novo50_5$V4/8.506508084, Novo50_5$V4/8.506508084,Novo50_5$V4/8.506508084), add=TRUE, inches=FALSE, fg = 1:N, bg="transparent")



      palette("default")

    })

N <- nrow(Bfast50_10)

    with(Bfast50_10, {

        palette(rainbow(N+1, end = 0.8))

        symbols(Bfast50_10$V3, Bfast50_10$V2, circles=Bfast50_10$V4/20, inches=FALSE, fg = 1:N, bg="transparent", main="0.10% (10-50kb)",font.main=2,cex.main=1,xlab="",ylab="",xlim=c(-30,170),ylim=c(-50,150))

        symbols(BWA50_10$V3, BWA50_10$V2, squares=BWA50_10$V4/10, add=TRUE, inches=FALSE, fg = 1:N, bg="transparent")
```

```
        symbols(Smalt50_10$V3, Smalt50_10$V2,
rectangles=cbind(Smalt50_10$V4/15,Smalt50_10$V4/10), add=TRUE, inches=FALSE, fg = 1:N,
bg="transparent")

        symbols(Stampy50_10$V3, Stampy50_10$V2,
stars=cbind(Stampy50_10$V4/5.773502692,Stampy50_10$V4/5.773502692,Stampy50_10$V4/5.7
73502692), add=TRUE, inches=FALSE, fg = 1:N, bg="transparent")

        symbols(Novo50_10$V3, Novo50_10$V2,
stars=cbind(Novo50_10$V4/8.506508084,Novo50_10$V4/8.506508084,Novo50_10$V4/8.506508
084,Novo50_10$V4/8.506508084,Novo50_10$V4/8.506508084), add=TRUE, inches=FALSE, fg
= 1:N, bg="transparent")



      palette("default")

    })

N <- nrow(Bfast50_15_1)

    with(Bfast50_15_1, {

        palette(rainbow(N+1, end = 0.8))

        symbols(Bfast50_15_1$V3, Bfast50_15_1$V2, circles=Bfast50_15_1$V4/20,
inches=FALSE, fg = 1:N, bg="transparent", main="1.50% (1-
10kb)",font.main=2,cex.main=1,xlab="",ylab="",xlim=c(-30,170),ylim=c(-50,150))

        symbols(BWA50_15_1$V3, BWA50_15_1$V2, squares=BWA50_15_1$V4/10,
add=TRUE, inches=FALSE, fg = 1:N, bg="transparent")

        symbols(Smalt50_15_1$V3, Smalt50_15_1$V2,
rectangles=cbind(Smalt50_15_1$V4/15,Smalt50_15_1$V4/10), add=TRUE, inches=FALSE, fg =
1:N, bg="transparent")

        symbols(Stampy50_15_1$V3, Stampy50_15_1$V2,
stars=cbind(Stampy50_15_1$V4/5.773502692,Stampy50_15_1$V4/5.773502692,Stampy50_15_1$
V4/5.773502692), add=TRUE, inches=FALSE, fg = 1:N, bg="transparent")

        symbols(Novo50_15_1$V3, Novo50_15_1$V2,
stars=cbind(Novo50_15_1$V4/8.506508084,Novo50_15_1$V4/8.506508084,Novo50_15_1$V4/8.
506508084,Novo50_15_1$V4/8.506508084,Novo50_10$V4/8.506508084), add=TRUE,
inches=FALSE, fg = 1:N, bg="transparent")



      palette("default")

    })
```

```
N <- nrow(Bfast50_15_10)

    with(Bfast50_15_10, {

          palette(rainbow(N+1, end = 0.8))

          symbols(Bfast50_15_10$V3, Bfast50_15_10$V2, circles=Bfast50_15_10$V4/20,
inches=FALSE, fg = 1:N, bg="transparent", main="1.50% (10-
50kb)",font.main=2,cex.main=1,sub="Read length (50 nts)
",font.sub=2,cex.sub=1,xlab="",ylab="",xlim=c(-30,170),ylim=c(-50,150))

          symbols(BWA50_15_10$V3, BWA50_15_10$V2, squares=BWA50_15_10$V4/10,
add=TRUE, inches=FALSE, fg = 1:N, bg="transparent")

          symbols(Smalt50_15_10$V3, Smalt50_15_10$V2,
rectangles=cbind(Smalt50_15_10$V4/15,Smalt50_15_10$V4/10), add=TRUE, inches=FALSE, fg
= 1:N, bg="transparent")

          symbols(Stampy50_15_10$V3, Stampy50_15_10$V2,
stars=cbind(Stampy50_15_10$V4/5.773502692,Stampy50_15_10$V4/5.773502692,Stampy50_15_
10$V4/5.773502692), add=TRUE, inches=FALSE, fg = 1:N, bg="transparent")

          symbols(Novo50_15_10$V3, Novo50_15_10$V2,
stars=cbind(Novo50_15_10$V4/8.506508084,Novo50_15_10$V4/8.506508084,Novo50_15_10$V
4/8.506508084,Novo50_15_10$V4/8.506508084,Novo50_10$V4/8.506508084), add=TRUE,
inches=FALSE, fg = 1:N, bg="transparent")



       palette("default")

    })

N <- nrow(Bfast50_15_50)

    with(Bfast50_15_50, {

          palette(rainbow(N+1, end = 0.8))

          symbols(Bfast50_15_50$V3, Bfast50_15_50$V2, circles=Bfast50_15_50$V4/20,
inches=FALSE, fg = 1:N, bg="transparent", main="1.50% (50kb-
1mb)",font.main=2,cex.main=1,xlab="",ylab="",xlim=c(-30,170),ylim=c(-50,150))

          symbols(BWA50_15_50$V3, BWA50_15_50$V2, squares=BWA50_15_50$V4/10,
add=TRUE, inches=FALSE, fg = 1:N, bg="transparent")

          symbols(Smalt50_15_50$V3, Smalt50_15_50$V2,
rectangles=cbind(Smalt50_15_50$V4/15,Smalt50_15_50$V4/10), add=TRUE, inches=FALSE, fg
= 1:N, bg="transparent")

          symbols(Stampy50_15_50$V3, Stampy50_15_50$V2,
stars=cbind(Stampy50_15_50$V4/5.773502692,Stampy50_15_50$V4/5.773502692,Stampy50_15_
```

```
50$V4/5.773502692), add=TRUE, inches=FALSE, fg = 1:N, bg="transparent")

        symbols(Novo50_15_50$V3, Novo50_15_50$V2,
stars=cbind(Novo50_15_50$V4/8.506508084,Novo50_15_50$V4/8.506508084,Novo50_15_50$V
4/8.506508084,Novo50_15_50$V4/8.506508084,Novo50_10$V4/8.506508084), add=TRUE,
inches=FALSE, fg = 1:N, bg="transparent")



      palette("default")

    })

N <- nrow(Bfast50_3)

    with(Bfast50_3, {

        palette(rainbow(N+1, end = 0.8))

        symbols(Bfast50_3$V3, Bfast50_3$V2, circles=Bfast50_3$V4/20, inches=FALSE, fg =
1:N, bg="transparent", main="3% (1-5mb)",font.main=2,cex.main=1,xlab="",ylab="",xlim=c(-
30,170),ylim=c(-50,150))

        legend (110,-
5,Bfast50_3$V1,col=1:N,box.col="transparent",text.col="black",fill=1:N,cex=0.5)

        legend (80,-4,legend=leg.txt, box.col="transparent",col="black",
pch=c(1,0,39,2,11),cex=0.6)

        symbols(BWA50_3$V3, BWA50_3$V2, squares=BWA50_3$V4/10, add=TRUE,
inches=FALSE, fg = 1:N, bg="transparent")

        symbols(Smalt50_3$V3, Smalt50_3$V2,
rectangles=cbind(Smalt50_3$V4/15,Smalt50_3$V4/10), add=TRUE, inches=FALSE, fg = 1:N,
bg="transparent")

        symbols(Stampy50_3$V3, Stampy50_3$V2,
stars=cbind(Stampy50_3$V4/5.773502692,Stampy50_3$V4/5.773502692,Stampy50_3$V4/5.7735
02692), add=TRUE, inches=FALSE, fg = 1:N, bg="transparent")

        symbols(Novo50_3$V3, Novo50_3$V2,
stars=cbind(Novo50_3$V4/8.506508084,Novo50_3$V4/8.506508084,Novo50_3$V4/8.506508084,
Novo50_3$V4/8.506508084,Novo50_10$V4/8.506508084), add=TRUE, inches=FALSE, fg = 1:N,
bg="transparent")



      palette("default")

    })

Bfast76_5=read.table("Bfast_76_0.05")
```

```
BWA76_5=read.table("BWA_76_0.05")
Smalt76_5=read.table("Smalt_76_0.05")
Stampy76_5=read.table("Stampy_76_0.05")
Novo76_5=read.table("Novo_76_0.05")
Bfast76_10=read.table("Bfast_76_0.10")
BWA76_10=read.table("BWA_76_0.10")
Smalt76_10=read.table("Smalt_76_0.10")
Stampy76_10=read.table("Stampy_76_0.10")
Novo76_10=read.table("Novo_76_0.10")
Bfast76_15_1=read.table("Bfast_76_1.50_1")
BWA76_15_1=read.table("BWA_76_1.50_1")
Smalt76_15_1=read.table("Smalt_76_1.50_1")
Stampy76_15_1=read.table("Stampy_76_1.50_1")
Novo76_15_1=read.table("Novo_76_1.50_1")
Bfast76_15_10=read.table("Bfast_76_1.50_10")
BWA76_15_10=read.table("BWA_76_1.50_10")
Smalt76_15_10=read.table("Smalt_76_1.50_10")
Stampy76_15_10=read.table("Stampy_76_1.50_10")
Novo76_15_10=read.table("Novo_76_1.50_10")
Bfast76_15_50=read.table("Bfast_76_1.50_50")
BWA76_15_50=read.table("BWA_76_1.50_50")
Smalt76_15_50=read.table("Smalt_76_1.50_50")
Stampy76_15_50=read.table("Stampy_76_1.50_50")
Novo76_15_50=read.table("Novo_76_1.50_50")
Bfast76_3=read.table("Bfast_76_3")
BWA76_3=read.table("BWA_76_3")
```

```
Smalt76_3=read.table("Smalt_76_3")

Stampy76_3=read.table("Stampy_76_3")

Novo76_3=read.table("Novo_76_3")

par(mar=c(5,2,1,1),mfrow=c(2,3))

N <- nrow(Bfast76_5)

    with(Bfast76_5, {

          palette(rainbow(N, end = 0.8))

          symbols(Bfast76_5$V3, Bfast76_5$V2, circles=Bfast76_5$V4/20, inches=FALSE, fg = 1:N, bg="transparent", main="C              0.05% (1-10kb)
",font.main=2,cex.main=1,xlab="",ylab="",xlim=c(-50,170),ylim=c(-50,140))

          symbols(BWA76_5$V3, BWA76_5$V2, squares=BWA76_5$V4/10, add=TRUE, inches=FALSE, fg = 1:N, bg="transparent")

          symbols(Smalt76_5$V3, Smalt76_5$V2, rectangles=cbind(Smalt76_5$V4/15,Smalt76_5$V4/10), add=TRUE, inches=FALSE, fg = 1:N, bg="transparent")

          symbols(Stampy76_5$V3, Stampy76_5$V2, stars=cbind(Stampy76_5$V4/5.773502692,Stampy76_5$V4/5.773502692,Stampy76_5$V4/5.773502692), add=TRUE, inches=FALSE, fg = 1:N, bg="transparent")

          symbols(Novo76_5$V3, Novo76_5$V2, stars=cbind(Novo76_5$V4/8.506508084,Novo76_5$V4/8.506508084,Novo76_5$V4/8.506508084, Novo76_5$V4/8.506508084,Novo76_5$V4/8.506508084), add=TRUE, inches=FALSE, fg = 1:N, bg="transparent")



       palette("default")

    })

N <- nrow(Bfast76_10)

    with(Bfast76_10, {

          palette(rainbow(N, end = 0.8))

          symbols(Bfast76_10$V3, Bfast76_10$V2, circles=Bfast76_10$V4/20, inches=FALSE, fg = 1:N, bg="transparent", main="0.10% (10-50kb)",font.main=2,cex.main=1,xlab="",ylab="",xlim=c(-50,170),ylim=c(-50,140))

          symbols(BWA76_10$V3, BWA76_10$V2, squares=BWA76_10$V4/10, add=TRUE,
```

```
inches=FALSE, fg = 1:N, bg="transparent")

        symbols(Smalt76_10$V3, Smalt76_10$V2,
rectangles=cbind(Smalt76_10$V4/15,Smalt76_10$V4/10), add=TRUE, inches=FALSE, fg = 1:N,
bg="transparent")

        symbols(Stampy76_10$V3, Stampy76_10$V2,
stars=cbind(Stampy76_10$V4/5.773502692,Stampy76_10$V4/5.773502692,Stampy76_10$V4/5.7
73502692), add=TRUE, inches=FALSE, fg = 1:N, bg="transparent")

        symbols(Novo76_10$V3, Novo76_10$V2,
stars=cbind(Novo76_10$V4/8.506508084,Novo76_10$V4/8.506508084,Novo76_10$V4/8.506508
084,Novo76_10$V4/8.506508084,Novo76_10$V4/8.506508084), add=TRUE, inches=FALSE, fg
= 1:N, bg="transparent")



      palette("default")

    })

N <- nrow(Bfast76_15_1)

    with(Bfast76_15_1, {

        palette(rainbow(N, end = 0.8))

        symbols(Bfast76_15_1$V3, Bfast76_15_1$V2, circles=Bfast76_15_1$V4/20,
inches=FALSE, fg = 1:N, bg="transparent", main="1.50% (1-
10kb)",font.main=2,cex.main=1,xlab="",ylab="",xlim=c(-50,170),ylim=c(-50,140))

        symbols(BWA76_15_1$V3, BWA76_15_1$V2, squares=BWA76_15_1$V4/10,
add=TRUE, inches=FALSE, fg = 1:N, bg="transparent")

        symbols(Smalt76_15_1$V3, Smalt76_15_1$V2,
rectangles=cbind(Smalt76_15_1$V4/15,Smalt76_15_1$V4/10), add=TRUE, inches=FALSE, fg =
1:N, bg="transparent")

        symbols(Stampy76_15_1$V3, Stampy76_15_1$V2,
stars=cbind(Stampy76_15_1$V4/5.773502692,Stampy76_15_1$V4/5.773502692,Stampy76_15_1$
V4/5.773502692), add=TRUE, inches=FALSE, fg = 1:N, bg="transparent")

        symbols(Novo76_15_1$V3, Novo76_15_1$V2,
stars=cbind(Novo76_15_1$V4/8.506508084,Novo76_15_1$V4/8.506508084,Novo76_15_1$V4/8.
506508084,Novo76_15_1$V4/8.506508084,Novo76_10$V4/8.506508084), add=TRUE,
inches=FALSE, fg = 1:N, bg="transparent")



      palette("default")

    })
```

```
N <- nrow(Bfast76_15_10)

    with(Bfast76_15_10, {

        palette(rainbow(N, end = 0.8))

        symbols(Bfast76_15_10$V3, Bfast76_15_10$V2, circles=Bfast76_15_10$V4/20,
inches=FALSE, fg = 1:N, bg="transparent", main="1.50% (10-
50kb)",font.main=2,cex.main=1,xlab="",ylab="",sub="Read length (76 nts)
",font.sub=2,cex.sub=1,xlim=c(-50,170),ylim=c(-50,140))

        symbols(BWA76_15_10$V3, BWA76_15_10$V2, squares=BWA76_15_10$V4/10,
add=TRUE, inches=FALSE, fg = 1:N, bg="transparent")

        symbols(Smalt76_15_10$V3, Smalt76_15_10$V2,
rectangles=cbind(Smalt76_15_10$V4/15,Smalt76_15_10$V4/10), add=TRUE, inches=FALSE, fg
= 1:N, bg="transparent")

        symbols(Stampy76_15_10$V3, Stampy76_15_10$V2,
stars=cbind(Stampy76_15_10$V4/5.773502692,Stampy76_15_10$V4/5.773502692,Stampy76_15_
10$V4/5.773502692), add=TRUE, inches=FALSE, fg = 1:N, bg="transparent")

        symbols(Novo76_15_10$V3, Novo76_15_10$V2,
stars=cbind(Novo76_15_10$V4/8.506508084,Novo76_15_10$V4/8.506508084,Novo76_15_10$V
4/8.506508084,Novo76_15_10$V4/8.506508084,Novo76_10$V4/8.506508084), add=TRUE,
inches=FALSE, fg = 1:N, bg="transparent")



      palette("default")

    })

N <- nrow(Bfast76_15_50)

    with(Bfast76_15_50, {

        palette(rainbow(N, end = 0.8))

        symbols(Bfast76_15_50$V3, Bfast76_15_50$V2, circles=Bfast76_15_50$V4/20,
inches=FALSE, fg = 1:N, bg="transparent", main="1.50% (50kb-
1mb)",font.main=2,cex.main=1,xlab="",ylab="",xlim=c(-50,170),ylim=c(-50,140))

        symbols(BWA76_15_50$V3, BWA76_15_50$V2, squares=BWA76_15_50$V4/10,
add=TRUE, inches=FALSE, fg = 1:N, bg="transparent")

        symbols(Smalt76_15_50$V3, Smalt76_15_50$V2,
rectangles=cbind(Smalt76_15_50$V4/15,Smalt76_15_50$V4/10), add=TRUE, inches=FALSE, fg
= 1:N, bg="transparent")

        symbols(Stampy76_15_50$V3, Stampy76_15_50$V2,
```

```
stars=cbind(Stampy76_15_50$V4/5.773502692,Stampy76_15_50$V4/5.773502692,Stampy76_15_50$V4/5.773502692), add=TRUE, inches=FALSE, fg = 1:N, bg="transparent")

        symbols(Novo76_15_50$V3, Novo76_15_50$V2, stars=cbind(Novo76_15_50$V4/8.506508084,Novo76_15_50$V4/8.506508084,Novo76_15_50$V4/8.506508084,Novo76_15_50$V4/8.506508084,Novo76_10$V4/8.506508084), add=TRUE, inches=FALSE, fg = 1:N, bg="transparent")



      palette("default")

    })

N <- nrow(Bfast76_3)

    with(Bfast76_3, {

        palette(rainbow(N, end = 0.8))

        symbols(Bfast76_3$V3, Bfast76_3$V2, circles=Bfast76_3$V4/20, inches=FALSE, fg = 1:N, bg="transparent", main="3% (1-5mb)",font.main=2,cex.main=1,xlab="",ylab="",xlim=c(-50,170),ylim=c(-50,140))

        legend (110,-15,Bfast76_3$V1,col=1:N,box.col="transparent",text.col="black",fill=1:N,cex=0.5)

        legend (70,-14,legend=leg.txt, box.col="transparent",col="black", pch=c(1,0,39,2,11),cex=0.6)

        symbols(BWA76_3$V3, BWA76_3$V2, squares=BWA76_3$V4/10, add=TRUE, inches=FALSE, fg = 1:N, bg="transparent")

        symbols(Smalt76_3$V3, Smalt76_3$V2, rectangles=cbind(Smalt76_3$V4/15,Smalt76_3$V4/10), add=TRUE, inches=FALSE, fg = 1:N, bg="transparent")

        symbols(Stampy76_3$V3, Stampy76_3$V2, stars=cbind(Stampy76_3$V4/5.773502692,Stampy76_3$V4/5.773502692,Stampy76_3$V4/5.773502692), add=TRUE, inches=FALSE, fg = 1:N, bg="transparent")

        symbols(Novo76_3$V3, Novo76_3$V2, stars=cbind(Novo76_3$V4/8.506508084,Novo76_3$V4/8.506508084,Novo76_3$V4/8.506508084,Novo76_3$V4/8.506508084,Novo76_10$V4/8.506508084), add=TRUE, inches=FALSE, fg = 1:N, bg="transparent")



      palette("default")

    })
```

```
Bfast100_5=read.table("Bfast_100_0.05")

BWA100_5=read.table("BWA_100_0.05")

Smalt100_5=read.table("Smalt_100_0.05")

Stampy100_5=read.table("Stampy_100_0.05")

Novo100_5=read.table("Novo_100_0.05")

Bfast100_10=read.table("Bfast_100_0.10")

BWA100_10=read.table("BWA_100_0.10")

Smalt100_10=read.table("Smalt_100_0.10")

Stampy100_10=read.table("Stampy_100_0.10")

Novo100_10=read.table("Novo_100_0.10")

Bfast100_15_1=read.table("Bfast_100_1.50_1")

BWA100_15_1=read.table("BWA_100_1.50_1")

Smalt100_15_1=read.table("Smalt_100_1.50_1")

Stampy100_15_1=read.table("Stampy_100_1.50_1")

Novo100_15_1=read.table("Novo_100_1.50_1")

Bfast100_15_10=read.table("Bfast_100_1.50_10")

BWA100_15_10=read.table("BWA_100_1.50_10")

Smalt100_15_10=read.table("Smalt_100_1.50_10")

Stampy100_15_10=read.table("Stampy_100_1.50_10")

Novo100_15_10=read.table("Novo_100_1.50_10")

Bfast100_15_50=read.table("Bfast_100_1.50_50")

BWA100_15_50=read.table("BWA_100_1.50_50")

Smalt100_15_50=read.table("Smalt_100_1.50_50")

Stampy100_15_50=read.table("Stampy_100_1.50_50")

Novo100_15_50=read.table("Novo_100_1.50_50")

Bfast100_3=read.table("Bfast_100_3")
```

```
BWA100_3=read.table("BWA_100_3")

Smalt100_3=read.table("Smalt_100_3")

Stampy100_3=read.table("Stampy_100_3")

Novo100_3=read.table("Novo_100_3")

par(mar=c(5,2,1,1),mfrow=c(2,3))

N <- nrow(Bfast100_5)

    with(Bfast100_5, {

          palette(rainbow(N, end = 0.8))

          symbols(Bfast100_5$V3, Bfast100_5$V2, circles=Bfast100_5$V4/20, inches=FALSE, fg = 1:N, bg="transparent", main="D              0.05% (1-10kb)
",font.main=2,cex.main=1,xlab="",ylab="",xlim=c(-10,160),ylim=c(-10,130))

          symbols(BWA100_5$V3, BWA100_5$V2, squares=BWA100_5$V4/10, add=TRUE, inches=FALSE, fg = 1:N, bg="transparent")

          symbols(Smalt100_5$V3, Smalt100_5$V2, rectangles=cbind(Smalt100_5$V4/15,Smalt100_5$V4/10), add=TRUE, inches=FALSE, fg = 1:N, bg="transparent")

          symbols(Stampy100_5$V3, Stampy100_5$V2, stars=cbind(Stampy100_5$V4/5.773502692,Stampy100_5$V4/5.773502692,Stampy100_5$V4/5.773502692), add=TRUE, inches=FALSE, fg = 1:N, bg="transparent")

          symbols(Novo100_5$V3, Novo100_5$V2, stars=cbind(Novo100_5$V4/8.506508084,Novo100_5$V4/8.506508084,Novo100_5$V4/8.506508084,Novo100_5$V4/8.506508084,Novo100_5$V4/8.506508084), add=TRUE, inches=FALSE, fg = 1:N, bg="transparent")



       palette("default")

    })

N <- nrow(Bfast100_10)

    with(Bfast100_10, {

          palette(rainbow(N, end = 0.8))

          symbols(Bfast100_10$V3, Bfast100_10$V2, circles=Bfast100_10$V4/20, inches=FALSE, fg = 1:N, bg="transparent", main="0.10% (10-
50kb)",font.main=2,cex.main=1,xlab="",ylab="",xlim=c(-10,160),ylim=c(-10,130))
```

```
        symbols(BWA100_10$V3, BWA100_10$V2, squares=BWA100_10$V4/10, add=TRUE, inches=FALSE, fg = 1:N, bg="transparent")

        symbols(Smalt100_10$V3, Smalt100_10$V2, rectangles=cbind(Smalt100_10$V4/15,Smalt100_10$V4/10), add=TRUE, inches=FALSE, fg = 1:N, bg="transparent")

        symbols(Stampy100_10$V3, Stampy100_10$V2, stars=cbind(Stampy100_10$V4/5.773502692,Stampy100_10$V4/5.773502692,Stampy100_10$V4/5.773502692), add=TRUE, inches=FALSE, fg = 1:N, bg="transparent")

        symbols(Novo100_10$V3, Novo100_10$V2, stars=cbind(Novo100_10$V4/8.506508084,Novo100_10$V4/8.506508084,Novo100_10$V4/8.506508084,Novo100_10$V4/8.506508084,Novo100_10$V4/8.506508084), add=TRUE, inches=FALSE, fg = 1:N, bg="transparent")



      palette("default")

    })

N <- nrow(Bfast100_15_1)

    with(Bfast100_15_1, {

        palette(rainbow(N, end = 0.8))

        symbols(Bfast100_15_1$V3, Bfast100_15_1$V2, circles=Bfast100_15_1$V4/20, inches=FALSE, fg = 1:N, bg="transparent", main="1.50% (1-10kb)",font.main=2,cex.main=1,xlab="",ylab="",xlim=c(-10,160),ylim=c(-10,130))

        symbols(BWA100_15_1$V3, BWA100_15_1$V2, squares=BWA100_15_1$V4/10, add=TRUE, inches=FALSE, fg = 1:N, bg="transparent")

        symbols(Smalt100_15_1$V3, Smalt100_15_1$V2, rectangles=cbind(Smalt100_15_1$V4/15,Smalt100_15_1$V4/10), add=TRUE, inches=FALSE, fg = 1:N, bg="transparent")

        symbols(Stampy100_15_1$V3, Stampy100_15_1$V2, stars=cbind(Stampy100_15_1$V4/5.773502692,Stampy100_15_1$V4/5.773502692,Stampy100_15_1$V4/5.773502692), add=TRUE, inches=FALSE, fg = 1:N, bg="transparent")

        symbols(Novo100_15_1$V3, Novo100_15_1$V2, stars=cbind(Novo100_15_1$V4/8.506508084,Novo100_15_1$V4/8.506508084,Novo100_15_1$V4/8.506508084,Novo100_15_1$V4/8.506508084,Novo100_10$V4/8.506508084), add=TRUE, inches=FALSE, fg = 1:N, bg="transparent")



      palette("default")
```

```
    })

N <- nrow(Bfast100_15_10)

    with(Bfast100_15_10, {

        palette(rainbow(N, end = 0.8))

        symbols(Bfast100_15_10$V3, Bfast100_15_10$V2, circles=Bfast100_15_10$V4/20,
inches=FALSE, fg = 1:N, bg="transparent", main="1.50% (10-
50kb)",font.main=2,cex.main=1,xlab="",ylab="",sub="Read length (100 nts)
",font.sub=2,cex.sub=1,xlim=c(-10,160),ylim=c(-10,130))

        symbols(BWA100_15_10$V3, BWA100_15_10$V2, squares=BWA100_15_10$V4/10,
add=TRUE, inches=FALSE, fg = 1:N, bg="transparent")

        symbols(Smalt100_15_10$V3, Smalt100_15_10$V2,
rectangles=cbind(Smalt100_15_10$V4/15,Smalt100_15_10$V4/10), add=TRUE, inches=FALSE,
fg = 1:N, bg="transparent")

        symbols(Stampy100_15_10$V3, Stampy100_15_10$V2,
stars=cbind(Stampy100_15_10$V4/5.773502692,Stampy100_15_10$V4/5.773502692,Stampy100_
15_10$V4/5.773502692), add=TRUE, inches=FALSE, fg = 1:N, bg="transparent")

        symbols(Novo100_15_10$V3, Novo100_15_10$V2,
stars=cbind(Novo100_15_10$V4/8.506508084,Novo100_15_10$V4/8.506508084,Novo100_15_10
$V4/8.506508084,Novo100_15_10$V4/8.506508084,Novo100_10$V4/8.506508084), add=TRUE,
inches=FALSE, fg = 1:N, bg="transparent")



      palette("default")

    })

N <- nrow(Bfast100_15_50)

    with(Bfast100_15_50, {

        palette(rainbow(N, end = 0.8))

        symbols(Bfast100_15_50$V3, Bfast100_15_50$V2, circles=Bfast100_15_50$V4/20,
inches=FALSE, fg = 1:N, bg="transparent", main="1.50% (50kb-
1mb)",font.main=2,cex.main=1,xlab="",ylab="",xlim=c(-10,160),ylim=c(-10,130))

        symbols(BWA100_15_50$V3, BWA100_15_50$V2, squares=BWA100_15_50$V4/10,
add=TRUE, inches=FALSE, fg = 1:N, bg="transparent")

        symbols(Smalt100_15_50$V3, Smalt100_15_50$V2,
rectangles=cbind(Smalt100_15_50$V4/15,Smalt100_15_50$V4/10), add=TRUE, inches=FALSE,
fg = 1:N, bg="transparent")
```

```
        symbols(Stampy100_15_50$V3, Stampy100_15_50$V2,
stars=cbind(Stampy100_15_50$V4/5.773502692,Stampy100_15_50$V4/5.773502692,Stampy100_
15_50$V4/5.773502692), add=TRUE, inches=FALSE, fg = 1:N, bg="transparent")

        symbols(Novo100_15_50$V3, Novo100_15_50$V2,
stars=cbind(Novo100_15_50$V4/8.506508084,Novo100_15_50$V4/8.506508084,Novo100_15_50
$V4/8.506508084,Novo100_15_50$V4/8.506508084,Novo100_10$V4/8.506508084), add=TRUE,
inches=FALSE, fg = 1:N, bg="transparent")



      palette("default")

  })

N <- nrow(Bfast100_3)

  with(Bfast100_3, {

        palette(rainbow(N, end = 0.8))

        symbols(Bfast100_3$V3, Bfast100_3$V2, circles=Bfast100_3$V4/20, inches=FALSE, fg =
1:N, bg="transparent", main="3% (1-5mb)",font.main=2,cex.main=1,xlab="",ylab="",xlim=c(-
10,160),ylim=c(-10,130))

        legend
(110,18,Bfast100_3$V1,col=1:N,box.col="transparent",text.col="black",fill=1:N,cex=0.5)

        legend (80,19,legend=leg.txt, box.col="transparent",col="black",
pch=c(1,0,39,2,11),cex=0.6)

        symbols(BWA100_3$V3, BWA100_3$V2, squares=BWA100_3$V4/10, add=TRUE,
inches=FALSE, fg = 1:N, bg="transparent")

        symbols(Smalt100_3$V3, Smalt100_3$V2,
rectangles=cbind(Smalt100_3$V4/15,Smalt100_3$V4/10), add=TRUE, inches=FALSE, fg = 1:N,
bg="transparent")

        symbols(Stampy100_3$V3, Stampy100_3$V2,
stars=cbind(Stampy100_3$V4/5.773502692,Stampy100_3$V4/5.773502692,Stampy100_3$V4/5.7
73502692), add=TRUE, inches=FALSE, fg = 1:N, bg="transparent")

        symbols(Novo100_3$V3, Novo100_3$V2,
stars=cbind(Novo100_3$V4/8.506508084,Novo100_3$V4/8.506508084,Novo100_3$V4/8.506508
084,Novo100_3$V4/8.506508084,Novo100_10$V4/8.506508084), add=TRUE, inches=FALSE, fg
= 1:N, bg="transparent")



      palette("default")

  })
```

```
Bfast150_5=read.table("Bfast_150_0.05")

BWA150_5=read.table("BWA_150_0.05")

Smalt150_5=read.table("Smalt_150_0.05")

Stampy150_5=read.table("Stampy_150_0.05")

Novo150_5=read.table("Novo_150_0.05")

Bfast150_10=read.table("Bfast_150_0.10")

BWA150_10=read.table("BWA_150_0.10")

Smalt150_10=read.table("Smalt_150_0.10")

Stampy150_10=read.table("Stampy_150_0.10")

Novo150_10=read.table("Novo_150_0.10")

Bfast150_15_1=read.table("Bfast_150_1.50_1")

BWA150_15_1=read.table("BWA_150_1.50_1")

Smalt150_15_1=read.table("Smalt_150_1.50_1")

Stampy150_15_1=read.table("Stampy_150_1.50_1")

Novo150_15_1=read.table("Novo_150_1.50_1")

Bfast150_15_10=read.table("Bfast_150_1.50_10")

BWA150_15_10=read.table("BWA_150_1.50_10")

Smalt150_15_10=read.table("Smalt_150_1.50_10")

Stampy150_15_10=read.table("Stampy_150_1.50_10")

Novo150_15_10=read.table("Novo_150_1.50_10")

Bfast150_15_50=read.table("Bfast_150_1.50_50")

BWA150_15_50=read.table("BWA_150_1.50_50")

Smalt150_15_50=read.table("Smalt_150_1.50_50")

Stampy150_15_50=read.table("Stampy_150_1.50_50")

Novo150_15_50=read.table("Novo_150_1.50_50")

Bfast150_3=read.table("Bfast_150_3")
```

```
BWA150_3=read.table("BWA_150_3")

Smalt150_3=read.table("Smalt_150_3")

Stampy150_3=read.table("Stampy_150_3")

Novo150_3=read.table("Novo_150_3")

par(mar=c(5,2,1,1),mfrow=c(2,3))

N <- nrow(Bfast150_5)

    with(Bfast150_5, {

          palette(rainbow(N, end = 0.8))

          symbols(Bfast150_5$V3, Bfast150_5$V2, circles=Bfast150_5$V4/20, inches=FALSE, fg = 1:N, bg="transparent", main="E              0.05% (1-10kb) ",font.main=2,cex.main=1,xlab="",ylab="",xlim=c(-10,160),ylim=c(-20,130))

          symbols(BWA150_5$V3, BWA150_5$V2, squares=BWA150_5$V4/10, add=TRUE, inches=FALSE, fg = 1:N, bg="transparent")

          symbols(Smalt150_5$V3, Smalt150_5$V2, rectangles=cbind(Smalt150_5$V4/15,Smalt150_5$V4/10), add=TRUE, inches=FALSE, fg = 1:N, bg="transparent")

          symbols(Stampy150_5$V3, Stampy150_5$V2, stars=cbind(Stampy150_5$V4/5.773502692,Stampy150_5$V4/5.773502692,Stampy150_5$V4/5.773502692), add=TRUE, inches=FALSE, fg = 1:N, bg="transparent")

          symbols(Novo150_5$V3, Novo150_5$V2, stars=cbind(Novo150_5$V4/8.506508084,Novo150_5$V4/8.506508084,Novo150_5$V4/8.506508084,Novo150_5$V4/8.506508084,Novo150_5$V4/8.506508084), add=TRUE, inches=FALSE, fg = 1:N, bg="transparent")



        palette("default")

    })

N <- nrow(Bfast150_10)

    with(Bfast150_10, {

          palette(rainbow(N, end = 0.8))

          symbols(Bfast150_10$V3, Bfast150_10$V2, circles=Bfast150_10$V4/20, inches=FALSE, fg = 1:N, bg="transparent", main="0.10% (10-50kb)",font.main=2,cex.main=1,xlab="",ylab="",xlim=c(-10,160),ylim=c(-20,130))
```

```
symbols(BWA150_10$V3, BWA150_10$V2, squares=BWA150_10$V4/10, add=TRUE, inches=FALSE, fg = 1:N, bg="transparent")

symbols(Smalt150_10$V3, Smalt150_10$V2, rectangles=cbind(Smalt150_10$V4/15,Smalt150_10$V4/10), add=TRUE, inches=FALSE, fg = 1:N, bg="transparent")

symbols(Stampy150_10$V3, Stampy150_10$V2, stars=cbind(Stampy150_10$V4/5.773502692,Stampy150_10$V4/5.773502692,Stampy150_10$V4/5.773502692), add=TRUE, inches=FALSE, fg = 1:N, bg="transparent")

symbols(Novo150_10$V3, Novo150_10$V2, stars=cbind(Novo150_10$V4/8.506508084,Novo150_10$V4/8.506508084,Novo150_10$V4/8.506508084,Novo150_10$V4/8.506508084,Novo150_10$V4/8.506508084), add=TRUE, inches=FALSE, fg = 1:N, bg="transparent")



palette("default")

})

N <- nrow(Bfast150_15_1)

with(Bfast150_15_1, {

palette(rainbow(N, end = 0.8))

symbols(Bfast150_15_1$V3, Bfast150_15_1$V2, circles=Bfast150_15_1$V4/20, inches=FALSE, fg = 1:N, bg="transparent", main="1.50% (1-10kb)",font.main=2,cex.main=1,xlab="",ylab="",xlim=c(-10,160),ylim=c(-20,130))

symbols(BWA150_15_1$V3, BWA150_15_1$V2, squares=BWA150_15_1$V4/10, add=TRUE, inches=FALSE, fg = 1:N, bg="transparent")

symbols(Smalt150_15_1$V3, Smalt150_15_1$V2, rectangles=cbind(Smalt150_15_1$V4/15,Smalt150_15_1$V4/10), add=TRUE, inches=FALSE, fg = 1:N, bg="transparent")

symbols(Stampy150_15_1$V3, Stampy150_15_1$V2, stars=cbind(Stampy150_15_1$V4/5.773502692,Stampy150_15_1$V4/5.773502692,Stampy150_15_1$V4/5.773502692), add=TRUE, inches=FALSE, fg = 1:N, bg="transparent")

symbols(Novo150_15_1$V3, Novo150_15_1$V2, stars=cbind(Novo150_15_1$V4/8.506508084,Novo150_15_1$V4/8.506508084,Novo150_15_1$V4/8.506508084,Novo150_15_1$V4/8.506508084,Novo150_10$V4/8.506508084), add=TRUE, inches=FALSE, fg = 1:N, bg="transparent")



palette("default")
```

```
    })
N <- nrow(Bfast150_15_10)
    with(Bfast150_15_10, {
        palette(rainbow(N, end = 0.8))
        symbols(Bfast150_15_10$V3, Bfast150_15_10$V2, circles=Bfast150_15_10$V4/20, inches=FALSE, fg = 1:N, bg="transparent", main="1.50% (10-50kb)",font.main=2,cex.main=1,xlab="",ylab="",sub="Read length (150 nts) ",font.sub=2,cex.sub=1,xlim=c(-10,160),ylim=c(-20,130))
        symbols(BWA150_15_10$V3, BWA150_15_10$V2, squares=BWA150_15_10$V4/10, add=TRUE, inches=FALSE, fg = 1:N, bg="transparent")
        symbols(Smalt150_15_10$V3, Smalt150_15_10$V2, rectangles=cbind(Smalt150_15_10$V4/15,Smalt150_15_10$V4/10), add=TRUE, inches=FALSE, fg = 1:N, bg="transparent")
        symbols(Stampy150_15_10$V3, Stampy150_15_10$V2, stars=cbind(Stampy150_15_10$V4/5.773502692,Stampy150_15_10$V4/5.773502692,Stampy150_15_10$V4/5.773502692), add=TRUE, inches=FALSE, fg = 1:N, bg="transparent")
        symbols(Novo150_15_10$V3, Novo150_15_10$V2, stars=cbind(Novo150_15_10$V4/8.506508084,Novo150_15_10$V4/8.506508084,Novo150_15_10$V4/8.506508084,Novo150_15_10$V4/8.506508084,Novo150_10$V4/8.506508084), add=TRUE, inches=FALSE, fg = 1:N, bg="transparent")


      palette("default")
    })
N <- nrow(Bfast150_15_50)
    with(Bfast150_15_50, {
        palette(rainbow(N, end = 0.8))
        symbols(Bfast150_15_50$V3, Bfast150_15_50$V2, circles=Bfast150_15_50$V4/20, inches=FALSE, fg = 1:N, bg="transparent", main="1.50% (50kb-1mb)",font.main=2,cex.main=1,xlab="",ylab="",xlim=c(-10,160),ylim=c(-20,130))
        symbols(BWA150_15_50$V3, BWA150_15_50$V2, squares=BWA150_15_50$V4/10, add=TRUE, inches=FALSE, fg = 1:N, bg="transparent")
        symbols(Smalt150_15_50$V3, Smalt150_15_50$V2, rectangles=cbind(Smalt150_15_50$V4/15,Smalt150_15_50$V4/10), add=TRUE, inches=FALSE, fg = 1:N, bg="transparent")
```

```
symbols(Stampy150_15_50$V3, Stampy150_15_50$V2,
stars=cbind(Stampy150_15_50$V4/5.773502692,Stampy150_15_50$V4/5.773502692,Stampy150_15_50$V4/5.773502692), add=TRUE, inches=FALSE, fg = 1:N, bg="transparent")

symbols(Novo150_15_50$V3, Novo150_15_50$V2,
stars=cbind(Novo150_15_50$V4/8.506508084,Novo150_15_50$V4/8.506508084,Novo150_15_50$V4/8.506508084,Novo150_15_50$V4/8.506508084,Novo150_10$V4/8.506508084), add=TRUE, inches=FALSE, fg = 1:N, bg="transparent")



palette("default")

})

N <- nrow(Bfast150_3)

with(Bfast150_3, {

palette(rainbow(N, end = 0.8))

symbols(Bfast150_3$V3, Bfast150_3$V2, circles=Bfast150_3$V4/20, inches=FALSE, fg = 1:N, bg="transparent", main="3% (1-5mb)",font.main=2,cex.main=1,xlab="",ylab="",xlim=c(-10,160),ylim=c(-20,130))

legend
(110,6,Bfast100_3$V1,col=1:N,box.col="transparent",text.col="black",fill=1:N,cex=0.5)

legend (80,7,legend=leg.txt, box.col="transparent",col="black",
pch=c(1,0,39,2,11),cex=0.6)

symbols(BWA150_3$V3, BWA150_3$V2, squares=BWA150_3$V4/10, add=TRUE, inches=FALSE, fg = 1:N, bg="transparent")

symbols(Smalt150_3$V3, Smalt150_3$V2,
rectangles=cbind(Smalt150_3$V4/15,Smalt150_3$V4/10), add=TRUE, inches=FALSE, fg = 1:N, bg="transparent")

symbols(Stampy150_3$V3, Stampy150_3$V2,
stars=cbind(Stampy150_3$V4/5.773502692,Stampy150_3$V4/5.773502692,Stampy150_3$V4/5.773502692), add=TRUE, inches=FALSE, fg = 1:N, bg="transparent")

symbols(Novo150_3$V3, Novo150_3$V2,
stars=cbind(Novo150_3$V4/8.506508084,Novo150_3$V4/8.506508084,Novo150_3$V4/8.506508084,Novo150_3$V4/8.506508084,Novo150_10$V4/8.506508084), add=TRUE, inches=FALSE, fg = 1:N, bg="transparent")



palette("default")
```

})
